# Mapping the function of MicroRNAs as a critical regulator of tumor-immune cell communication in breast cancer and potential treatment strategies

**DOI:** 10.3389/fcell.2024.1390704

**Published:** 2024-04-25

**Authors:** Aimi Syamima Abdul Manap, Aini Athirah Wisham, Fei Wen Wong, Huda Raihanah Ahmad Najmi, Zhi Fei Ng, Rubaiyat Siddique Diba

**Affiliations:** ^1^ Department of Biomedical Science, College of Veterinary Medicine, King Faisal University, Al-Ahsa, Saudi Arabia; ^2^ Faculty of Biosciences, MAHSA University, Kuala Langat, Selangor, Malaysia

**Keywords:** breast cancer, microRNA, miRNA, tumor-immune cells, tumor suppressor, oncogene

## Abstract

Among women, breast cancer ranks as the most prevalent form of cancer, and the presence of metastases significantly reduces prognosis and diminishes overall survival rates. Gaining insights into the biological mechanisms governing the conversion of cancer cells, their subsequent spread to other areas of the body, and the immune system’s monitoring of tumor growth will contribute to the advancement of more efficient and targeted therapies. MicroRNAs (miRNAs) play a critical role in the interaction between tumor cells and immune cells, facilitating tumor cells’ evasion of the immune system and promoting cancer progression. Additionally, miRNAs also influence metastasis formation, including the establishment of metastatic sites and the transformation of tumor cells into migratory phenotypes. Specifically, dysregulated expression of these genes has been associated with abnormal expression of oncogenes and tumor suppressor genes, thereby facilitating tumor development. This study aims to provide a concise overview of the significance and function of miRNAs in breast cancer, focusing on their involvement as tumor suppressors in the antitumor immune response and as oncogenes in metastasis formation. Furthermore, miRNAs hold tremendous potential as targets for gene therapy due to their ability to modulate specific pathways that can either promote or suppress carcinogenesis. This perspective highlights the latest strategies developed for miRNA-based therapies.

## Introduction

**FIGURE 1:**
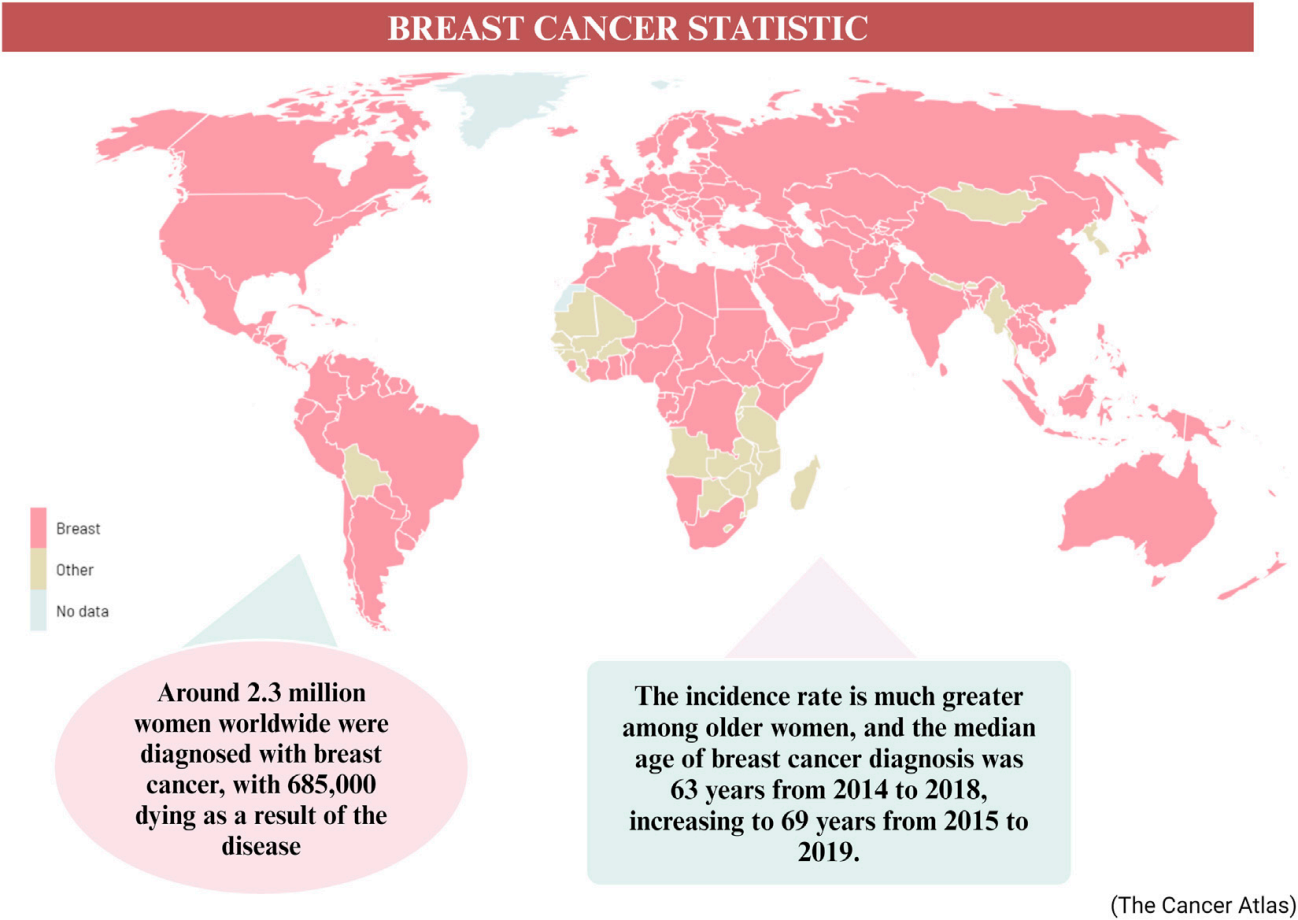
The worldwide statistic of breast cancer cases in 2020–2040 (Arnold et al., 2022).

More than one in ten new cases of cancer identified in women are related to breast cancer, making it the most common cancer diagnosed in such a group (Alkabban and Ferguson, 2023). In 2020, approximately 2.3 million women worldwide were diagnosed with breast cancer, resulting in 685,000 deaths related to the disease (Sung et al., 2021). The incidence rate tends to be higher among older women, with a median age of breast cancer diagnosis recorded as 63 years from 2014 to 2018, and increasing to 69 years from 2015 to 2019 (Cao et al., 2021) (Figure 1). Breast cancer is predominantly an incurable metastatic cancer that frequently spreads to distant organs such as the liver, brain, lungs, and bones. Early detection plays a crucial role in achieving a favorable prognosis and higher survival rates (Sun et al., 2017). Global data on cancer incidence rates reveal that breast cancer accounted for 49.3% of all diagnosed cases in women in 2020, surpassing any other cancer type (Lee et al., 2019). Given the existence of multiple biological subtypes, each characterized by unique molecular profiles and clinicopathological characteristics, breast cancer is often regarded as a diverse group of illnesses (>100). Gene expression profiling has allowed the distinction between receptor-positive subtypes (Luminal A, B, Normal like, and HER-2 positive) and receptor-negative subtypes (such as TNBC or Basal-like), in addition to histological subtypes (Kashyap et al., 2022). Breast cancer screening significantly improves survival rates by enhancing the likelihood of early detection and prompt treatment of malignant tumors. The recommended screening modalities for breast cancer include breast self-examination, clinical breast examination, and mammography for women aged forty (Sebastian-delaCruz et al., 2021) and above. While developing nations emphasize the importance of breast self-examination as a pivotal strategy for early breast cancer identification, inadequate funding hinders the implementation of mammography screening (Lee et al., 2019). Presently, the three primary methods used for treating breast cancer are radiation therapy, chemotherapy, and surgery.

In the past, breast cancer was commonly perceived as a malignancy with minimal immunological responses. The immunological landscape surrounding breast cancer comprises a diverse array of cells and cytokines, some with anti-tumor properties while others possess pro-tumor or immunosuppressive characteristics. Persistent inflammation driven by these cells and cytokines can hasten the progression of breast cancer. Among immune system components, such as NK cells and CTLs, there exists a focus on combating cancer by targeting breast cancer cells. However, various elements including tregs, macrophages, MDSCs, and T-helper cells contribute to breast cancer advancement through mechanisms such as promoting metastasis, secreting proinflammatory cytokines, and attenuating the function of cytotoxic T-cells. B-cells may exhibit a protumorigenic role by reducing antitumor immunity, whereas they can also release antibodies that neutralize tumors, thus assuming an antitumorigenic role (Amens et al., 2021a). Inflammation can severely damage breast tissue and facilitate breast cancer development by increasing proinflammatory cytokines like TNF-α and interleukins (Amens et al., 2021a) Tumor cells have shown resilience against chemotherapy through various mechanisms. These mechanisms can be classified into six categories: elevated expression of specific ATP-binding cassette transport proteins, evasion of cell death, enhancement of DNA repair pathways, relevant mutations in cellular targets, increased resilience to adverse environments, and biotransformation of anticancer drugs (Miraghel et al., 2022). When it comes to treatment considerations for breast cancer, several factors are taken into account, including the expression of HER2, progesterone receptor (PR), and estrogen receptor (ER), along with other patient- and tumor-specific characteristics. The protein HER2 is encoded by the erythroblastic oncogene B (ERBB2) gene. Oncogene amplification, leading to the overexpression of HER2, fosters the proliferation of cancer cells and contributes to the emergence of various subtypes of breast cancer (van den Ende et al., 2022).

Reflecting on historical limits, the care of breast cancer has significantly evolved throughout time, leading to a more sophisticated approach. When it comes to early diagnosis, previous screening methods were less advanced and less generally available, resulting in delayed discovery of breast cancer in many cases. However, modern imaging techniques such as digital mammography, ultrasound, MRI, and 3D mammography enable earlier and more accurate identification of breast cancer, hence improving survival rates and treatment outcomes. Aside from that, survival rates for breast cancer were poorer due to fewer treatment options, late-stage diagnosis, and less effective medicines. However, nowadays, the breast cancer survival rates have considerably improved thanks to advances in early identification, tailored treatments, and supportive care, resulting in better long-term results and increasing chances of cure, particularly when the cancer is diagnosed early (Li et al., 2021a). For examples, in recent years, extensive research has focused on various non-invasive biomarkers, including circulating cell-free or exosomal non-coding RNAs, proteins (such as tumor-associated autoantibodies, carcinoembryonic antigen, carbohydrate antigen, tissue polypeptide-specific antigen, etc.), polygenic risk scores involving single nucleotide polymorphisms, and cell-free DNA. Among these biomarkers, circulating cell-free miRNAs have garnered attention. MiRNAs are small non-coding RNAs measuring around 17 to 25 nucleotides, and they play a crucial role in regulating gene expression at a post-transcriptional level. Acting as master regulators, miRNAs can repress translation or degrade messenger RNAs (mRNAs), thus controlling gene expression. They are essential components of biological regulatory processes involved in development, host-pathogen interactions, cell differentiation, proliferation, apoptosis, metabolism, and cancer across various organisms (Maldonado et al., 2022). Mature miRNAs can be exported from cells and are primarily located within the cytoplasm. Some miRNAs persistently exist in bodily fluids like serum, plasma, saliva, or urine due to their interaction with exosomes or RNA-binding proteins, protecting them from degradation. Changes in the levels of these diagnostic circulating miRNAs have been observed even before the detection of tumors using conventional diagnostic tools. Breast cancer research and knowledge have advanced substantially. Breast cancer is now recognized as a diverse disease with distinct subtypes based on molecular features. Aside from that, advancements in treatment procedures increase the identification of specific mutations, gene amplifications, and expression patterns, resulting in personalized therapy regimens tailored to individual patients (O’Day and Lal, 2010). The function of miRNAs in treatment approach has a significant impact on patient survival. The function can affect a variety of biological processes, including cell proliferation, differentiation, apoptosis, metabolism, and immunological response. It regulates several molecular pathways that contribute to the growth of breast cancer. For example, depending on their target genes, miRNAs can serve as oncogenes (oncomiRs) or tumor suppressors. OncomiRs enhance cancer cell proliferation, survival, and invasion by targeting tumor suppressor genes or apoptosis regulators, whereas miRNAs regulate angiogenesis by targeting genes involved in VEGF signaling and angiogenic pathways. They also affect metastasis by altering genes related to cell adhesion, migration, and invasion, encouraging the spread of cancer cells to distant organs. MiRNAs regulate hormone receptor signaling in breast cancer. MiRNAs can target estrogen receptor alpha (ERα) or PR, impacting hormone responsiveness and treatment results in hormone receptor-positive breast tumors. Finally, changes in the expression of various miRNAs are linked to different breast cancer subtypes, clinical stages, and patient outcomes. MiRNAs can be used as diagnostic biomarkers for early detection, as well as prognostic indications for disease progression, metastasis, and treatment response (Loh et al., 2019). Thus, circulating cell-free miRNAs may have greater utility than other listed biomarkers in detecting early-stage breast cancer (Sehovic et al., 2022). The objective of this study is to provide a concise overview of the importance and role of miRNAs in breast cancer. It will discuss their involvement in the antitumor immune response as tumor suppressors and their participation in metastasis as oncogenes. Furthermore, miRNAs hold promise as therapeutic targets for gene therapy due to their ability to influence specific pathways that either promote or inhibit carcinogenesis. This perspective will emphasize the latest strategies developed for therapeutics utilizing miRNA.

## Breast cancer

Breast cancer, with an estimated 2.3 million new cases worldwide, currently stands as one of the most prevalent malignancies across the globe and ranks as the fifth leading cause of cancer-related deaths. Among women worldwide, breast cancer takes the foremost position in terms of cancer-related mortality (Łukasiewicz et al., 2021a). Breast cancer is a complex disease influenced by various factors. While it affects populations globally, significant regional differences in incidence, mortality, and survival rates exist. These variations can be attributed to factors such as age, estrogen levels, lifestyle choices, and genetics (Momenimovahed and Salehiniya, 2019). Age is a crucial risk factor for breast cancer, and its incidence strongly correlates with advancing age. Being a woman and growing older are the two primary variables affecting the risk, with the majority of cases occurring in women aged 50 or older (Centers for Disease Control and Prevention, 2023). However, extensive research has focused specifically on young women, who often experience more aggressive forms of the disease, receive intensive therapies, and face concerns of lower survival rates (Plichta et al., 2020). The role of estrogen, categorized into endogenous and exogenous estrogens, is significant in breast cancer risk. Endogenous estrogen, particularly in estrogen receptor-positive subtypes that account for 70% of cases after menopause, plays a major role in breast cancer development. Breast adipose tissue in cancer patients exhibits significantly elevated expressions of CYP19 and aromatase, leading to increased estrogen production stimulated by tumor-derived substances such as prostaglandins released by fibroblasts, infiltrating lymphocytes, or epithelial cells (Parida and Sharma, 2019). Additionally, sedentary lifestyle, regular alcohol consumption, and abdominal obesity increase the risk of breast cancer, regardless of menopausal status. In premenopausal women, abdominal obesity is associated with a 3.3-fold increase in risk. Smoking, both current and prior, raises the risk of postmenopausal breast cancer (McCarthy et al., 2021). Finally, genetic influences contribute to breast cancer. Germline mutations in known susceptibility genes account for 5%–10% of cases and are categorized as “high-penetrance,” “moderate-penetrance,” and “low-penetrance” based on their impact and recurrence. Mutations in genes such as BRCA1 and BRCA2 account for 10%–15% of heritable breast cancer cases, while other high-risk genes, including TP53, CDH1, phosphate and tensin homolog (PTEN), and STK11, also lead to breast cancer development. The NF1 gene, associated with neuroectodermal disorders and exhibiting wide variation in gene expression due to autosomal dominant inheritance, is another gene implicated in invasive breast cancer (Naeem et al., 2019).

**FIGURE 2:**
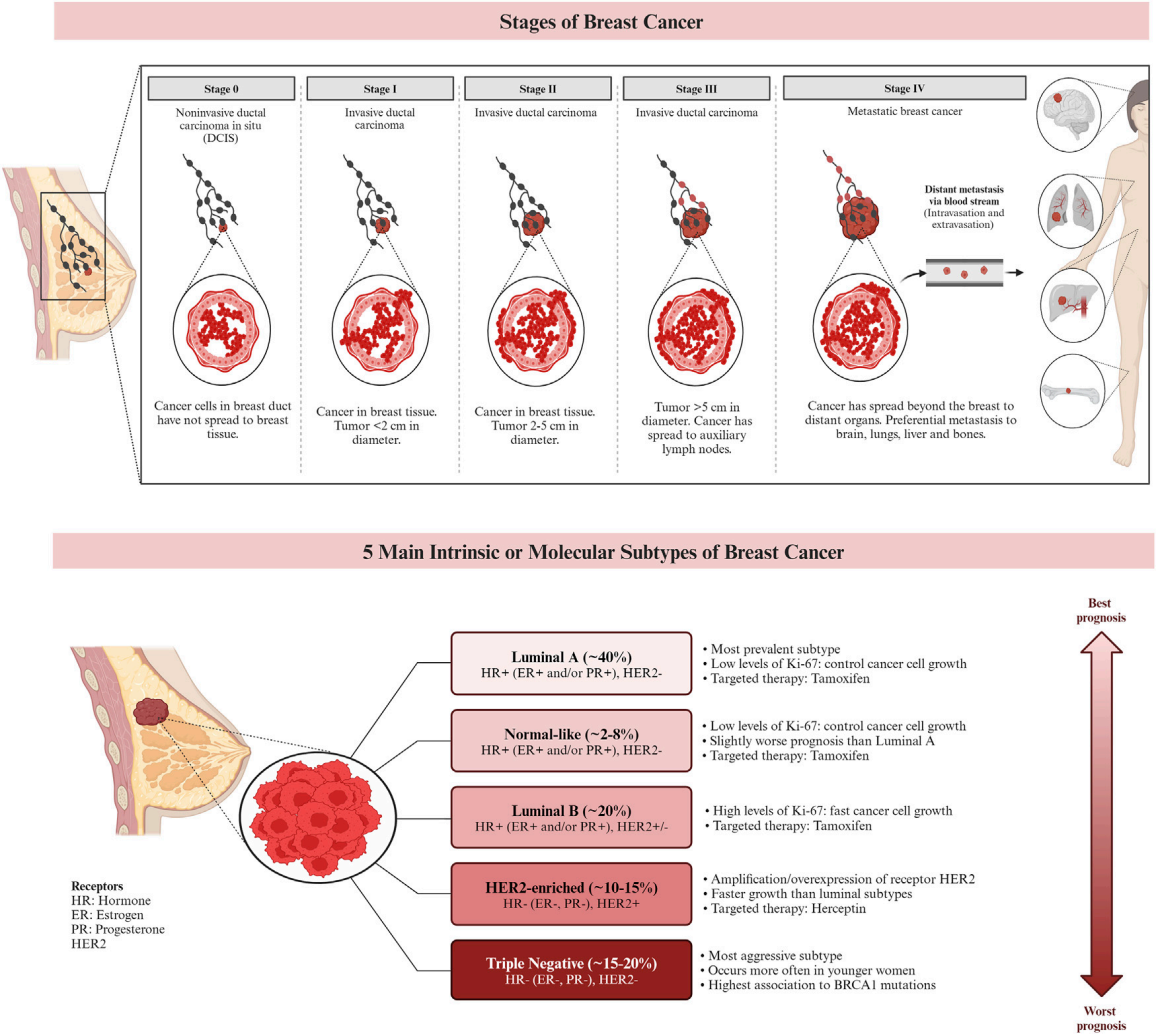
The stages and the subtypes of breast cancer. Breast cancer subtypes can be categorized based on their expression of hormone receptors (estrogen receptor (ER) and progesterone receptor (PR)), the proliferation marker Ki-67, and the receptor tyrosine kinase HER2. Targeted therapies, such as Herceptin (aimed at the HER2 protein) and Tamoxifen (aimed at the ER), can be used in the treatment of certain breast cancer subtypes. Prognosis varies based on the subtype of breast cancer. (Larsen et al., 2013; Chen et al., 2018; De Cicco et al., 2019; Hashmi et al., 2019).

Genetic alterations and DNA damage, which can be influenced by estrogen exposure, are known to contribute to the development of breast cancer. Inherited genes such as BRCA1 and BRCA2 have been associated with an increased risk of cancer. As a result, individuals with a family history of ovarian or breast cancer have a higher likelihood of developing breast cancer themselves. In a healthy individual, the immune system typically targets cells with abnormal DNA or development. However, in cases of breast cancer, this surveillance system may fail, allowing the tumor to grow and spread (Alkabban and Ferguson, 2023). Currently, breast cancer is classified using histopathologic characteristics, molecular characterization, and immunohistochemistry (IHC). Invasive ductal carcinoma and invasive lobular carcinoma are the most common histologic subtypes, accounting for 80%–85%, and 10%–15% of all cases of invasive breast cancer, respectively. Other less common histologic cancer subtypes make up less than 1% of invasive breast cancers. The characterization of breast cancer through IHC plays a crucial role in determining treatment strategies and predicting prognosis (Bhattacharyya et al., 2020). IHC is frequently employed to classify breast cancer subtypes based on the expression of specific markers, including the ER, PR, and HER2. Approximately 73% of breast cancers are ER/PR + HER2− tumors, which represent the most common subtype. It is worth noting that these tumors can also be PR positive. Research suggests that the etiologies of different breast cancer subtypes may vary (McCarthy et al., 2021). Figure 2 demonstrates the stages and subtypes of breast cancer.

## Subtypes of breast cancer

### Triple negative breast cancer (TNBC)

Breast cancer subtype known as triple negative breast cancer (TNBC) is a malignant and complex one that presents with challenges for therapeutic targeting due to its absence of expression of the ER, PR, and HER2 receptors. From a biological perspective, TNBC tumors typically exhibit increased aggressiveness and size, along with a higher oncological grade and lymph node metastases. Although metaplastic cancers, which may exhibit squamous or spindle cell differentiation, medullary-like cancers with a prominent lymphocytic infiltrate, and uncommon special type cancers like adenoid cystic carcinoma are the most common histologies observed in TNBC (Cao and Niu, 2020).

### Luminal breast cancer

ER-positive tumors known as luminal breast cancers account for over 70% of all instances of breast cancer. There are two primary biological processes that differentiate Luminal-like cancers into Luminal A and B subtypes: proliferation-related pathways and luminal-regulated pathways. The presence of ER and/or PR and the lack of HER2 are characteristics of luminal A cancers. The ER transcription factors in this subtype activate genes whose expression is typical of the luminal epithelium lining the mammary ducts. Additionally, it exhibits decreased expression of genes involved in cell division. Luminal B tumors, on the other hand, have a worse prognosis and are higher grade. In addition to being ER positive, they could also be PR negative, HER2, or both. Furthermore, genes linked to proliferation are highly expressed in it. This subtype expresses less of the PR and FOXA1 genes and proteins, which are typical of the luminal epithelium, but not the ER (Łukasiewicz et al., 2021b).

### HER2-enriched breast cancer

**FIGURE 3:**
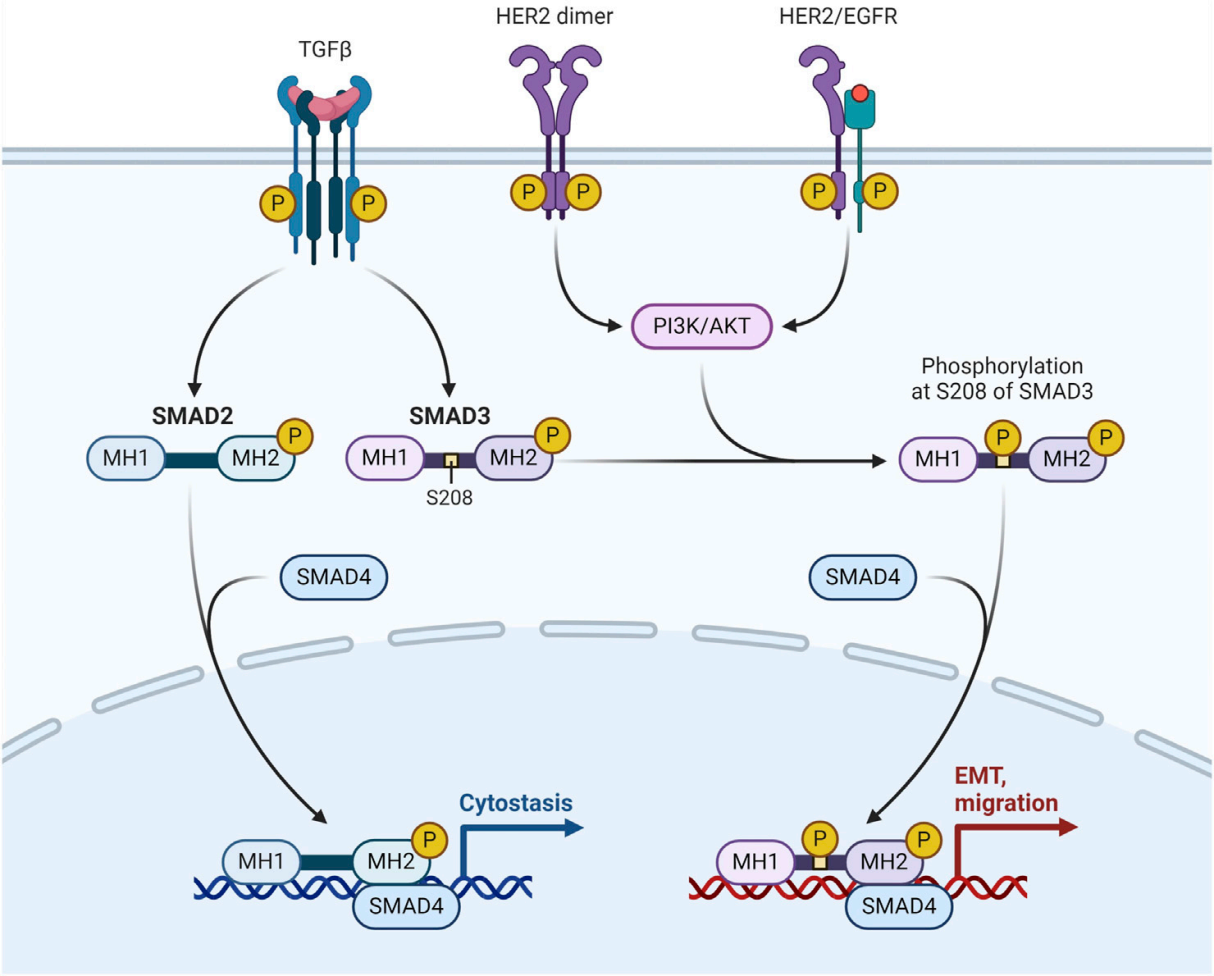
HER-2/EGFR signaling pathway in breast cancer. HER-2/EGFR signaling switches TGFβ function from inducing antiproliferation to promoting breast cancer development via the AKT-mediated phosphorylation of Smad3 at S208 in the linker (Huang F. et al., 2018).

The transmembrane tyrosine kinase receptor encoded by the HER2, often referred to as HER2/neu and Erb-B2, binds to its extracellular signal to start a cascade that regulates cell proliferation, differentiation, and survival. Adequate tumor development and unfavourable clinical prognosis are caused by overexpression of the HER2 protein and/or HER2 gene amplification in 12%–20% of all breast cancer cases. They have modest levels of ER expression and overexpression of the Erb-B2 oncogene. It's interesting to note that the HER2-enriched subtype does not exhibit upregulation of proliferation genes such Ki-67 and proliferating cell nuclear antigen (Conant and Soo, 2021) (Figure 3).

### Claudin-low breast cancer

The most notable gene expression traits that characterize the claudin-low breast cancer subtype include stem cell-like/less differentiated gene expression patterns, high expression of epithelial-mesenchymal transition genes, and low expression of cell-cell adhesion genes. Claudin-low tumors have significant immune and stromal cell infiltration in addition to these gene expression characteristics, but they differ greatly in many other respects. According to reports, most claudin-low breast cancers are triple negative—they do not express the ER, PR, or HER2. These tumors are linked to a poor prognosis (Bergholtz et al., 2020). The poor expression of essential cell-cell adhesion molecules, such as occludin, E-cadherin, and claudins 3, 4, and 7, is a defining characteristic of the claudin-low subtype. Because of their extreme enrichment in stem cell and mesenchymal characteristics, these tumors are regarded as the most primordial forms of breast cancer (Pommier et al., 2020).

Breast cancer patients at stages I, II, III, and IV have 5-year survival rates of 100%, 93%, 72%, and 26%, respectively. While the death rate from breast cancer has decreased as a result of early identification and detection, more advancements in prevention, detection, and treatment are desperately needed to improve the prognosis and survival of breast cancer patients. MiRNAs have garnered significant attention in recent times due to their regulatory role in the development, advancement, and spread of breast cancer. Moreover, there exists a strong correlation between the expression level of specific miRNAs and the morphological characteristics, immunohistochemical profiles, histopathological parameters, clinical outcomes, prognosis, and response to treatment of breast cancer It is noteworthy that approximately half of the human miRNA-encoding genes are located in regions associated with cancer or fragile chromosomal sites. Various studies have demonstrated altered expression of miRNAs in breast cancer since the initial discovery of miRNA dysregulation in breast cancer in 2005 (Loh et al., 2019).

### MiRNAs and the mechanism

MiRNAs have become a focal point in both fundamental and applied biomedical research due to their impact on gene expression, widespread presence in bodily tissues and fluids, and potential as disease biomarkers (Citron et al., 2017; Yoon et al., 2020). The primary mechanism by which miRNAs exert their influence is through the targeting of messenger RNA (mRNA) (Pu et al., 2019). Alterations in miRNA expression can significantly impact target regulation, thereby influencing cellular homeostasis. Consequently, the relative levels of miRNA, and subsequently mRNA, play a pivotal role in processes such as carcinogenesis and other diseases (Hill and Tran, 2021).

## Mechanism involved in MiRNAs and its Pathological functions

### Biogenesis

MiRNA biogenesis denotes the intricate process by which these diminutive RNA molecules are synthesized within a cell. This intricate pathway involves several sequential stages, commencing with the transcription of miRNA genes and culminating in the production of mature miRNAs capable of finely modulating gene expression. The maturation of miRNAs is facilitated by the activity of RNAse III enzymes, namely, Drosha and Dicer in animals, and DCL in plants. Following cropping and exportation to the cytoplasm, miRNAs undergo further processing, being diced into smaller fragments, which are subsequently incorporated into Argonaute proteins. Notably, Drosha, in conjunction with Di George syndrome critical region gene 8 (DGCR8), assumes the role of a molecular ruler, precisely measuring the cleavage site during this intricate miRNA biogenesis process (Hajarnis et al., 2015). The biogenesis of miRNAs initiates with the transcription of dedicated information units by RNA polymerase II, progressing through successive maturation steps to produce the final functional regulators (Treiber et al., 2019). Two distinct groups of enzymes participate in miRNA biogenesis: processors, comprising RNA endonucleases cleaving miRNA precursors in the nucleus (Drosha) and the cytoplasm (Dicer), and effectors, encompassing the Argonaute family (Ago protein) and proteins orchestrating post-transcriptional regulatory effects on mRNA targets (RISC complex) (Daugaard and Hansen, 2017). The overall pathway includes the transport system responsible for exporting miRNA precursors from the nucleus to the cytoplasm (Exportin-5) and auxiliary proteins modulating specific steps, such as TRBP and DGCR8. Nuclear processors are exclusive to miRNA biosynthesis, while the cytoplasmic branch of the pathway may intersect with other regulatory mechanisms like RNA-mediated interference (RNAi). Human miRNA biogenesis involves a two-step process with nuclear and cytoplasmic cleavage events. In the nucleus, miRNAs are transcribed as long transcripts termed pri-miRNA, either by their independent promoters or by sharing promoters with their host gene (Creugny et al., 2018). This transcription is predominantly executed by RNA polymerase II (Schier and Taatjes, 2020), although certain virus-encoded miRNAs are transcribed by RNA polymerase III (Nanbo et al., 2021). Recognition of a stem-loop structure within the pri-miRNA occurs through the action of the RNase III Drosha and its cofactor DGCR8 (Nguyen et al., 2020). This intricate process ensures the formation of mature miRNAs essential for post-transcriptional gene regulation.

**FIGURE 4:**
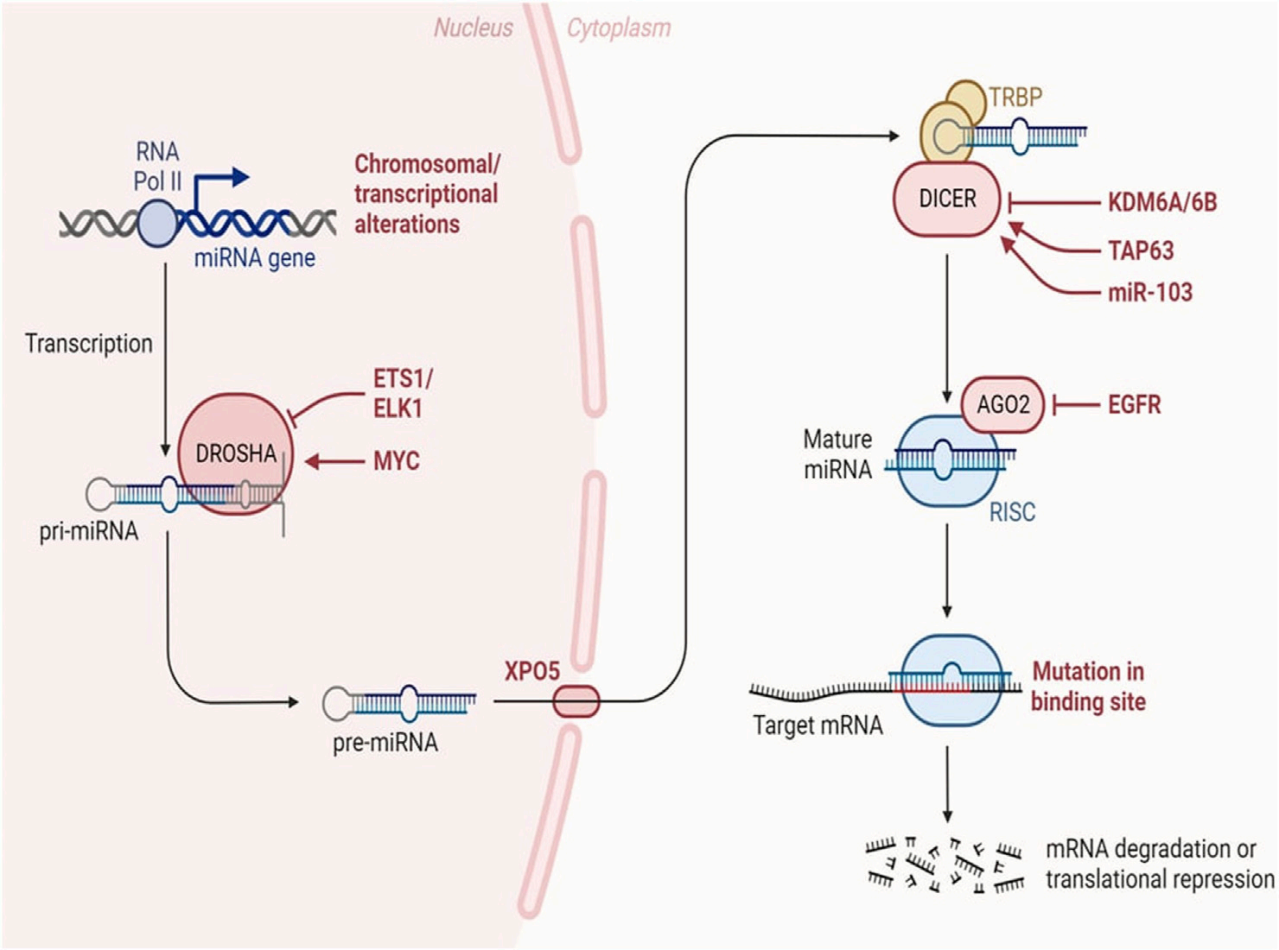
MiRNA biogenesis in cancer. Changes occurring throughout the process of miRNA synthesis can impact the accessibility of the target mRNA. Adapted from Lin et al. (2015) (Lin and Gregory, 2015).

The miRNA biogenesis pathway is depicted schematically, encompassing the key enzymes and cellular compartments involved. Eukaryotic genomes harbour transcriptional units responsible for generating miRNAs, which can be found in various genomic regions, including intergenic spaces, coding genes, and non-coding genes. Following transcription, primary miRNAs adopt a characteristic hairpin-loop secondary structure, recognized and excised by the microprocessor complex comprising DGCR8 and Drosha. Subsequently, the resulting precursor miRNAs undergo exportation to the cytoplasm, where the Dicer nuclease processes them into double-stranded RNA. The mature miRNA chain is then selected by Ago2 and incorporated into the RNA-induced silencing complex, initiating its regulatory function. In this intricate process, transcriptional units within the genome give rise to miRNAs, which exhibit a diverse genomic distribution. The microprocessor complex, involving DGCR8 and Drosha, plays a crucial role in recognizing and cleaving the hairpin-loop structure of primary miRNAs. Following this nuclear processing, precursor miRNAs are transported to the cytoplasm, where Dicer further refines them into double-stranded RNA. The mature miRNA is subsequently chosen by Ago2 and integrated into the RNA-induced silencing complex, where it becomes a pivotal player in post-transcriptional gene regulation (Figure 4).

### Regulation of gene expression

MiRNAs orchestrate post-transcriptional gene regulation by binding to target messenger RNAs (mRNAs), inducing either translational repression or mRNA degradation (Sebastian-delaCruz et al., 2021; Rani and Sengar, 2022). This regulatory mechanism is pivotal for precise modulation of gene expression, contributing to the maintenance of cellular homeostasis (Fernández-Hernando and Suárez, 2018).

MiRNAs regulate gene expression post-transcriptionally in a sequence-specific manner, with significant biological impact (Acuña et al., 2020). Research has delved into understanding the mechanisms of miRNA-mediated post-transcriptional regulation, using *in vitro*, *in vivo*, and cell-free extracts, along with bioinformatics tools predicting miRNA regulation of nearly 30% of protein-coding genes in mammalian cells (Boivin et al., 2018). MiRNAs influence translation by inhibiting initiation, preventing ribosomal association, and blocking elongation (Oliveto et al., 2017; Ahmed et al., 2018; Naeli et al., 2023). They also regulate mRNA stability by recruiting decay machinery components, leading to deadenylation or decapping followed by exonuclease activity (Frédérick and Simard, 2022; Passmore and Coller, 2022). Additionally, miRNAs are secreted as exosomes or into extracellular fluids, suggesting potential roles in intercellular communication and as disease biomarkers, including infectious diseases (Acuña et al., 2020). Figure 4 shows the how the alterations throughout miRNA biogenesis can affect availability of target mRNA.

## Disease associations in cancer (OncomiRs)

**FIGURE 5:**
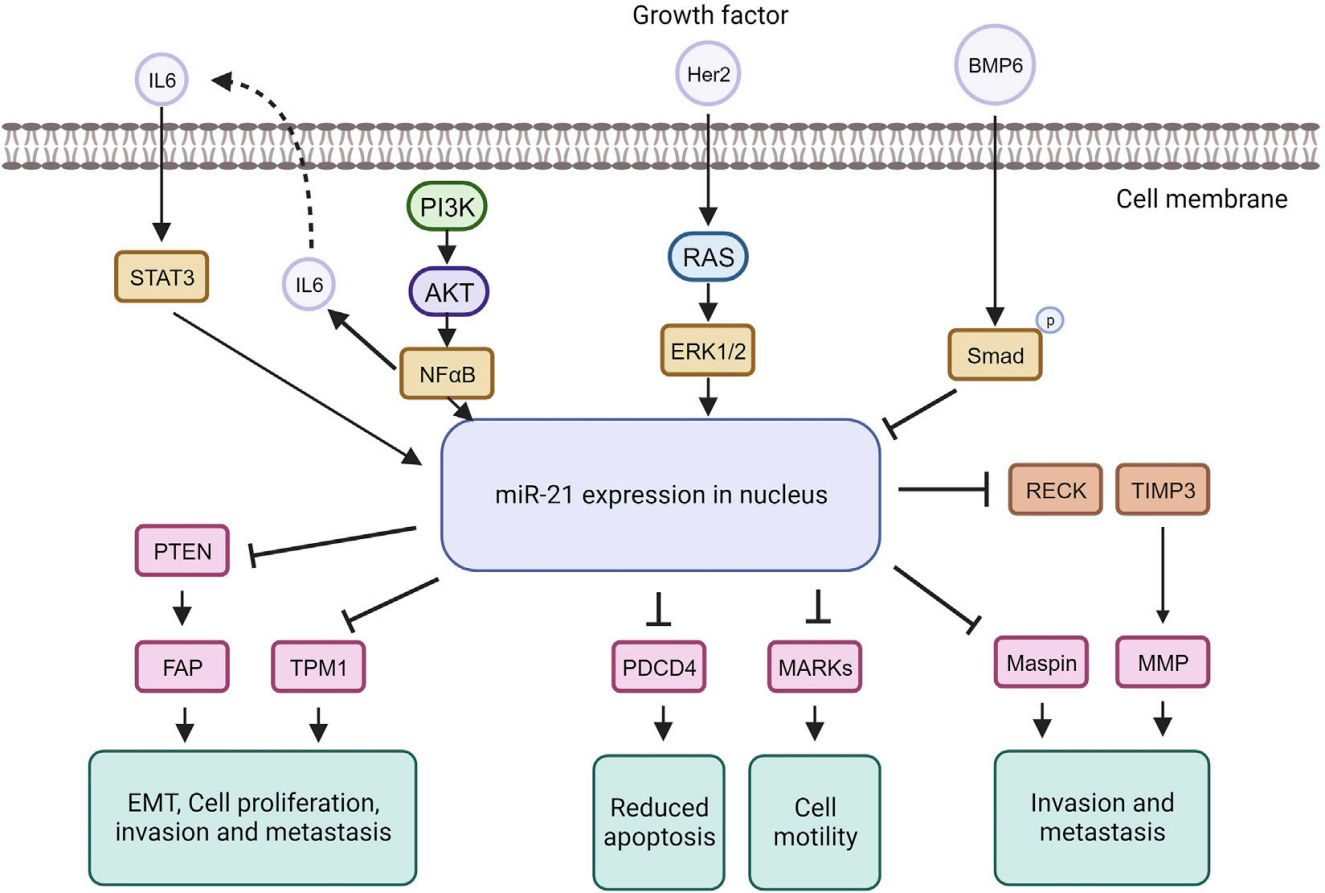
MiRNA-21 targets and their function in tumorigenic pathways.

MiRNA dysregulation is commonly linked to cancer, where some function as oncogenes (oncomiRs), promoting tumor formation, while others act as tumor suppressors (O’Bryan et al., 2017). In breast cancer, miR-21, an extensively studied oncomiR, is often elevated, leading to increased cell proliferation and invasion. MiR-21 exerts its oncogenic functions by targeting genes like TPM1, PDCD4, PTEN, and Smad7, enhancing tumor cell growth and inhibiting apoptosis (Zhang Y. et al., 2020; Najjary et al., 2020; Shen et al., 2020) (Figure 5).

The tumor suppressor TPM1 is suppressed by miR-21, promoting cancer cell growth (Shen et al., 2020). TPM1, part of the actin-binding protein family, is crucial for regulating contraction by mediating calcium response in muscle. It acts as a tumor suppressor in neoplastic transformation, inhibiting cell growth and transformation (Sun et al., 2020). Research suggests TPM1 stabilizes the cytoskeleton, hindering actin filament movement, cell proliferation, and migration (Hardeman et al., 2020). Increased miR-21 under high glucose conditions downregulates TPM1, promoting cell proliferation and migration. Treatment with Tan counteracts this effect by upregulating TPM1, inhibiting proliferation and migration. Inhibiting TPM1 function partially reverses Tan’s anti-proliferative and anti-migrative effects (Jia et al., 2019).

MiR-21 represses the tumor suppressor PDCD4, impacting the mTOR pathway involved in cell growth and proliferation (Cristofoletti et al., 2019; Moustafa-Kamal et al., 2020). MiR-21 downregulates PDCD4 expression by directly targeting its 3′UTR region. PDCD4, a pro-inflammatory tumor suppressor protein, is consistently reduced in human cancers. Under normal conditions, PDCD4 is primarily in the nucleus but can move to the cytoplasm in response to environmental changes. PDCD4 regulates tumor cell apoptosis by binding to eukaryotic initiation factors 4A and 4E (Takaki and Eto, 2018; Wang and Yang, 2018; Matsuhashi et al., 2019). In breast cancer, miR-21 downregulates both PTEN and Smad7. Through PTEN inhibition, miR-21 induces fibroblast activation protein, promoting tumor cell growth. In contrast, Smad7 inhibition by miR-21 enhances fibroblast-to-myofibroblast transition and alpha-smooth muscle actin expression, boosting proliferation and invasion (Najjary et al., 2020). MiR-21 plays a role in the epithelial-mesenchymal transition of breast cancer cells by affecting the p-AKT and p-ERK pathways through PTEN inhibition (Najjary et al., 2020). The upregulation of Her2/neu receptors activates the MAPK signaling pathway, leading to increased miR-21 expression, promoting invasion and metastasis (Sareyeldin et al., 2019). Studies indicate that elevated miR-21 is associated with advanced breast cancer stages, metastasis, and poor prognosis (Anwar et al., 2019). MiR-21 influences invasion and metastasis through its targets PTEN, TPM1, PDCD4, and the tumor suppressor maspin (Huang et al., 2020), making it a potential target for cancer therapy. However, anti-cancer drugs like doxorubicin can paradoxically upregulate miR-21 through NF-kappa B activation, promoting breast cancer progression (O’Bryan et al., 2017). Nevertheless, targeting miR-21 in cancer therapy has shown increased sensitivity to anti-cancer agents in breast cancer (Hashemi et al., 2023).

## Dysregulated MiRNA profiles in breast cancer

### Breast cancer subtypes defined by gene and miRNA expression

**FIGURE 6:**
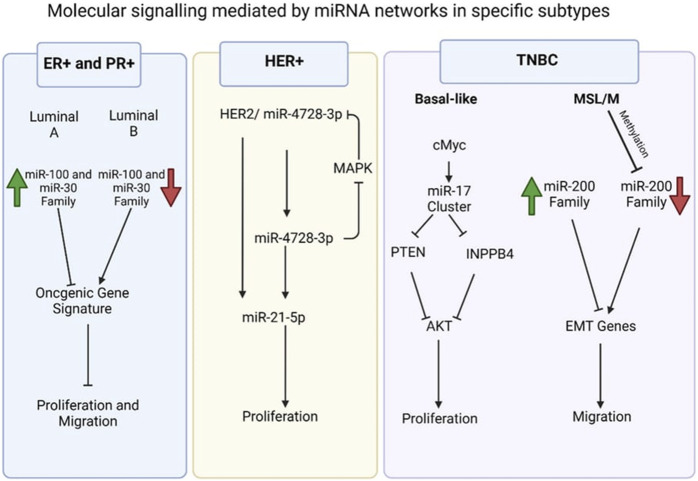
Molecular signalling mediated by miRNA network in specific subtypes (Arun et al., 2022).

Examples of significant miRNAs associated with specific breast cancer subtypes and their effects on cell phenotypes are shown in Figure 6; Table 1 demonstrates the list of miRNAs involvement in regulation of breast cancer. In estrogen receptor positive/progesterone receptor positive (ER+/PR+) breast cancers, significant miRNAs such as miR-100 and the miR-30 family play essential roles, particularly in distinguishing between luminal A and luminal B molecular subtypes. In human epidermal growth factor receptor two positive (HER2+) breast cancers, miR-4728-3p, located within the intronic region of HER2, is co-expressed with HER2 and involved in feedback regulation of HER2 and oncogenic miR-21-5p, thereby promoting several oncogenic processes in advanced tumors. In TNBCs, the cMYC oncogene-driven miR-17–92 cluster is overexpressed, especially in the basal-like one molecular subtype, promoting proliferation by targeting inhibitors of the proliferation mediator AKT such as PTEN and inositol polyphosphate-4-phosphatase type II B. Furthermore, in TNBC cell lines categorized as mesenchymal stem-like and mesenchymal molecular subtypes, epigenetic changes inhibit the miR-200 family, facilitating the expression of epithelial-to-mesenchymal transition and migration genes, ultimately promoting a migratory phenotype (Arun et al., 2022).

**TABLE 1:** Involvement of miRNAs in the regulation of breast cancer.

MicroRNA	Mechanisms of action	Involvement of Genes/Proteins	References
Major oncogenic microRNAs in breast cancer			
miR-1207-5p	Promotion of cell proliferation and G2 cell cycle progression	STAT2, CDKN1A, CDKN1B	Yan et al. (2017)
miR-492	Promotion of cell proliferation and G1–S cell cycle progression	SOX7, cyclin D1, c-MYC	Shen et al. (2015)
miR-135b	Promotion of cell proliferation and S–G2/M cell cycle progression	LATS2, CDK2, p-YAP	Hua et al. (2016)
miR-200c and miR-141	Promote metastasis and elevated in serum of metastatic mouse model and breast cancer patients	SerpinB2, c-Jun, c-Fos, FosB, FOXP3, KAT2B	Zhang et al. (2017b), Jin et al. (2017)
miR-331	Promotion of metastasis and invasion by elevation in plasma of metastatic breast cancer patients	HER2, HOTAIR, E2F1, DOHH, PHLPP	McAnena et al. (2019)
miR-200b	Promotion of metastasis and invasion	Ezrin/Radixin/Moesin (ERM)	Hong et al. (2016)
miR-122	Promotion of metastasis by reprogrammed glucose metabolism	pyruvate kinase (PK) and citrate synthase (CS)	Fong et al. (2015)
miR-374a	Promotion of metastasis by regulating EMT and Wnt/β-catenin signaling	E-cadherin, γ-catenin, CK18, vimentin, N-cadherin, Β-catenin, WIF1, PTEN, WNT5A	Cai et al. (2013)
miR-519a-3p	Promotion of apoptosis resistance and escape from natural killer cell recognition	RAIL-R2 (TNFRSF10B), caspase-8, caspase-7, MICA, ULBP2	Breunig et al. (2017)
miR-191-5p	Promotion of apoptosis resistance and doxorubicin resistance	SOX4, caspase-3, caspase-7, p53	Sharma et al. (2017)
miR-21	Pro-survival effect can be overcome by kallistatin	Akt, BCL-2, BAX	Li et al. (2016a)
miR-203	Pro-survival effect can be overcome by kallistatin	PKC-ERK, SOCS3	Li et al. (2016a)
miR-155	Telomere fragility and genomic instability	TRF1	Dinami et al. (2014)
miR-210	Hypoxia-inducible miRNA	HIFs, GPD1L, Pax-5	Camps et al. (2014), Costales et al. (2017), Zhang et al. (2017c)
miR-191	Hypoxia-inducible miRNA and stimulator of TGFβ-signaling pathways	HuR, TGFβ2, SMAD3, BMP4, JUN, FOS, PTGS2, CTGF, VEGFA	Nagpal et al. (2015)
miR-24	Hypoxia-inducible miRNA	Nanog, Oct-3/4, BimL, F1H1, HIF-1α, Snail, VEGFA	Roscigno et al. (2017)
Major tumor suppressor miRNAs in breast cancer			
miR-497	Anti-proliferative and G1-S cell cycle arrest	Cyclin E1	Luo et al. (2013)
	Anti-metastasis and anti-invasion	SMAD7	Liu et al. (2016)
	Anti-metastasis, anti-tumorigenic and inhibition of immune response or tumor immune escape	CD274	Yang et al. (2018b)
	Anti-angiogenesis and anti-tumorigenic	VEGF, HIF-1α	Wu et al. (2016)
miR-16	Anti-proliferative and G1–S cell cycle arrest, restores tamoxifen sensitivity	Cyclin E1, E2F7	Chu et al. (2015), Guo et al. (2015)
miR-30c-2-3p	Anti-proliferative and G1–S cell cycle arrest	Cyclin E1	Shukla et al. (2015)
miR-483-3p	Anti-proliferative and G1–S cell cycle arrest	Cyclin E1, p-NPAT, CDK2	Huang and Lyu (2018)
miR-143	Anti-proliferative	ERK5, MAP3K7, Cyclin D1	Zhou et al. (2017a)
miR-566	Anti-proliferative	CDK14, Cyclin D1, p21	Wang et al. (2017a)
miR-424	Anti-proliferative and G2–M cell cycle arrest	CDK1, YAP, p-ERK1/2	Xie et al. (2018)
miR-543	Anti-proliferative, cell cycle arrest and apoptosis	ERK/MAPK	Chen et al. (2017a)
miR-26a	Anti-proliferative, G1 cell cycle arrest and restores sensitivity to tamoxifen and trastuzumab treatment	Cyclin D1, CDK4, CDK6, p21, p27, p53, RNF6/ERα/BCL-xL, E2F7, MYC, cyclin E2	Huang et al. (2019)
miR-206	Anti-proliferative, cell cycle arrest and restores sensitivity to tamoxifen treatment	WBP2, p21, CDK4, cyclin D1	Ren et al. (2017)
miR-15a	Anti-proliferative and G1–S cell cycle arrest, restores tamoxifen sensitivity	Cyclin E1, E2F7	Chu et al. (2015)
miR-30b	Anti-proliferative, G1 cell cycle arrest and restores sensitivity to trastuzumab treatment	Cyclin E2	Tormo et al. (2017)
miR-365	Anti-proliferative and restores sensitivity to Fluorouracil chemotherapeutic treatment	GALNT4	Zhang et al. (2016)
miR-22	Anti-proliferative and restores sensitivity to Paclitaxel chemotherapeutic treatment	KRAS	Song et al. (2018)
miR-708	Anti-proliferative and regulates cell cycle arrest upon induction of glucocorticoid agonists, DEX and ATA	IKKβ, COX-2, c-MYC	Senthil Kumar et al. (2019)
miR-124a and miR-26b	Anti-metastasis and anti-invasion	SerpinB2	Jin et al. (2017)
miR-195	Anti-metastasis and anti-invasion by underregulation in plasma of metastatic breast cancer patients	FASN, HMGCR, ACACA, CYP27B1	McAnena et al. (2019)
	Anti-metastasis, anti-tumorigenic and inhibits immune response or tumor immune escape	CD274	Yang et al. (2018b)
miR-148a	Anti-metastasis and anti-invasion by regulating Wnt/β-catenin signaling pathway	WNT-1, β-catenin, MMP-7, TCF-4	Jiang et al. (2016)
	Promotes apoptotic response and overcomes chemoresistance	BCL-2, caspases	Li et al. (2017b)
miR-340	Anti-metastasis and anti-invasion by regulating Wnt/β-catenin and Rho/Rho-associated kinase (ROCK) signaling pathways	c-MYC, CTNNB1, ROCK1	Mohammadi-Yeganeh et al. (2016)
miR-34a	Anti-metastasis and anti-invasion by regulating EMT	TPD52, E-cadherin, TGF-β, N-cadherin	Li et al. (2016b)
	Pro-apoptotic effect can be induced by kallistatin	P53	Li et al. (2016a)
miR-138	Anti-metastasis and anti-invasion by regulating EMT	E-cadherin, vimentin, N-cadherin, Snail	Zhang et al. (2015a)
miR-494	Anti-metastasis and anti-invasion	PAK1, E-cadherin	Zhan et al. (2018)
miR-33b	Anti-metastasis and anti-invasion	HMGA2, SALL4, Twist 1	Lin et al. (2015)
miR-421	Anti-metastasis and anti-invasion	MTA1	Pan et al. (2016)
miR-193a	Anti-metastasis and anti-invasion	WT1	Xie et al. (2017)
miR-211-5p	Anti-metastasis and anti-invasion	SETBP1	Chen et al. (2017b)
miR-355	Anti-metastasis and anti-invasion	EphA4	Dong et al. (2018)
miR-133a	Anti-metastasis and anti-invasion	LASP1	Sui et al. (2022)
miR-124	Anti-metastasis and anti-invasion	STAT3	Shi et al. (2019)
miR-204-5p	Anti-metastasis, anti-tumorigenic, restores sensitivity towards PIK3CB inhibitors and chemotherapeutic drugs (i.e., doxorubicin, taxanes and bortezomib), and involved in tumor immune microenvironment remodeling	PIK3CB	Hong et al. (2019)
miR-204	Promotion of apoptotic response	JAK2, BCL-2, survivin	Wang et al. (2015)
miR-101	Promotion of apoptotic response by negatively regulating Notch pathway	EYA1, jagged1, Hes1, Hey1, SOX2	Wang et al. (2017b)
miR-296-5p and miR-512-5p	Reduction of telomerase activity, impairment of telomere maintenance and activation of replicative senescence and apoptosis programs	hTERT	Muraki et al. (2012)
miR-29b	Anti-angiogenesis and anti-tumorigenesis	Akt3, VEGF, c-MYC	Li et al. (2017c)
miR-140-5p	Anti-angiogenesis and anti-tumorigenesis	VEGFA, CD31, Ki-67, MMP-9	Lu et al. (2017)
miR-126	Anti-angiogenesis and anti-tumorigenesis	VEGFA	Alhasan (2019)
miR-100	Shuttling of miRNA enriched in MSC-derived exosomes, anti-angiogenesis and anti-tumorigenesis	VEGF, mTOR/HIF-1α	Pakravan et al. (2017)

### Specific MiRNAs linked to tumorigenesis, metastasis, and drug resistance

Several miRNAs have been identified as key players in tumorigenesis, metastasis, and drug resistance across various cancer types (Pan et al., 2021). These miRNAs play crucial roles in cancer progression and treatment response, making them potential targets for therapeutic interventions and biomarkers for prognosis and drug response prediction as shown in Table 2. However, their precise roles can vary depending on the specific cancer type and context, highlighting the complexity of miRNA-mediated regulation in cancer biology (Hill and Tran, 2021).

**TABLE 2:** MicroRNAs and their relationships with tumorigenesis, metastasis, and drug resistance.

MicroRNA	Tumorigenesis	Metastasis	Drug resistance	References
miR-21	Promotes tumorigenesis by targeting multiple tumor suppressor genes	Associated with metastasis by regulating various signalling pathways involved	Associated with drug resistance by regulating various signalling pathways involved	Najjary et al. (2020), Dan et al. (2021), Hashemi et al. (2023)
Known to be upregulated in many cancer types				
miR-155	Promotes tumorigenesis by targeting tumor suppressor genes	Promotes metastasis by targeting tumor suppressor genes	Implicated in drug resistance through modulation of cell survival pathways	Grimaldi et al. (2020)
Upregulated in several cancers				
miR-10b	Essential for tumorigenesis	Associated with promoting metastasis by regulating genes involved in cell motility and invasion	-	Raval et al. (2022)
miR-221/222	Promoting tumorigenesis by targeting various tumor suppressor genes and genes involved in cell cycle regulation and apoptosis	Promoting metastasis by targeting various tumor suppressor genes and genes involved in cell cycle regulation and apoptosis	Promoting drug resistance by targeting various tumor suppressor genes and genes involved in cell cycle regulation and apoptosis	Li et al. (2020a)
miR-34a	Downregulation of miR-34a is associated with tumorigenesis in various cancers	Downregulation of miR-34a is associated with metastasis in various cancers	Downregulation of miR-34a is associated with drug resistance in various cancers	Naghizadeh et al. (2020), Yang et al. (2021)
A tumor-suppressive miRNA.				
miR-200 family (including miR-200a, miR-200b, miR-200c, miR-141, and miR-429)	Key regulators of tumorigenesis and breast cancer development	Inhibit epithelial-to-mesenchymal transition (EMT), thereby suppressing metastasis. Downregulation of miR-200 family members is associated with metastasis many cancers	Downregulation of miR-200 family members is associated with drug resistance in many cancers	Tang et al. (2021), Jo et al. (2022)
miR-122	-	-	Regulate drug metabolism and sensitivity in hepatocellular carcinoma (HCC) and other cancers. Its downregulation is associated with drug resistance	Song et al. (2024)
Let-7 family	Frequently downregulated in cancers and are involved in regulating multiple oncogenic pathways, including tumorigenesis	Frequently downregulated in cancers and are involved in regulating multiple oncogenic pathways, including metastasis	Frequently downregulated in cancers and are involved in regulating multiple oncogenic pathways, including drug resistance	Ma et al. (2021), Karami et al. (2022b)

## MiRNA as diagnostic biomarkers

The care of patients in breast cancer remains a significant clinical problem. The scientific community is currently engaged in the ongoing exploration of supplementary biomarkers that can be employed in clinical settings to enhance the efficacy of oncologists in patient management (Jordan-Alejandre et al., 2023). Scientists have utilized genomics, bioinformatics, and molecular biology methodologies to identify miRNAs that offer significant diagnostic insights (Wu et al., 2014). As an illustration, Li et al. conducted a study wherein they employed the exiqon miRNA qPCR panel to screen plasma samples of breast cancer patients (as opposed to normal controls) and identified five distinct types of miRNAs (Let-7b-5p, miR-122-5p, miR-146b-5p, miR-210-3p, and miR-215-5p). Subsequently, they proceeded to validate their findings through quantitative reverse transcription polymerase chain reaction (qRT-PCR) and found that the ROC correlation coefficient for miRNA expression in both samples was 0.978. This finding provides evidence for the potential of the miRNA panel as a biomarker in the diagnosis of breast cancer (Li M. et al., 2019). In addition, Bakr et al. outlined the utilization of miR-373 as a diagnostic biomarker in individuals with breast cancer. Furthermore, they conducted an assessment of the target genes (VEGF and cyclin D1) of miR-373, revealing its role as an oncomiR. This finding suggests that miR-373 could serve as a crucial biomarker for the diagnosis and prognosis of breast cancer through its targeting of VEGF and cyclin D1 (Bakr et al., 2021).

In contrast, Zhang et al. conducted a study investigating the increase of miR-26b-5p, miR-106b-5p, miR-142-3p, miR-142-5p, miR-185-5p, and miR-362-5p in patients with breast cancer. The analysis demonstrated that the miRNA panel had the ability to distinguish between breast cancer patients and healthy controls, and it also exerted an influence on multiple cancer-related pathways (Zhang K. et al., 2021).

In addition, Lv et al. established a correlation between the decrease in miR-145 levels and the diagnosis of breast cancer, regardless of the specific kind of cells involved. A meta-analysis was conducted on a total of eight papers that employed quantitative methodologies utilizing qRT-PCR. They demonstrated that the expression of miR-145 was 2.57 times higher in breast cancer tissues compared to normal breast tissues. The research findings indicated a significant inverse correlation between the expression of miR-145 and the histological grade of tumors, so implying the potential utility of miR-145 as a biomarker (Lv et al., 2020). The diagnostic relevance of seven miRNA molecules (miR-126-5p, miR-144-5p, miR-144-3p, miR-301a-3p, miR-126-3p, miR-101-3p, and miR-664b-5p) was investigated and emphasized by Kahraman et al. The researchers employed RT-qPCR to examine 63 blood samples obtained from women, consisting of 21 patients diagnosed with TNBC and 21 healthy patients. The study revealed that miR-126-5p exhibited a substantial expression level, making it a prominent miRNA that suppresses tumor growth in breast cancer. Furthermore, it was found to be the most influential miRNA for diagnosing breast cancer in the panel under analysis (Kahraman et al., 2018). Table 3 presents a diverse range of miRNA compounds that have the potential to serve as biomarkers.

**TABLE 3:** MiRNA biomarkers as diagnosis and prognosis.

miRNA	Study type	Sample type	Sample source	Detection methodology	Potential as	References
Let-7b-5p, miR-122-5p, miR-146b-5p, miR-210-3p and miR-215-5p	Multi-phase validation	Blood samples	Hospital of Nanjing Medical University	Exiqon miRNA qPCR	Diagnosis	Li et al. (2019a)
miR-9 and miR-34a	Case–control	Tissues	National Tumor Bank and Isfahan Cancer Research Center of Seyed-o-Shohada Hospital	qRT-PCR	Diagnosis	Orangi and Motovali-Bashi (2019)
miR-185-5p and miR-362-5p	Case–control	Blood samples	Qilu Hospital of Shandong University	qRT-PCR	Diagnosis	Zhang et al. (2021a)
miR-126-5p, miR-144-5p, miR-144-3p, miR-301a-3p, miR-126-3p, miR-101-3p, and miR-664b-5p	Clinical trial	Blood samples	Medical Faculty of the Friedrich-Alexander University Erlangen-Nürnberg	qRT-PCR	Diagnosis	Kahraman et al. (2018)
miR-1246 and miR-21	*In vitro* and case–control	Exosomes from plasma	USA	Small RNA sequencing and qRT-PCR	Diagnosis	Hannafon et al. (2016)
miR-1246 and miR-21	Meta- Analysis	Peripheral blood samples	NR	ELISA and qRT-PCR	Diagnosis and prognosis	Wang et al. (2018a)
miR-21 and miR-27a	Case–control	Fasting venous blood	Jinan People’s Hospital Affiliated to Shandong First Medical University	qPCR	Diagnosis	Li et al. (2021b)

## MiRNA and cancer immune evasion

### MiRNA regulated innate immune response through immune cells

Immune cells in the body provide innate immune response whenever tumor cells arise which should kill and eliminate the tumor cells. However, these tumor cells are able to escape from the immune response and progress to another area of the body, aggravating the cancer (Thomopoulou et al., 2021).

This is made possible by the bi-directional communication between tumor cells and immune cells. These interactions create tumor microenvironment (TME) where tumor cells use different mechanisms to manipulate the cells around it (Tan and Naylor, 2022). Tumor cells manipulate other cells to meet their metabolic needs. Since tumor cell metabolism differs from normal cell metabolism, it competes with normal cells for resources like glucose. In an article by Elia and Haigis (2021), it is stated that T cells and cancer cells compete for glucose and amino acids. Due to T cell dependency on metabolism, limited glucose supply alters its function and differentiation which helps in tumor progression. Similarly, other immune cells also compete with cancer cells to sustain their metabolism which affects the normal function of the immune cells thus allowing the tumor to progress and metastasize (Elia and Haigis, 2021).

The impaired functionality of immune cells can either be corrected or further impaired by miRNAs. MiRNAs play a crucial role in cancer immune evasion by regulating the pathways of immune cells involved in cancer like macrophages, NK cells, Tregg cells, and T helper cells (Cortez et al., 2019). Unfortunately, while some miRNAs can manipulate the impaired immune cells to stop tumor progression, others influence tumor cells activities leading to cancer progression.

MiRNAs can be used as biomarkers for various cancers and infectious diseases. In breast cancer patients, miRNA-125b (miR-125b) and miRNA-155 (mir-155) are used for diagnosis, monitoring prognosis, and assessing patient’s response to chemotherapy. In addition to that, they are also believed to have the ability to regulate innate immune response in breast cancer by modulating the M1/M2 profile.

The most discussed miRNA in relation to breast cancer is miR-155. In breast cancer, overexpression of miR-155 was initially thought to be oncogenic however, a study conducted by Wang et al. (2022), proves otherwise when it demonstrates that miR-155 deficient macrophages increases tumor progression while macrophages showing an overexpression of these noncoding RNAs improves tumor suppression (Wang et al., 2022a). One of the main functions of miR-155 is regulating innate immune responses by differentiating B lymphocytes and CD4^+^ T cells as well as activating Tregg cells. Furthermore, miR-155 has a direct relation with dendritic cells in which during the immune response in the early stage of breast cancer, both activation of dendritic cells and expression of miR-155 are increased. A study by Asadirad et al. (2019), stated that miR-155 is transported into dendritic cells via the exosomes derived by the cancer cells to increase the expression of immune cells like MHCII, CD86, CD40, CD83, IL12p70, IFN- γ , and IL10. In addition to that, the abundance of miR-155 seen in early breast cancer patients as compared to metastatic breast cancer patients indicates that miR-155 is responsible for the regulation of innate immune response in breast cancer (Asadirad et al., 2019).

Other miRNAs that are highly expressed in early breast cancer include miRNA-126 (miR-126), miRNA-19a (miR-19a), and miRNA-20 (miR-20). Due to that, these three miRNAs are also believed to have an influence in the regulation of innate immune response in breast cancer. MiR-126 prevents breast cancer metastasis by reshaping the TME. Reshaping of TME is made possible by targeting the stromal cell-derived factor 1 (SDF-1) which suppresses the production of monocytes. MiR-19a prevents breast cancer metastasis by converting tumor-associated macrophages (TAM) or M2 macrophages to M1 macrophages through the inhibition of various proto-oncogenes. MiR-20 is a T-helper cells modulator which affects breast cancer by targeting FOXO-1 and STAT3 pathway (Ilhan et al., 2023).

**FIGURE 7:**
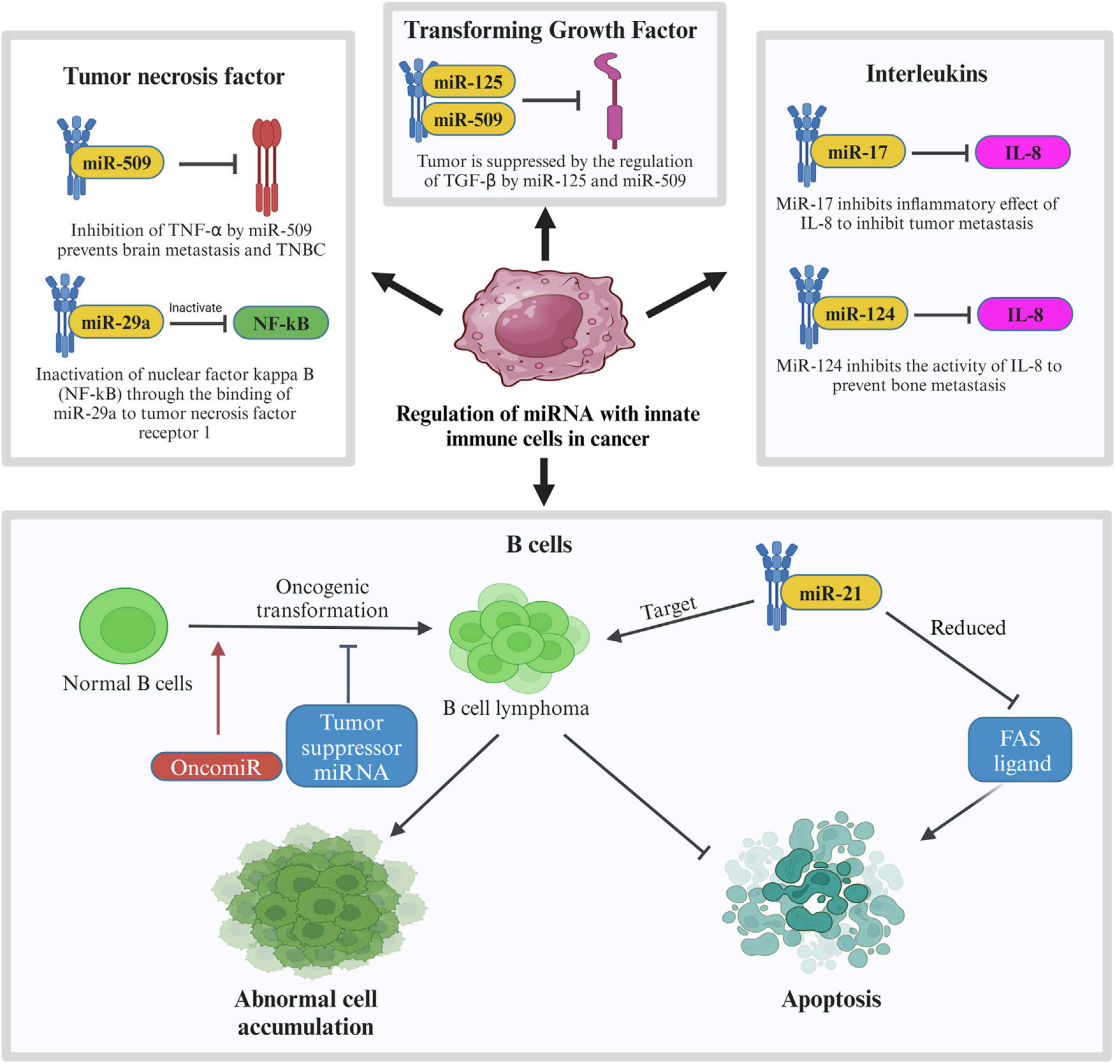
Regulation of miRNA with innate immune cells in cancer. In breast cancer, innate immune cells including tumor necrosis factor, transforming growth factor, interleukins and B cells are involved in regulation of miRNA in which their pathways are interrupted by tumor suppressor miRNAs to prevent further tumor metastasis. In contrast, oncogenic MiR-21 is involved in the mechanism by targeting B cell lymphoma, leading to abnormal cell accumulation and suppression of apoptosis.

Like miR-20, many other miRNAs affect the pathway of cytokines produced by the innate immune system as seen in Figure 7. When the body detects breast cancer cells, innate immune cells like lymphocytes and macrophages secrete cytokines like tumor necrosis factor (TNF), transforming growth factor (TGF), and interleukins (IL). MiRNAs affect these cytokines to either promote or suppress tumor progression (Bahiraee et al., 2019). One of the cytokines, tumor necrosis factor-alpha (TNF-α), is involved in brain metastasis of primary breast cancer. MiRNA-509 (miR-509) prevents this action by suppressing the levels of TNF-α regulated in the tumor environment. Suppression of TNF-α by miR-509 also prevents TNBC cell proliferation and invasion. Another miRNA involved with TNF is miRNA-29a (miR-29a). This miRNA binds to 3′-UTR of tumor necrosis factor receptor 1 (TNFR1) gene to inactivate the nuclear factor kappa B (NFκB) pathway. Inactivation of this pathway reduces the cell proliferation and induces apoptosis to MCF-7 cell which is a cell from the breast cancer cell line (Zhao et al., 2017).

Another type of cytokine, transforming growth factor beta (TGF-β) plays a very important role in cell differentiation, proliferation, and motility in breast cancer which also makes it crucial for TGF-β to be controlled by a regulatory network (Tzavlaki and Moustakas, 2020). In breast cancer, the regulation of TGF-β is suppressed which leads to its overactivation. Overactivation of TGF-β promotes invasion and metastasis of breast cancer cells. In relation to TGF-β in breast cancer, some miRNAs like miR-10b, miR-21, miR-106b, and miR-181 are regulated by TGF-β to promote metastasis while others like miR-145 and miR-206 regulate TGF-β to suppress tumor progression. Other than TGFs, innate immune cells also produce various interleukins. IL-1, IL-8, and IL-11 are known to aid in tumor progression in breast cancer. IN TME, these ILs promote invasion and metastasis of the cancer cells. Certain miRNAs like miR-17, miR-20, and miR-124 supress the effect of these ILs to inhibit tumor progression. MiR-17 and miR-20 bind to 3′-UTR of IL-8 to inhibit the in the inhibition of tumor metastasis to the neighbouring cells whereas the expression of miR-124 prevents bone metastasis caused by IL-11. It is observed that miR-124 has a direct downstream effect to IL-11 by reducing the luciferase activity of the 3′-UTR of IL-11. Overexpression of miR-124 inhibits the activity of IL-11 which prevents bone metastasis in breast cancer patients.

Another interleukin targeted by miRNAs in breast cancer is IL-6. Targeting this interleukin has a dual effect in breast cancer which may either be tumorigenic or anti-tumorigenic. The tumorigenic effect is caused by inhibiting apoptosis, triggering the survival of the tumor cells, and allowing metastasis. IL-6 stimulates miR-155 expression which is known to target suppressors of cytokine signalling pathway (SOCS), hence promoting the progression of breast cancer. Blocking IL-6 pathway can prevent this progression through the inhibition of tumor migration and invasion (Gyamfi et al., 2018). Another method of overcoming this situation is through the downregulation of miR-155 using a photosensitizer. The anti-tumorigenic effects are caused by inhibiting IL-6/STAT3 and SOCS pathway. One of the characteristics of miR-146 is suppression of inflammatory cytokines expression (Gao and Dong, 2017) which makes it a good tumor suppressor marker. In primary breast cancer, miR-146 expresses a negative feedback loop to IL-6 as a mean to inhibit the tumor progression initiated by IL-6/STAT3 pathway. Other miRNAs that can block IL-6 pathway are miR-203 and miR-7. MiR-203 directly inhibits the SOCS pathway in MCF7 cells which suppresses breast cancer progression while miR-7 directly binds to 3′-UTR of IL-6 to negatively regulate its expression which results in antitumorigenic and antimetastatic effects in breast cancer. Figure 7 shows the regulation of miRNA with innate immune cells in breast cancer.

Although the immune system has similar mechanism in every individual, in breast cancer patients, the expression of miRNAs may differ in different patients based on their race. This is because they are genetically influenced and causing women of different races to have different miRNAs expression. A study on Latina breast cancer patients shows that upregulation of miR-141-3-p is associated with cancer recurrence, while downregulation of miR-166 and miR-150-5p are associated with large tumors (Almohaywi et al., 2023). Another study conducted on Egyptian breast cancer patients shows an overexpression of miR-29b and under expression of miR-31 that are linked to the patient’s relapse (Abbas et al., 2022). Therefore, patient ancestry is an important factor when considering the breast cancer biomarkers.

### MiRNA regulates specific immune responses in breast cancer

The specific immune system is also known as adaptive immune response which produces respective antibodies that can be used to fight against the particular microbes. The certain microbes that have previously entered the body will be immediately attacked by the immune system if they again infect the specific individual (NCHBI, 2020). The common examples of specific immune responses in breast cancers are the cytotoxic T lymphocytes, natural killer cells and B cells. The cytotoxic T lymphocytes and natural killer cells exert their effect in a direct route onto the breast cancer cells by killing the abnormal cells therefore to induce the antitumor immunity. They play the dominant character in fighting against breast cancer. As breast cancer is growing, the quantity of T-cells will be increasing simultaneously but often associated with reduction in its function due to the extremely immuno-suppressive surroundings in malignant conditions (Speiser and Verdeil, 2017). On the other hand, B cells will result in antitumor immunity through the secretion of immunoglobulins which are able to minimize the amount of early neoplasms (Amens et al., 2021b).

In this case, the tumor cells will have two-way interaction with their local environment which known as tumor microenvironment with the presence of tumor, normal breast, immune, endothelial cells, fibroblast that found in the existence of cancer and mesenchymal stromal (Syeda et al., 2022). By simply secreting the small extracellular vesicles, bidirectionally, tumor cells able to signal the other components. These generated small extracellular vesicles may modulate the activity of mesenchymal stromal, cancer-associated fibroblasts and immune. Meanwhile particularly during hypoxic condition, the performance of endothelial cells can be controlled by mesenchymal stromal and tumor (Rashid et al., 2023). The combination of immune, cancer-associated fibroblast and endothelial cells may enhance angiogenesis (Fu et al., 2020). The major role will be presented by the breast cells which able to largely control the antitumor immune response by producing the small extracellular vesicles and certain extracellular signaling molecules (Mammes et al., 2021). It therefore led to destruction in the immune cells as the products consist of numerous T cells that responsible to impede or activate the polarization, activation, expansion, movement and recruitment of the target cells (Loric et al., 2023).

**FIGURE 8:**
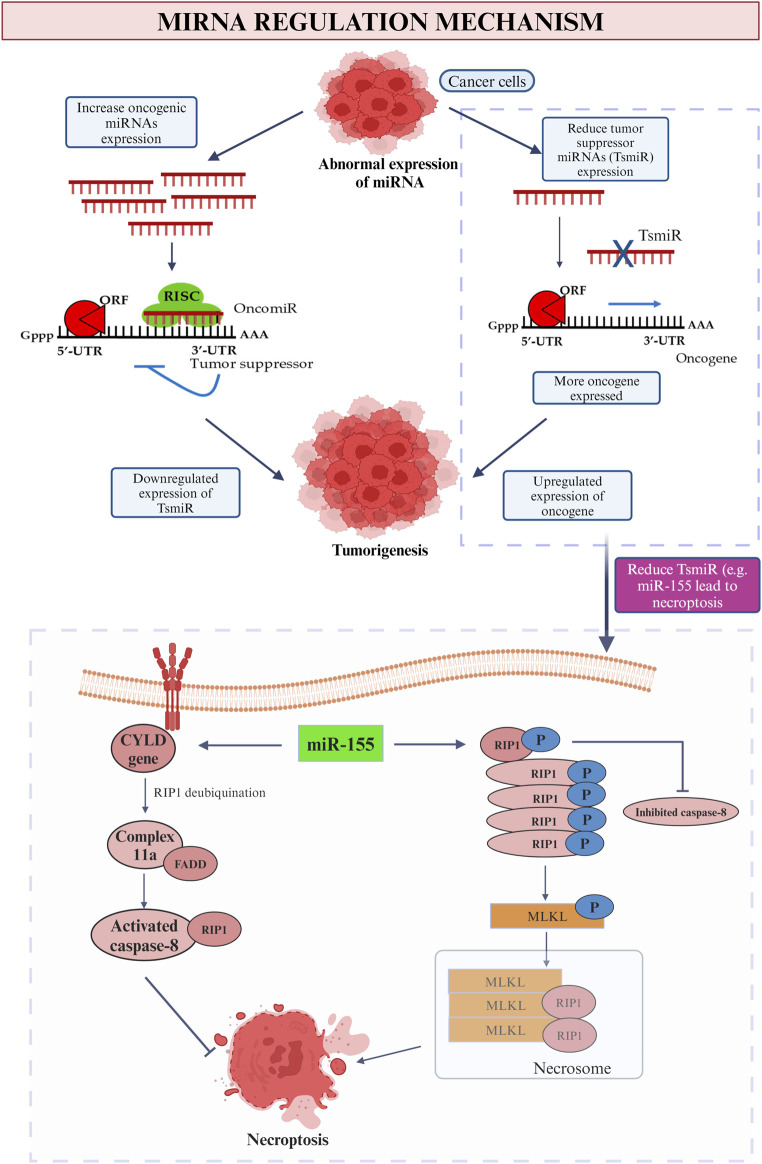
MiRNA regulation mechanism. This picture depicted the role of oncogenic and tumor suppressor miRNA in cancer and the influence on tumor suppressor miRNA (miR-155) on promotion and inhibition of necroptosis.

There are numerous miRNAs have been studied previously and it was found that they are capable in both enhancing and impeding tumor development, depends on the types and roles of each miRNA. Many of them are proven to be related to invasion, prognosis and the formation of new blood vessels (Hussen et al., 2021). The miRNA that is involved in breast cancer can be categorized into few classes such as the oncogenic miRNA and tumor suppressor miRNA (Figure 8). Subdivision can be continued from both classes into various families. These miRNAs are derived from both the tumor and stroma cells to perform their role in inhibiting the immune system (Wang et al., 2022b). Positive regulation of oncogenic miRNAs hinder the tumor suppressor gene thus allow the formation of tumor while in contra to it, negative modulation of miRNAs with tumor suppressor effect will improve the oncogenes translation (Zacharias et al., 2020). As an example of tumor suppressor miRNA in suppressing the cell proliferation, miR-340 is one of the best instance by which it obstructs the Wnt appearance by aiming LGR5 and FHL2 thus inhibit the cell proliferation (Otmani and Lewalle, 2021).

In the context of promoting malignancy, oncogene miRNA is responsible to inhibit the function of the specific tumor suppressor genes thus boosting the cancer progression and promote breast malignancy. Meanwhile, tumor suppressor miRNA restrains the oncogenes that related in breast tumorigenesis thus downregulate and leads to breast cancer (Saikia et al., 2020). In a nutshell, irregular miRNAs will be found in the appearance of cancer cell. Increase level of oncogenic mRNA results in lesser manifesting of tumor suppressor gene while due to the low level of tumor suppressor miRNAs, positive feedback possessed onto the appearance of oncogene. Both of the feedbacks lead to tumorigenesis which involves the process of cell proliferation, invasion, prevent apoptotic event, angiogenesis, replicative immortality and metastasis (Loh et al., 2019). On the contrary, with the purpose to inhibit the breast cancer development, certain miRNAs may be participated in the replacement therapies which hamper the appearance of oncogenes (Smolarz et al., 2022). To be more persuading, a research held in 2014 found that patients who diagnosed with early stage breast cancer will have increased level of miR-155, miR-19a, miR-181b and miR-24 as compared to normal healthy people (Hamam et al., 2017).

As an example of oncogenic miRNA, high level of miR-155 play an antitumor role in breast cancer through the directly suppressing of cytokine signaling (SOCS1) which aids in the modulation of IFN-y production in T cells is vital to assemble and activate the adequate amount of T effector cells (Chen et al., 2020). In breast cancer, it is the dominant regulator in metabolizing glucose hence able to promote the energy metabolism through few pathways. Moreover, the coordination between NF-κB-miR-155 axis and NF-κB-miR-146a axis may also upregulating the inflammatory reaction (Hoorzad et al., 2023). The above founding all indicate high level of miR-155 is potent as a role that inhibiting the activity of immune response. However, it is also crucial in the first line differentiation of myeloid progenitors, responsible for various cellular processes such like proliferation, stemness, apoptosis and angiogenesis, dysregulation of it may still result in hematologic problem and exerting its oncogenic natural in solid tumors such as breast cancer (Kalkusova et al., 2022).

On the other hand, tumor suppressor miRNAs such as miR-23a/27a/24-2, miR-146a and miR-223a also possessed similar responsibilities but in a different alternative. As an instance, miR-146a is the main component that responsible to downregulate the differentiation of Th1 cell (Testa et al., 2017). Along with the elevating level of IFN-y in miR-146a, destruction is able to be seen on both CD4^+^ and CD8^+^ T cells (Mastroianni et al., 2019). When the signal transducer and activator of transcription one is not able to be targeted, the Treg cells will no longer capable of impeding the Th1 response which triggered by IFN (Goropevšek et al., 2017). Assemble of innate immune cells, autoantibodies as well as triggered CD4^+^ and CD8^+^ T cells were found to result in autoimmune disorders and severe hyperinflammatory (Li B. et al., 2017). Moreover, with the stimulation of T-cells, it negatively responses in limiting the NF-kB activity through the suppression of NF-kB activators (Wang et al., 2019). This communication is proved to reduce the death in T-cells that enhanced by activation.

Furthermore, elevate expression of miR-494 in myeloid-derived suppressor cells (MDSCs) diminish the quantity of PTEN thus result in the accumulation of MDSCs and help in the invasion and metastasis of breast tumor. In normal situation, PTEN represents the major pathway to regulate the activation and reproduction of several immune cells including T- and B- cells (Taylor et al., 2019). Hence, it is reduced in this case via the lipid phosphatase and protein phosphatase activities to diminish its ability in producing divergent tumor suppressing genes (Chen et al., 2022). When PTEN is not enough to construct the gene that suppresses the tumor growth, then a high amount of MDSCs will be collected in the respective area. MDSCs have potential to impede the T cells activities thus create a favorable condition for tumor to develop and proliferate without any interruption. Meanwhile, it also improves the tumor with resistance to immunotherapy therefore its survival is even assured (Law et al., 2020).

Meanwhile, miR-34a is a well-known miRNA that can be simply detected in every healthy cell with the responsibility to repress various tumors which comprise the breast, colon, prostate cancer and more. According to Rui et al. (2018), they suggested that the elevated the number of miR-34a, the more the breast cancer is under controlled (Rui et al., 2018). A series of assessments have been performed in order to support their statement which include the use of TargetScan device to expose the effect of miR-34a towards Notch1. In a more particular way, Notch1 is an elementary vital option that contribute in the emergence of breast tumor as it consists of Delta-like-4 that lead to the formation of new useless blood vessels in the malignancy conditions when the Delta-like-4 is being downregulated (Kontomanolis et al., 2018). It is crucial to modulate the proliferation of breast tumor. Besides, MTT examination states that the survival rate of the breast cancer cell line, MCF-7 cell is apparently reduced by miR-34a. It prompts the abnormal cells that can be found in breast to perform apoptosis and some of the malignant cells are arrested in G1 phase to prevent the further atypical replication of mutated DNA (Ngabire et al., 2018). With this, it is strong evidence showing that increasing amount of miR-34a is highly beneficial in treating and inhibiting breast cancer. It may be used as a therapeutic agent to cure the breast cancer.

Moreover, let-7 family that can be counted as a member of miRNA which is found ahead of the other miRNAs play an important role to suppress numerous cancer development. The name lethal-7 is given as its evolution often result in death of the patient (Bernstein et al., 2021). It is made up of 22 nucleotides but does not possess the ability to encode a protein. Meantime, it is a big family which act as an originate for nine miRNAs which are the let-7a, let-7b, let-7c, let-7d, let-7e, let-7f, let-7g, let-7i and miR-98 (Mizuno et al., 2018). Out of various functions that can be performed by this big family, they also capable of restricting the growth of breast cancer via impeding the proliferation, metastases and invasion of the abnormal cells by targeting MAGE-A (Liu et al., 2019). MAGE-A is also known as Melanoma-associated antigen-A which is an antigen that is noticed with the presence of tumor. These antigens will be noted by the cytotoxic T cells thus lead to the activation of a strong specific immune response that fights against the breast tumor. Elevated appearance of MAGE-A genes always indicating that the patient may have a reduced survival rate from breast cancer (Poojary et al., 2020). Based on the declaration by (121), miRNA let-7a also able to decrease the presence of E2F2 protein in order to inhibit the aberrant reproduction of osteosarcoma cells, which is the proliferation of high-grade malignant mesenchymal cancer cell that derived from the abnormal bone and osteoid (Dekkers et al., 2019). The E2F transcription factor 2 (E2F2) that stimulated by the infected E2F or Myc proteins is vital for tumorigenesis by which high expression of E2F2 may assist in high grade tumor, metastasis of cancer cell or any other terrible prognosis (Świętek et al., 2023).

### MiRNA regulated cancer mediated immune cell death

Immune cell death may occur in two approaches which include the death of cell, apoptosis and destruction of the body tissues, necrosis when there is only inadequate blood flows to the particular area (Khalid and Azimpouran, 2023). Apoptosis is commonly a pathway that rigorously performed by every cells each time when the cells reach to the limit of their lifespan but the condition become out of control in the presence of cancer cells (Shirjang et al., 2019). As explained by Taghavipour et al. (2020), miRNAs able to enhance and reduce the chance of apoptosis through two different alternatives which are the intrinsic and extrinsic route (Taghavipour et al., 2020). Intrinsic pathway will be stimulated when there is destruction of DNA while apoptosis through extrinsic pathway occurs indirectly when the cells are alerted by the other cells that ordered them to perform cell death (Jang and Lee, 2021). Figure 8 demonstrates the summarization story of how apoptosis is being upregulated and downregulated by miRNA under different conditions with miR-21, the common miRNA that is involved in breast cancer as an instance.

In the case of extrinsic pathway, the appearance of fas ligand (fasL) is proved to be directly reduced by the miR-21 in the breast cancer cell line to promote the apoptotic event (Abtin et al., 2018). Meantime, miR-21 may target certain anti-apoptotic proteins in some particular cases thus reduce the ability of cancer cells to migrate and perform programme cell death. To provide a more understandable illustration, there are abundant genes that responsible to diminish the capability of abnormal cells to migrate from one place to another will be targeted by miR-21 such as the PTEN, tropomyosin 1 (TPM1), metalloproteinase inhibitor 3 (TIMP3), programmed cell death protein 4 (PDCD4) and so on. Similar to the episode that suppress the apoptotic event, PDCD4, a kind of anti-apoptotic protein, also targeted in this case with the addition of directly aiming on the B cell lymphoma 2 (Bautista-Sánchez et al., 2020a). As an extra knowledge, B cell lymphoma two possess of the ability to inhibit the apoptosis activity and assemble the abnormal cells in the same time. Thus, apoptosis can be reduced by the miR-21 that target on B cell lymphoma 2 (Bashir et al., 2021). Moreover, miR-7-5p also an instance of it by which it is responsible to impede the proliferation of cancer cells thus to enhance the abnormal cells to undergo programme cell death by virtue of aiming the REGy, proteasome activator subunit 3 (Dong et al., 2019). Conversely, for intrinsic pathway, the relationship between miR-15a, miR-16 and the transcription of 3′UTR in BMI1 has been proved to be beneficial in indirectly promoting the intrinsic route of apoptosis. In details, these miRNAs inhibit the presence of BMI1 during the transcription period. The protein BCL2 that in charge of depleting the apoptotic event will be reduced meanwhile increase the proteins that initiate the apoptosis. It then triggers a series of activities including the rising of mitochondrial reactive oxygen species (ROS) extent that lead to the disability of potential in mitochondrial membrane thus allow the cytochrome c to enter cytosol then initialize the Caspase-3 and Caspase-6 or -9 (Ghafouri-Fard et al., 2021).

The succeeding immune cell death is the occasion of necroptosis. As a general, caspase activities are blocked by which the cell death will be accomplished solitarily without the existence of caspase events in the stimulation of tumor necrosis factor (TNF) family (Jang and Lee, 2021). As the most common miRNA for breast cancer, miR-155 play a role in both up- and downregulation of necroptosis (Figure 8). First of all, by straightly targeting the Receptor-Interacting Protein 1 (RIP1) in human cardiomyocyte progenitor cells, about 40% of necroptosis is able to be inhibited (Ro et al., 2018). As soon as the TNF family initiate the necroptosis process, RIP1 will be deubiquitinated by CYLD gene thus trigger the formation of complex IIa that composed of caspase 8, RIP1 and FADD. The activated caspase-8 divides RIP1 therefor achieves the purpose to impede necroptosis and prolong the cell lifespan (Gong et al., 2019). The upregulation of cell necroptosis also can be done by miR-155 by targeting the same protein kinase, RIP1. The elevated quantity of RIP1 that merged with RIP3 along the suppression of caspase-8 will result in the emergence of a compound named as necrosome (Li et al., 2023). Then the substrate of RIP3 kinase, mixed-lineage kinase domain-like protein (MLKL) initializes the phosphorylation procedure and ready itself for necroptosis by translocating to the plasma membrane. The greatly improved in the permeability of plasma membrane and the disruption of integrity then induce the occurrence of necrosis (Zhirong et al., 2021).

### Therapeutic strategies

Despite tremendous breakthroughs in treatment options for breast cancer, there is still an urgent need for innovative therapeutic strategies to enhance patient outcomes (Grimaldi et al., 2021). MiRNAs have recently attracted attention as potential therapeutic targets in cancer treatment. Changes in miRNA expression have been linked to a variety of disorders, including cancer, and therapy strategies based on miRNAs have showed promise in both preclinical and clinical studies (Forterre et al., 2020).

#### MiRNA-based therapies

Previously, research on miRNAs focused on characterising them, developing miRNA mimics and inhibitors, combining miRNA-based treatments with other medical interventions, and developing nanoparticle-based delivery techniques. Particular strategies in the context of breast cancer include the finding of miRNA patterns for diagnosis and prognosis, targeting of cancer-promoting miRNAs, and the development of miRNA-based treatments such as miRNA inhibitors. These techniques aim to decrease the probability of developing the disease. Several miRNAs, including miR-23a/b, miR-27a/b, miR-133, and miR-24a, are notable for their ability to impact cell fate decisions by regulating key proteins involved in apoptosis. Targeting these miRNAs is a promising technique to influence the susceptibility of cancer cells to anti-cancer therapy. Proteins involved in both intrinsic and extrinsic apoptotic pathways are described in the text, and they might be therapeutic targets for the treatment of cancer. The importance of miRNAs in controlling cell death pathways and their promise as cancer treatment targets, underscores the necessity for more study into these programmed cell death processes.

An aggressive form of breast cancer, TNBC accounts for 15%–20% of all breast malignancies. Its limited therapy choices are caused by the absence of certain receptors. Residual disease is frequently left behind by current neoadjuvant treatment (Qattan, 2020). By controlling gene expression, RNA-mediated interference is emerging as a potential therapeutic. But problems still exist since RNA-mediated interference is not very stable and breaks down quickly in the blood. One potential answer is the pRNA-3WJ scaffold, which is a product of RNA nanotechnology. By providing a robust foundation, this scaffold enables the construction of multifunctional RNA nanoparticles that carry anti-miR21 (Lin et al., 2020). The binding affinity to oncogenic miR21 is enhanced by modifications such as locked nucleic acid (LNA). Targeting CD133 with an RNA aptamer enhances targeting specificity since CD133 is expressed in TNBC and may function as a particular targeting receptor. *In vitro* and *in vivo* studies have assessed the targeting affinity, therapeutic effectiveness, and gene regulation consequences of the engineered RNA nanoparticles, which have subsequently been used to treat TNBC. The goal of this novel strategy is to improve therapeutic delivery and make tailored therapy for TNBC possible (Yin et al., 2019).

MiRNAs play a crucial role as clinical biomarkers in cancer, exerting an impact on prognosis, diagnosis, and treatment responses. These entities demonstrate substantial influence as oncogenes and tumor suppressors, which affects conventional treatments and immune reactions within the tumor microenvironment (TME) (Seliger et al., 2014). The utilisation of therapeutics that target miRNAs exhibits potential for enhancing immunotherapy in combination with established treatments. Preclinical investigations classify miRNA-based approaches into two distinct categories: antagonists and mimics. Mimics reinstate miRNAs that inhibit tumor growth, thereby increasing the tumor’s susceptibility to standard therapies and fortifying its immunogenicity. Clinical trials have investigated miR-34a mimics, which have demonstrated potential in modulating immune cell infiltration into tumor tissue and augmenting anti-tumor immune responses (XRT-mediated). Nevertheless, toxicity concerns prompted the phase I trial to be terminated early. Additional potential targets of miRNA mimics, such as miR-124 and miR-424, have exhibited the capacity to interfere with immune checkpoint signalling, thereby enhancing survival rates in glioma and ovarian carcinoma models, respectively. In a glioma model, administration of miR-138 substantially reduces the expression of CTLA-4, PD-1, and Foxp3 on CD4^+^ T cells that infiltrate the tumor. Clinical trials of miRNA inhibitors that target oncogenic miRNAs have commenced in the interim. As an anti-miR-155 agent, MRG105 inhibited the growth and metastasis of tumors. Nonetheless, systemic administration may impair the function of immune cells. By inhibiting effector functions of immune cells, such as miR-23a, antitumor immune responses are strengthened. Additionally, miRNAs improve the functionality of chimeric antigen receptor (CAR) T cells, which is crucial for adoptive cell therapy. The cytotoxicity and efficacy of CAR T cells are enhanced through the combination with specific miRNAs, as demonstrated by their ability to target EGFRvIII for glioblastoma or HER2 for cancer therapy (Tang et al., 2022).

The potential therapeutic application of miRNAs in cancer immunotherapy is vast, evident from their crucial role in regulating immune evasion within the TME. These miRNAs influence essential immune responses by selectively targeting various cellular components, such as dendritic cells (DCs), regulatory T-cells (Tregs), natural killer cells (NK), and macrophages. For instance, neutralizing miR-146a-5p alters the TME, disrupting macrophage-tumor cell communication and potentially bolstering anti-tumor immunity. Moreover, miRNAs like miR-23a-3p, miR-27a-3p, miR-155, miR-142-5p, miR-183, and miR-214 could serve as therapeutic targets due to their association with immunosuppressive mechanisms, indicating the potential to enhance immune responses against tumors and the significance of miRNAs in cancer immunotherapy (Raue et al., 2021). MiR-155 stands out for its pivotal role in stimulating anti-tumor immunity in T cells, enhancing functionality, memory formation, cytotoxicity against tumor cells, and promoting IFN-γ production. Its overexpression suppresses SOCS1 in CD8^+^ T cells, amplifying antitumor immunity, and cultivates a proinflammatory milieu within the tumor by promoting IFN-γ generation by Th1 cells. While modulating miRNA expression augments T-cell activity against specific tumor antigens, IFN-γ signaling upregulates PD-L1, fostering a pro-tumor milieu. Combining immune checkpoint blockade inhibitors with miRNA-targeting therapeutics could optimize the antitumor effect (Cortez et al., 2019).

#### MiRNA-based therapies—mimics

This intervention is to restore lost miRNA expression and it holds potential in revolutionizing cancer treatment due to their correlation with tumorigenesis. The potential improvement in the efficacy of cancer treatments lies in targeting multiple immune checkpoints by combining miRNA therapy with immune checkpoint blockade. While the combination method of immune checkpoint blockade and microRNAs has not yet advanced to clinical trials, there is increasing preclinical evidence of possible synergistic interactions between the two. Preclinical evidence suggests synergistic interactions between immune checkpoint blockade and miRNAs, particularly miR-424 and miR-138, which target PD-L1, CD80, PD-1, and CTLA-4, presenting capacity for treating malignancy. Overcoming the complex interplay between miRNAs and immune checkpoints is essential to exploit the potential of miRNA-ICI synergy in breast cancer treatment (Zhou et al., 2023a).

#### Selected MicroRNA targeted multiple immune checkpoints in breast cancer

Selected miRNAs that have the capacity to influence multiple immunological checkpoints in breast cancer cells have been chosen for potential application in immunotherapy, based on the current evidence.

MiR-149-3p: MiR-149-3p, a gene that suppresses cancer, has been found to reduce cell proliferation, migration, and invasion, and induce apoptosis in various types of cancer cells, including breast cancer (Li et al., 2022). Furthermore, there is data indicating that miR-149-3p is capable of controlling the expression of PD-1, TIM-3, and BTLA by attaching to the 3′UTRs of their mRNAs. This action helps to inhibit tumor immune escape (Zhang et al., 2019). Furthermore, the upregulated expression of miR-149-3p has been seen to reduce the programmed cell death of T cells and attenuate the miRNA indicators of T cell fatigue, resulting in enhanced cytotoxicity of CD8^+^ T cells against 4T1 breast cancer cells (Zhang et al., 2019). Therefore, miR-149-3p could potentially be utilized in breast cancer immunotherapy by controlling the expression of PD-1, TIM-3, and BTLA.

MiR-195/MiR-497: Prior research has indicated that the levels of miR-195/miR-497 vary between normal and cancerous breast tissues, suggesting that miR-195/miR-497 could serve as a promising biomarker for the treatment of breast cancer (Li X. et al., 2019; Purohit et al., 2019). Additional bioinformatic study revealed that miR-195/miR-497 have the capacity to control the immune evasion of breast cancer cells by specifically targeting PD-L1 and B7-H6 (Yang et al., 2018a). For example, the process of introducing miR-195/miR-497 into MDA-MB-231 cells has been discovered to significantly reduce the levels of PD-L1 messenger RNA (mRNA) and protein expression (Yang et al., 2018a). Furthermore, the introduction of miR-195 has been shown to decrease the levels of PD-L1 mRNA and protein expression. This leads to a decrease in the growth and movement of OCI-Ly10 cells, which are a type of diffuse large B cell lymphoma. Additionally, it prevents these cells from evading the immune system and increases the rate of cell death through apoptosis [28]. Furthermore, the analysis of transcriptome profiling data from the TCGA database (Yang et al., 2018a) has shown that miR-195/miR-497 can target PD-L1 and B7-H6. Therefore, the miR-195/miR-497 combination has been suggested as a promising option for using immune checkpoint blockade therapy in breast cancer. This combination works by controlling the levels of PD-L1 and B7-H6 expression.

MiR-5119: According to a study, MiR-5119 has been identified as a regulator of anti-tumor immunity in breast cancer (Zhang M. et al., 2020). A recent study has discovered that by engineering dendritic cells (DCs) and utilizing miR-5119, a new therapeutic approach can be developed. This approach effectively suppresses various negative regulatory molecules, including inhibitory receptor (IR) ligands like PD-L1 and IDO2, in DCs. As a result, it enhances the immune response against tumors, increases cytokine production, and decreases T cell apoptosis in breast cancer cells (Zhang M. et al., 2020). Moreover, MiR-5119 has the ability to function as a possible controller of PD-L1 in the immunological tolerance of tissue transplants (Zhou et al., 2023b). In summary, miR-5119 has the ability to selectively bind to certain regions in the 3′UTRs of PD-L1 and IDO2 genes, resulting in the inhibition of protein production for both genes. The dual-luciferase reporter test shown that the miR-5119 mimic can decrease luciferase activity in comparison to the control miRNA. This suggests that miR-5119 has the ability to directly bind to and affect the expression of the PD-L1 gene (Zhang M. et al., 2020). Therefore, miR-5119 is being seen as a potential enhancer for ICIs-based immunotherapy. However, additional research is necessary before it can be applied in a clinical setting.

MiR-200a: MiR-200a, a constituent of the miR-200 family, exhibits aberrant upregulation in breast cancer tissues (Kim, 2021). Patients with stomach cancer who have a greater level of miR-200a expression have a more favorable prognosis in comparison to those with lower miR-200a expression. This suggests that miR-200a has a significant role in predicting prognosis (Zhang H. et al., 2021). Additionally, it has been discovered that miR-200a is inversely correlated with CD86 in gastrointestinal cancer, suggesting that it could be a promising target for therapeutic intervention (Zhang H. et al., 2021). MiRNA has been demonstrated to have superior diagnostic and therapeutic performance in relation to breast cancer [118,119]. Furthermore, in relation to breast cancer, new findings have shown that the genes that miR-200a could potentially affect are highly present in the PD-1 and PD-L1 pathways. This suggests that miR-200a may have the ability to modulate these immune checkpoints (Kim, 2021). Cancer immunotherapy faces obstacles within the TME due to immune-regulatory cells and their secretions impeding antitumor immunity (Segal and Slack, 2020). MiRNAs modulate immune cell recruitment, function, and trafficking in the TME, impacting the immune contexture by influencing the polarisation of tumor-associated macrophages (TAMs) and modulating chemokines for immune cell recruitment. Moreover, extracellular vesicles transport miRNAs, mediating intercellular communication in the TME and influencing immune cell functions and responses. Understanding miRNA regulation in the TME comprehensively may yield innovative approaches to enhance cancer immunotherapy.

#### MiRNA-based therapies—inhibitors

MiRNA inhibitors are synthetic oligonucleotides designed to bind specifically to certain miRNAs, preventing them from interacting with target mRNAs. These miRNA inhibitors are also known as anti-miRNAs or antimiRs. It is feasible to decrease cancer cell growth and survival by reactivating the expression of tumor suppressor genes that have been rendered inactive by oncogenic miRNAs. MiRNA inhibitors come in a variety of forms, including locked nucleic acids (LNAs), chemically modified antisense oligonucleotides, and small molecule inhibitors. These inhibitors can be delivered into cancer cells in a variety of ways, including liposome- or nanoparticle-based delivery systems. Table 4 demonstrates the summary of therapeutic strategies for miRNAs in breast cancer.

**TABLE 4:** Therapeutic strategies for miRNAs in breast cancer.

Therapeutic strategies	Intervention (*In vitro/in vivo*)	Outcome	References
miRNA’s role in tumor progression			
miR-21 inhibition	*In vivo*	Decreased the HIF-1a/VEGF/VEGFR2 pathway and significantly slowed breast tumor growth and decreased angiogenesis	Grimaldi et al. (2021)
Targeting miR-21 in breast cancer cells using anti-miRNA oligonucleotides	*In vivo* mouse model	Decreased tumor growth and metastasis	Si et al. (2007)
miR-155 inhibition	*In vivo* xenograft models	Reduced tumor cell proliferation and invasiveness	Kong et al. (2014)
Combining miRNA inhibitor with chemotherapy	*In vivo* cell cultures	Synergistic effect, increased apoptosis	Baldassari et al. (2018)
Delivery of miR-155 expression using small molecule inhibitors	*In vitro and in vivo*	Induction of apoptosis and inhibition of tumor growth	Babar et al. (2012)
Targeting miR-200 family members using miRNA sponges	*In vivo*	Reversal of epithelial mesenchymal transition and inhibition of metastasis	Cavallari et al. (2021)
miR206, miR 335 as tumor suppressor	N/A	Suppress breast cancer, suppress metastasis and cell migration	Dong et al. (2018)
miR-replacement therapy (miR 4306 mimic)	*In vivo* breast cancer model tested Orthotropic xenograft mouse	Inhibited TNBC cell growth, lung metastasis, angiogenesis and lymphatic metastasis	Zhao et al. (2019)
MiR-138	*In vivo*	Regulate the expression of PD-1 and CTLA-4 in gliomas	Skafi et al. (2020)
miRNA’s role in the tumor immune microenvironment			
The use of miRNA inhibitor against interleukin in breast cancer	*In vivo*	Increase immune response compared to untreated cells	Cai et al. (2018)
miR-155	*In vivo*	Enhances CTL function by promoting proliferation, effector function and memory formation, cytotoxicity against tumor cells, and IFN-γ production	Cortez et al. (2019)
MiR-124	*In vivo*	Has the ability to target STAT3	Cheng et al. (2015)
miR-124 mimic	*In vivo*	Increases pro-immunogenic cytokines, such as IFN-γ, TNF-α, and IL-2 in glioma microenvironment, resulting in a potent anti-glioma therapeutic effect	Cortez et al. (2019)
miR-424	N/A	Acts as suppressor of PD-L1 and CD80 expression	Cortez et al. (2019), Skafi et al. (2020)
Disruption of PD-L1/PD-1 and CD80/CTLA-4 immune checkpoint signaling and reversion of chemoresistance mediated by restoration of miR-424 result in a synergistic effect	*In vivo*	Induces proliferation of functional cytotoxic CD8^+^T cells, the inhibition of myeloid-derived suppressive cells and regulatory T cells, and increases survival in a mouse model of ovarian carcinoma	Cortez et al. (2019), Skafi et al. (2020)

#### Anti-miRNAs or AntimiRs

AntimiRs are a family of medicinal compounds that have been engineered to block the action of certain miRNAs within cells. They are also known as anti-miRNAs and miRNA inhibitors. Due to the fact that particular miRNAs typically operate as oncomiRs or tumor suppressors, antimiRs are now being researched for their potential utility in the treatment of cancer. One such strategy is to inhibit oncomiR-21, which is commonly found to be overexpressed in malignancies such as breast, lung, and colorectal cancers. The use of antimiRs to decrease miRNA expression requires oligonucleotide optimisation with the goals of increasing binding affinity, improving nuclease resistance, and facilitating *in vivo* transport. This can be achieved by a variety of chemical modifications, including shifts in the sugar, nucleobase, or internucleotide linkages (Chakrabortty et al., 2023).

Chemical modifications, such as 2′-O-methyl (2′-O-Me), 2′-methoxyethyl (2′-MOE), and locked nucleic acid (LNA), can increase the binding affinity and resist degradation. Other changes to the backbone, such as phosphorothioate (PS) links or morpholino oligomers, can further boost nuclease resistance. Studies have shown that high-affinity modifications, such as LNA/2′-O-Me mixmers, can increase the efficacy of antimiR, with LNA having the greatest affinity of any of these modifications. High-affinity modifications like LNA/2′-O-Me mixmers, are more effective in inhibiting miRNAs such as miR-21. LNA/2′-O-Me and 2′-fluoro (2′-F)/MOE modified antimiRs show effective targeting of miR-122. AntimiRs that are fully complementary are widely utilised, and targeting the miRNA seed region is critical for suppression. Tiny LNAs are short seed-targeting LNA oligonucleotides that block complete miRNA seed families in a concentration-dependent manner. The strong binding affinity of completely substituted 8-mer LNAs is critical for successful miRNA suppression, highlighting the significance of targeting the miRNA seed for therapeutic efficacy. In both cancer and viral infections, effective miRNA targeting has been proven, which highlights the necessity of particular alterations and targeting methodologies for best treatment results.

The *in vivo* delivery of antimir Oligonucleotide shows MiRNA function was initially inhibited in worms using a 2′-O-Me oligonucleotide that mimicked miRNA loss. Antagomirs, which are 3′cholesterol-conjugated, 2′-O-Me oligonucleotides, efficiently repressed miRNAs such as miR-16 and miR-122 in mice, impacting diverse organs. PS backbone changes enhanced pharmacokinetics, allowing chemically modified antimiRs to be delivered *in vivo* (Stenvang et al., 2012). Unconjugated 2′-F/MOE, 2′-MOE, and LNA-modified antimiRs with a full PS backbone effectively silenced miRNA *in vivo*. Notably, a high-affinity 15-nucleotide LNA/DNA mixmer PS oligonucleotide targeting miR-122 displayed effective and specific silencing in mice and African green monkeys, decreasing blood cholesterol without harm. Tiny 8-mer LNA-antimiRs with great binding affinity have recently been investigated. MiR-122 and miR-21 were efficiently sequestered in mouse tissues after systemic distribution of these small antimiRs, indicating the possibility for *in vivo* functional research of miRNAs.

#### LNA oligonucleotides

LNA oligonucleotides, also known as locked nucleic acid oligonucleotides, are the antisense agents of the next-generation. The third-generation nucleic acid analogue, Locked Nucleic Acid (LNA), has emerged as a promising tool in molecular medicine and cancer research. As a result of its unique properties, LNAs have a greater binding affinity, sequence specificity, temperature stability, and nuclease resistance (Kamali et al., 2022). Oligonucleotides that contain one or more nucleotide building blocks and have an additional methylene bridge that locks the ribose moiety into either the C3′-endo (beta-D-LNA) or C2′-endo (alpha-L-LNA) conformation are referred to as having locked nucleic acid, or LNA for short. Locked nucleic acid is a kind of nucleic acid.

Its unique features make it ideal for the synthesis of nucleic acid-based medicines. LNA is used in cancer therapy in a variety of ways, such as oligonucleotide analogues and mimics. LNA nucleotides, for example, are used to construct mimic structures that make strong interactions with target proteins, as observed in inhibiting KRAS gene expression in pancreatic cancer. Furthermore, LNA is effective in silencing disease-associated miRNAs, suggesting a new path for targeted cancer therapy. LNA has been used to inhibit miR-21 in melanoma cells, resulting in lower proliferation and greater apoptosis, and LNA-i-miR-221 has shown potential in treating multiple myeloma. Furthermore, LNA-containing aptamers and LNA-based diagnostic procedures such as PCR improvements and single nucleotide polymorphisms discrimination help cancer research by enhancing target delivery and detection sensitivity. These applications highlight LNA’s versatility and therapeutic potential in cancer research and treatment, holding great promise for future studies, clinical applications, and diagnostic contexts, highlighting its potential impact on cancer treatment and detection methodologies.

#### Small molecule inhibitors

The major approaches of targeted cancer treatment are antibody therapies and small molecule inhibitors. Small molecule inhibitors decrease target protein function by attaching to the “pocket” on their surface. In compared to antibodies, their smaller size allows them to attach to a greater spectrum of sites, both extracellular and intracellular. Furthermore, many small molecule inhibitors can be given orally, whereas antibodies must be administered subcutaneously or intravenously (Liu et al., 2022). To treat intracranial lesions, certain small molecule inhibitors can even cross the blood-brain barrier. Protein kinase inhibitors are the most common of these medications, although they also affect DNA repair, epigenetics, apoptosis, and tumor metabolism. Notably, recent approvals have expanded to previously deemed difficult goals, such as RAS. Small molecule inhibitors are an important component of targeted cancer therapy because to their versatility and growing scope.

The US FDA has authorised 88 small molecule inhibitors for cancer indications as of August 2022. These inhibitors are classified into selective small molecule inhibitors and multikinase small molecule inhibitors depending on target selectivity. Selective small molecule inhibitors, further subdivided into kinase and non-kinase inhibitors, target particular cancer growth, survival, apoptosis, and metabolic dysfunctions. Because of their potential to target particular genes, patients must be screened for gene changes. Multikinase inhibitors, mostly targeting VEGFR, have anticancer effects that do not need exact detection. Protein kinase inhibitors, a major class of small chemical inhibitors, target the human kinome, which includes both eukaryotic and atypical protein kinases. These inhibitors prevent ATP from attaching to kinases, which is required for signal transmission. Based on their biochemical mechanisms, protein kinase inhibitors are classified into six types: those that interact with the ATP binding site (Type I and II), those that bind allosterically (Type III and IV), those that interact with both allosteric and ATP binding pockets (Type V), and those that form irreversible covalent bonds (Type VI). Each variety has distinct features, allowing for customised cancer treatment techniques.

### Therapeutic potential of MiRNA in cancer immune response

**FIGURE 9:**
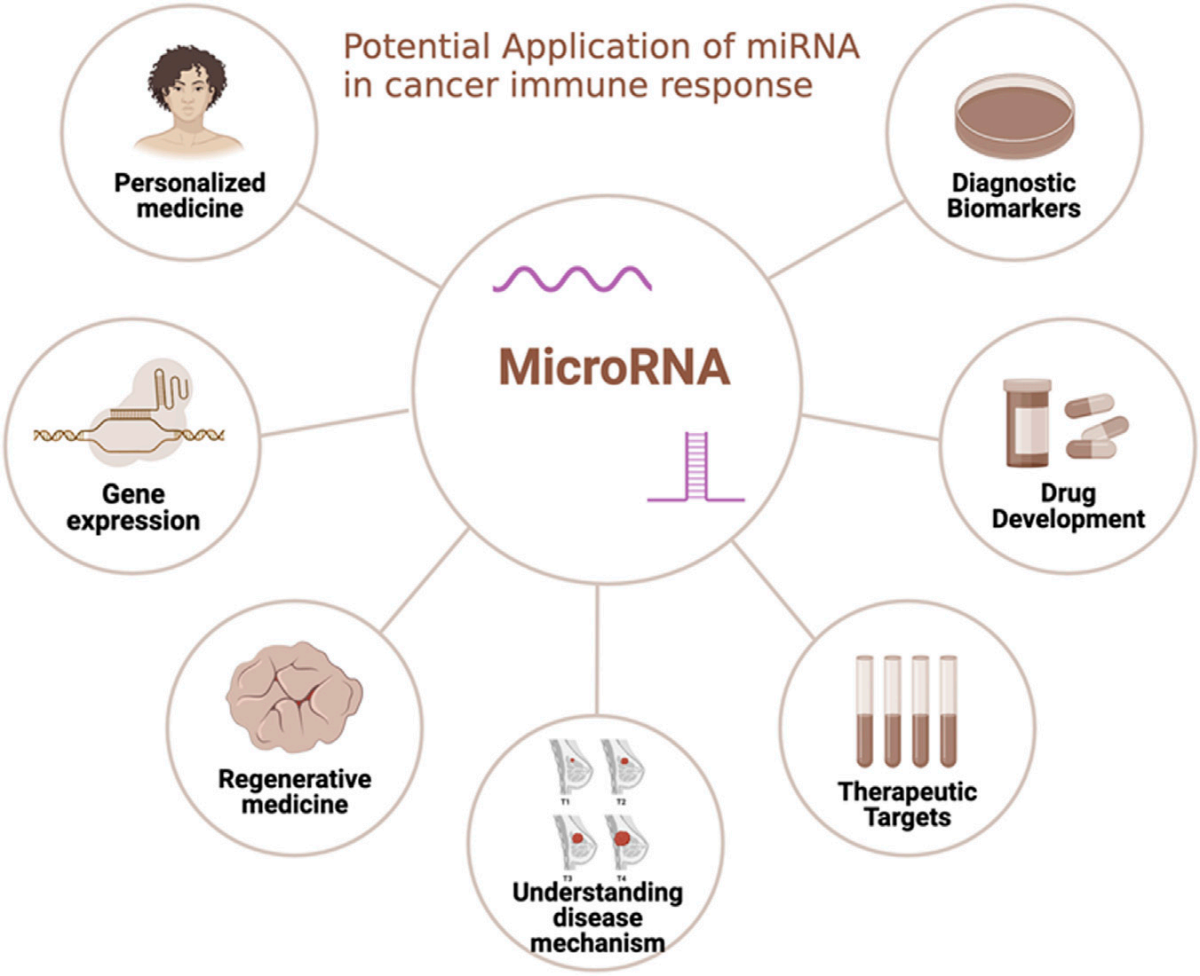
Therapeutic applications of miRNA in cancer immune response.

Emerging as highly adaptable molecules, miRNAs have a wide range of possible uses that go beyond just the medical field. In addition to their well-established function in cancer-related immune suppression, miRNAs have many other potential uses in the fields of diagnostics, prognostics, therapeutics, drug discovery, personalised medicine, regenerative medicine, and elucidating disease mechanisms across diverse medical domains (Aggarwal et al., 2020) (Figure 9).

The possible use of miRNA in early illness identification and continuous monitoring is highlighted by the fact that variations in miRNA expression patterns have been associated to several diseases, most notably cancer as such it a promising biomarker. It is important to note that miRNAs play a crucial role in the transformation of cancer cells. They are very stable in body fluids such as blood, saliva, and urine, and they exhibit unique patterns of expression that are particular to different tissues, which gives them diagnostic potential (Mehrgou and Akouchekian, 2017). Researchers compare the miRNA expression profiles of healthy persons to those of breast cancer patients by collecting tissue or blood samples. This allows them to use miRNAs as diagnostic biomarkers. Modern methods such as qRT-PCR and microarray analysis allow them to pinpoint which miRNAs are either up- or downregulated in breast cancer. Early identification, therapy response monitoring, and patient outcome prediction can all be aided by these miRNAs when utilised as biomarkers. To help in tumor subgrouping, several miRNAs like miR-30 have functions in determining ER, PR, and HER-2 status. Research has shown that more than fifteen miRNAs are involved in the spread of breast cancer and that low expression of miR-125b and miR-126 is associated with poor survival rates. Stem cells, one of several cell types seen in breast tumors, play an important role in tumor progression because of their special capacities. MiRNAs impact the G1/S cell cycle transition, regulate stem cell populations, and govern self-proliferation and cell destiny in stem cells. They may regulate many genes at once (McGuire et al., 2015). The function of miRNAs in cancer research has been better understood in recent years, and this has led to new insights into molecular subgrouping, the identification of breast cancer risk genes, and the development of diagnostic and prognostic biomarkers.

Furthermore, some miRNA signatures have demonstrated robust correlations with illness prognosis and patient outcomes, providing vital information on the development of the disease, the efficacy of treatments, and the total percentage of survival. A potential way to evaluate prognosis in a wide range of medical disorders is by profiling miRNA expression levels. MiRNAs have the ability to inhibit the advancement of the cell cycle or the creation and growth of tumors, respectively, which makes them either oncogenes or tumor suppressors. Since the discovery of miRNAs, there has been a significant amount of interest in the possibility of using them in the diagnosis, prognosis, and therapy of cancer. There are many different forms of tumors, and it is feasible to detect their individual miRNA profiles. It is possible that these profiles might be utilised as phenotypic indicators to assist in the processes of cancer diagnosis, prognosis, and treatment. As mentioned already, miRNAs impact biological processes such as cell migration, proliferation, and tumor formation; they are found in genomic areas that are either delicate or connected to cancer. There are many mechanisms by which oncogenic miRNAs aid in the progression and maintenance of tumor cells (McGuire et al., 2015). As an example, miR-21 is increased in breast cancer and inhibits tumor suppressor genes; in TNBC, on the other hand, miR-155 enhances angiogenesis and invasiveness. The potential therapeutic significance of tumor suppressor miRNAs like miR-126 and miR-206 is demonstrated by their ability to limit cell proliferation and metastasis. In the event that miRNA profiles are able to accurately predict malignancies, this method has the potential to revolutionise a number of diagnostic challenges.

The ability of miRNAs to serve as intervention targets is the source of their therapeutic potential. Gene expression may be changed and disease pathways can be modulated by manipulating miRNA expression levels using various tactics, such as using miRNA mimics or inhibitors (Iorio et al., 2011). This possibility has tremendous promise for developing novel treatments for a wide range of ailments, including cancer, cardiovascular issues, and neurological problems. The altered expression patterns in cancer and the tissue-specific stability in formalin-fixed paraffin-embedded tissues demonstrate the use of miRNAs as diagnostic tools. Their analysis is useful for subdividing breast cancer cases, forecasting how the illness will develop, and gauging how well patients will react to treatments like hormone replacement and chemotherapy. One possible way to improve treatment responses and overcome resistance is to manipulate miRNA expression levels. Gaining insight into the role of miRNAs in disease pathways can help in the development of medications that target these molecules or the processes that regulate them. The potential for these individualised medications to affect miRNA activity and, by extension, to open up new therapy possibilities is exciting. In addition, by customising medicines according to individual miRNA profiles, the use of miRNA profiling in personalised medicine has the potential to completely alter therapy paradigms. This personalised strategy has the ability to increase treatment effectiveness while reducing side effects. Because of their critical function in disease pathways, miRNAs hold great promise for personalised therapy. Their roles as cancer oncogenes or tumor suppressors pave the way for individualised treatments. As an example, oncomiRs can be targeted using compounds to limit their action, possibly halting cancer growth; miRNA mimics restore tumor-suppressive miRNA expression, blocking oncogenic effects (Chakrabortty et al., 2023).

**FIGURE 10:**
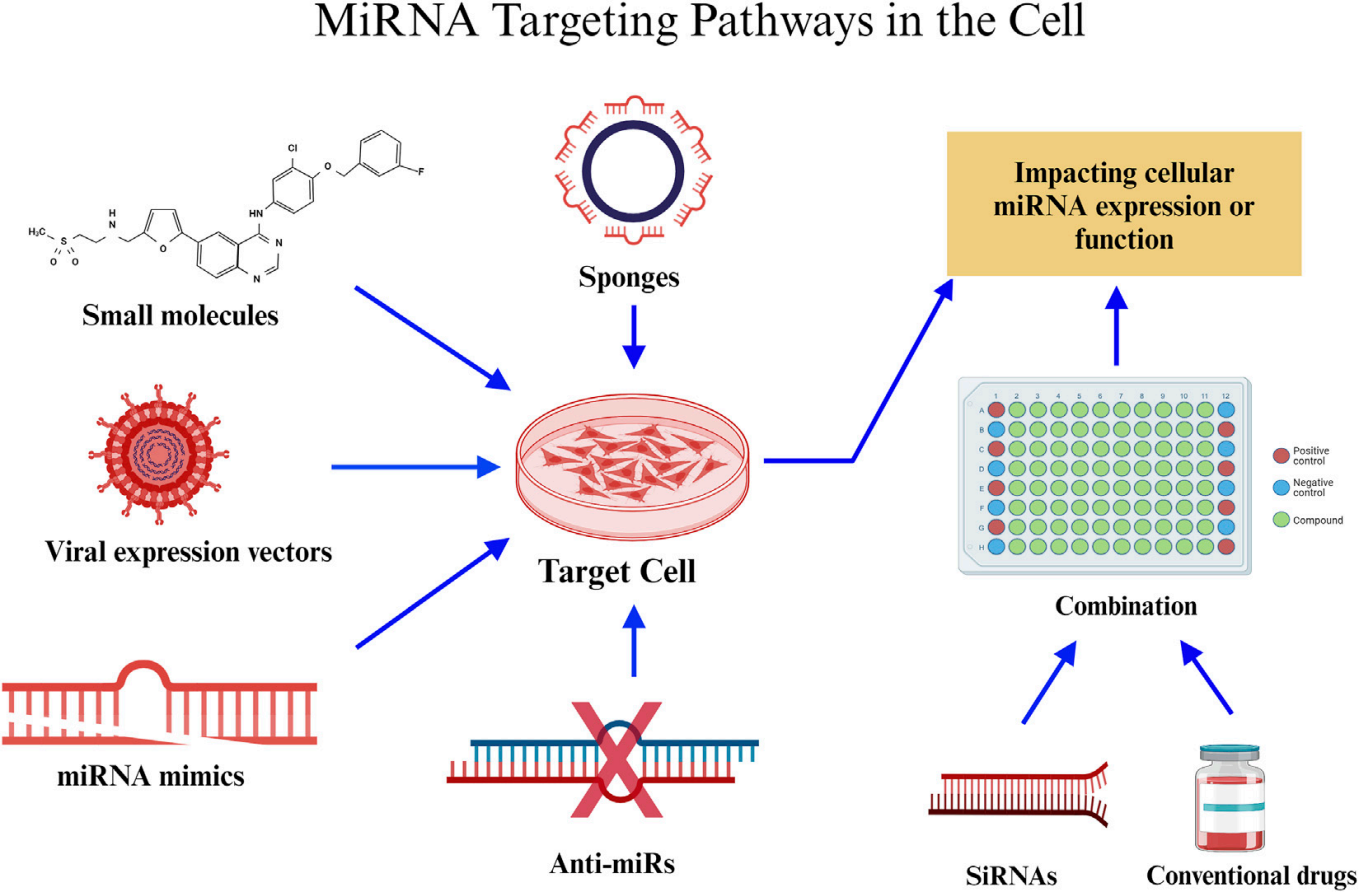
Overview of miRNA targeting pathways in the cell.

Single-stranded molecules that are essential for cellular activities, miRNAs, can be degraded inside cells. Modifying synthetic oligonucleotides stabilises and improves their function. Downregulating oncomiRs activity is a primary goal in breast cancer therapy. Because of a change at the C2 carbon of the sugar molecule, antisense anti-miR oligonucleotides (AMOs) are effective in reducing the expression of certain miRNAs. Their stability and capacity to suppress cancer growth are enhanced by various chemical modifications, such as the addition of methyl groups or fluorine. Modified AMOs may have off-target effects and are expensive. Stability and off-target impacts can be improved by combining adjustments (Wong and Cheah, 2020). To successfully limit the proliferation of cancer cells, customised AMOs were utilised in recent investigations. A different method, CRISPR/Cas9, allows for the permanent suppression of genes, which in turn lowers tumor development in breast cancer. Because miRNAs are unstable, delivering them is difficult. Systems using viruses, such as lentivirus or adenovirus, and non-viral components, such as lipids or polymers, facilitate the transport and protection of miRNAs to their intended target cells. One example is the non-toxic delivery of tumor-suppressing miRNAs to mice utilising lipid-based carriers and gold nanoparticles. Nanocarriers made of polymers work in tandem with chemotherapy to increase the effectiveness of the former while decreasing the latter’s toxicity. Figure 10 demonstrates an overview of miRNA targeting pathways in the cells.

There are many methods for therapeutically targeting certain miRNAs in cells. These strategies entail modifying the expression or function of miRNAs implicated in illness. Anti-miRNAs are used to break down or block the function of harmful miRNAs, synthetic miRNA copies that mimic natural ones, viruses are used to introduce miRNA expression, small molecules disrupt miRNA production, or miRNA sponges are used to block the action of miRNAs by diverting them away from their usual targets. In both experimental and clinical contexts, these approaches are occasionally paired with additional therapies such as siRNAs or conventional medicines. These results demonstrate that miRNAs may be useful therapeutic targets for breast cancer. The development of secure miRNA delivery methods shows potential for individualised treatments, which might improve anticancer outcomes while reducing patient adverse effects (Diener et al., 2022). The promise of miRNA-based treatments as an effective tool in the fight against breast cancer has been illuminated by a thorough examination of *in vivo* experiments. Opportunities for therapeutic intervention were revealed via categorising research into combination therapy, miRNA replacement, and miRNA inhibition. Several breast cancer models showed significant improvement in tumor development and metastasis when miRNA replacement techniques were used, with a focus on miR-34a, miR-497, miR-544, and miR-603. For example, in breast cancer cells, miR-34a replacement inhibited migration and proliferation, but in TNBC cells, miR-544 and miR-603 replacements reduced invasion and proliferation. Research into miRNA suppression has focused on oncomiRs such as miR-21 and miR-214 because of the promise they hold for preventing the advancement of cancer by modulating certain genes and signalling pathways (Sharma S. et al., 2022).

The synergistic effects of antagomiR-21 with standard chemotherapeutics were particularly striking. The results showed that miR-221 inhibition and miR-205 substitution had a synergistic impact on tumor growth suppression and metastasis mitigation. Furthermore, studies explored the use of miRNA-based therapeutics to manipulate the TME, with the goal of influencing the interactions between cancer cells inside the TME to hinder metastasis. Research has shown that reprogramming tumor-membrane endothelial cells (TMECs), targeting particular miRNAs linked with metastasis, and replacing tumor-suppressing miRNAs with other miRNAs are all effective ways to prevent the spread of metastases. The promising results of delivering miRNAs through nanosized carriers or systemic injections have sparked optimism for their potential therapeutic uses in the fight against breast cancer. These results highlight the exciting possibilities for tailored breast cancer treatment that miRNA-based therapies provide.

Another mention worthy therapeutic potential of miRNA is noticed in regenerative treatments which may be driven by manipulation of miRNA expression. Regenerative medicine relies heavily on miRNAs for a variety of purposes, including cell differentiation and tissue regeneration. Regulating gene expression and controlling diverse cellular processes are two of miRNAs’ many functions, making them a potential target for regenerative medicine. Researchers have the ability to control cell fate, improve tissue healing, and stimulate regeneration by regulating miRNA expression. MiRNAs block the translation of target genes and operates as post-transcriptional gene regulators. They have a role in a wide variety of biological activities, such as wound healing, tissue homeostasis, and embryonic development. One of the most important roles that miRNAs perform during tissue regeneration is in regulating the processes of cellular dedifferentiation, trans differentiation, and reprogramming. The use of miRNA mimics and inhibitors directed towards certain cells at the damage site allows for the manipulation of miRNA levels. By focusing on specific areas, scientists may exert fine-grained control over miRNA expression and function, which in turn allows them to modulate cellular activity and stimulate tissue regeneration. Another option for induced pluripotency that does not involve manipulating transcription factors is to use miRNA-based techniques. A powerful new method for producing regenerative pluripotent stem cells is now at our fingertips. Additionally, regenerative medicine has demonstrated translational relevance for miRNA-based treatments. By increasing the expression of beneficial miRNAs and decreasing the expression of harmful ones, these treatments can improve tissue regeneration and repair. New opportunities for the development of novel therapeutics and pharmacological techniques have emerged with the capability to precisely target miRNAs implicated in pathways for tissue regeneration (Karami F. M. et al., 2022). It may orchestrate cell destiny, enhance tissue repair, and promote regeneration.

MiRNAs have recently gained attention as a possible therapeutic tool that might help with a variety of issues, including steroid receptor modulation, metastasis, multidrug resistance, and recurrence after chemotherapy. Relapse prevention strategies that target breast tumor-initiating cells (BT-ICs) have recently gained interest. One such strategy, miRNA let-7, has shown promise in breast cancer models in mice. The use of miRNAs such as miR-22, miR-145, miR-206, and miR-375 in anti-ER-α/HER treatments have the potential to address multidrug resistance and specifically target the overexpression of ER-α and HER2/HER3. Potential anti-metastasis therapies include miRNAs such as miR-21, miR-31, miR-145, and miR-146, which have shown functions in reducing invasion and metastasis. Additionally, novel approaches to breast cancer treatment are being explored through miRNAs such as miR-155, miR-663, miR-326, miR-328, and miR-451, which impact breast cancer cell survival, sensitivity to chemotherapeutic agents, and the reversal of drug resistance mechanisms (Zhang K. et al., 2014).

## Therapeutic implications of targeting dysregulated MiRNAs in breast cancer

Dysregulation of miRNAs is a crucial factor that affects several cellular pathways in breast cancer. These routes include those that regulate cell proliferation, the cell cycle, and the response to treatment. The presence of genetic instability complicates both carcinogenesis and the therapy of breast cancer, as it hinders the capacity to specifically target the pathways responsible for the development and proliferation of cancer cells. However, targeting dysregulated miRNAs in breast cancer has intriguing therapeutic implications for resolving drug resistance and increasing patient outcomes. To develop effective personalised therapeutic strategies for breast cancer treatment, it is essential to comprehend the intricate regulatory networks involving miRNAs and their downstream targets. Enhancing the efficacy of existing medications and enabling personalised strategies for breast cancer therapy may be achieved by harnessing the therapeutic capabilities of dysregulated miRNAs.

The aberrant expression of miRNAs in breast cancer has a substantial influence on the efficacy of therapeutic treatments and plays a role in the genesis of cancer. MiRNA dysregulation leads to resistance to breast cancer treatment, which is caused by changes in drug transporters and an increase in antiapoptotic activity. Oncogenic miRNAs, such as miR-155 and miR-21, have been shown to have a role in regulating resistance-associated proteins, which ultimately leads to the development of chemoresistance. Multiple treatment methods for breast cancer, including as targeted treatments, chemotherapy, antiendocrine therapy, and radiation, have been associated with resistance based on miRNA research. For example, the decrease in miR-26a and the increase in E2F7 in oestrogen receptor-positive (ER+) breast cancer highlight the possibility for treating the disease by targeting miRNAs that are not functioning properly (van Schooneveld et al., 2015). Specifically, the promising approach of targeting dysregulated miRNAs such as miR-26a and miR-30b has been found to enhance the efficiency of trastuzumab therapy for HER2-positive (HER+) breast cancer by improving sensitivity (Liu J. et al., 2018). In breast cancer tissues, the expression of miR-365 and miR-22 is reduced. These two molecules, which have lower levels of expression, might be targeted for therapy to enhance the effectiveness of chemotherapy therapies. Overexpressing miR-365 and miR-22 via the use of miRNA mimics inhibits the growth of breast cancer cells and enhances their susceptibility to chemotherapeutic treatments by selectively targeting key proteins involved in cell proliferation and tumor development (Loh et al., 2019; Bautista-Sánchez et al., 2020b).

Moreover, miR-221/222 have been identified as important regulators of resistance to antiendocrine therapy, specifically targeting proteins that are involved in signalling via the oestrogen receptor and control of the cell cycle. Targeting dysregulated miRNAs, such as miR-708, which are triggered by the glucocorticoid receptor alpha (GRα), offers a promising therapeutic approach to prevent breast cancer progression and improve the efficacy of chemotherapy. The presence of miR-210, miR-9, miR-187, and miR-155 in breast cancer indicates that they may be targeted to enhance the effectiveness of therapy and decrease the advancement of the disease (Mulrane et al., 2013). The identification of dysregulated miRNAs has promise for developing personalised therapeutics for breast cancer, despite the nascent stage of this research field. Clinical trials investigating the impact of drugs on miRNA demonstrate potential for personalised treatment approaches; miRNA-based therapeutics provide a novel approach to enhance outcomes for breast cancer patients.

### MiRNA therapeutics in clinical trials

Despite the considerable potential inherent in the clinical application of medicines that regulate miRNA expression, the current absence of approved miRNA pharmaceuticals and phase 3 research documented on Clinicaltrials.gov is noteworthy in breast cancer. The siRNA therapeutics, which belong to a closely related class, have demonstrated a modestly higher level of success in their progression towards clinical implementation. This underscores the numerous obstacles that must be overcome in order to effectively advance the development of an oligonucleotide therapy. The challenges associated with the development of miRNA drugs for cancer treatment are clearly evident in the ongoing clinical trials. The website Clinicaltrials.gov provides a comprehensive compilation of Phase 1 and 2 clinical trials investigating the efficacy of miRNA compounds in the treatment of various diseases, including as diabetes, cardiovascular diseases, hepatitis C virus infection, and cancer, exhibiting varying outcomes. An illustrative instance of a miRNA drug that had challenges in clinical trials is MRX34 developed by Mirna Therapeutics, Inc. The administration of a liposomal injection of a synthetic double-stranded RNA oligonucleotide was conducted in patients diagnosed with primary liver cancer. The purpose of this intervention was to replace the depleted miR-34 and reinstate its functionality on the p53/wnt cellular pathways. Nevertheless, the study was concluded as a result of immune-related severe adverse events, leading to the withdrawal of a planned phase 2 study involving melanoma patients. Other clinical research investigating medicines targeting dysregulated miRNAs in cancer yielded more encouraging outcomes. The development of targeted minicells carrying targomiRs, a miRNA mimic, was a collaborative effort between the Asbestos Diseases Research Foundation and EnGeneIC Limited. A phase 1 research was conducted to evaluate the efficacy of the initial TargomiR, Mesomir 1, in individuals diagnosed with malignant pleural mesothelioma. The Mesomir one protein harbors a miRNA mimic those functions as a tumor suppressor for miR-16 in several types of cancer. An anti-EGFR bispecific antibody is used to specifically target lung cancer cells that express EGFR. The authors of the study assert that further investigations are warranted due to the drug’s satisfactory safety profile and efficacy (Reid et al., 2016; Van Zandwijk et al., 2017).

Regulus has announced the introduction of another therapeutic candidate targeting a dysregulated miRNA in cancer. The medication RGLS5579 is designed to specifically target miR-10b in glioblastoma multiforme, a therapeutic target that has shown promise (Teplyuk et al., 2016). The primary objective of the present investigation is to validate the prognostic and diagnostic use of miR-10b expression patterns in glioma samples. Additionally, the researchers intend to assess the sensitivity of individual primary tumors to anti-miR-10b treatment *in vitro*. A favorable study result would facilitate the entry of their medicine into clinical trials. Moreover, several miRNA medicines currently undergoing clinical trials for various illnesses are providing optimism on the potential application of similar techniques in the treatment of cancer. The ability of a single class of miRNA medications to effectively treat various diseases is demonstrated by locked nuclear acid (LNA)-based anti-miRNAs. These drugs have shown promise as a potential class of miRNA drugs, as evidenced by human phase 1 trials that confirmed the safety and efficacy of three LNA-based anti-miRNAs. One example is Miravirsen (SPC3649 from Roche), which specifically targets the liver-specific miR-122 (Gebert et al., 2014). In a phase 2 study including patients with chronic HCV genotype 1 infection, Miravirsen demonstrated sustained decreases in hepatitis C virus (HCV) RNA levels without any indication of viral resistance (Janssen et al., 2013).

Cobomarsen (MRG-106), a second LNA-based anti-miRNA, has been demonstrated to be safe in humans. It specifically targets miR-155 in several hematological malignancies (Foss et al., 2018). Presently, there exist two ongoing phase 2 clinical trials investigating the potential application of Cobomarsen in the treatment of cutaneous T-cell lymphoma. MRG-110, the most recent among the three anti-miRNA medication possibilities, functions by inhibiting miR-92a. Its primary objective is to stimulate angiogenesis and perhaps be employed in the therapy of heart ailments. The results of a phase 1 trial shown that MRG-110 effectively decreases the levels of miR-92a and suppresses the expression of miR-92a target genes in the peripheral blood compartment. The authors suggest that targeting gene expression in T cells and NK cells may have therapeutic potential in disorders characterized by dysregulated immune processes (Abplanalp et al., 2020). This finding could also be relevant in the setting of tumors. Abivax employs a promising strategy to regulate miRNA expression in illnesses. Two phase 2 trials are now being conducted to evaluate the efficacy of ABX464, a chemical, in individuals diagnosed with Crohn’s disease or moderate to severe active ulcerative colitis, a condition that frequently advances to colon cancer. The overexpression of miR-124 is induced by the administration of ABX4664, which binds to the cap binding complex located at the 5′-end of pre-mRNA. This binding process leads to the enhancement of splicing of a single long noncoding RNA, resulting in the production of the anti-inflammatory miR-124 (Vautrin et al., 2019). UniQure Biopharma B.V. has successfully created a gene silencing method known as miQURE™, which utilizes artificial micro-RNAs. AAV5 vector was developed to treat Huntington’s disease. This vector contains a miQure miRNA that specifically targets the huntingtin gene (AMT-130). The safety and proof of concept of this miRNA are now being evaluated in a phase 1/2 trial (Rodrigues and Wild, 2020). If the approach demonstrates both safety and efficacy in human subjects, it is likely to be applicable for cancer therapy as well.

### MiRNA therapeutics to reverse TME chemotherapy resistance

The interaction between tumor cells and stromal cells via miRNAs can also improve treatment resistance. A study found that propofol causes TAMs to release miR-142-3p, which conveys propofol action in cancer cells, demonstrating that modulating miRNA transfer in the TME can be employed for treating cancer. Propofol was initially shown to decrease tumor growth in tumor-bearing mice in an HCC model. After researching the mechanism, it was shown that these effects were mediated by the transport of miR-142-3p via secreted microvesicles from TAMs during propofol stimulation (Zhang J. et al., 2014). Thus, interfering with miRNAs involved in TME-mediated therapy resistance is a potential way to reverse resistance and could be attempted in a variety of scenarios, including miRNA exchange between tumor cells and CAFs, TAMs, or other stromal or cancer cells, as well as miRNAs involved in signaling that confers chemotherapy or immunotherapy resistance.

Additional stromal cells within the TME also exert an impact on the development of resistance to therapy. Research has demonstrated that the administration of doxorubicin resulted in the increased expression of miR-21-5p in both mesenchymal stem cells and the exosomes produced from them. In breast cancer cells, the expression of S100A6 was promoted by miR-21-5p generated from exosomes, leading to the development of chemoresistance both in laboratory settings and in living organisms (Luo et al., 2020). In the majority of the aforementioned scenarios, a therapeutic strategy would probably involve manipulating the levels of miRNAs to counteract chemotherapy resistance and enable the continuation of the treatment. Nevertheless, as exosomal transfer promotes miRNA-mediated chemotherapy resistance in certain instances, interrupting this pathway of transfer could significantly contribute to the prevention of resistance from occurring initially (Raue et al., 2021).

### Co-delivery

Multiple studies have demonstrated that co-administering the miRNA drugs alongside a chemotherapeutic agent can enhance the effectiveness of the treatment. Docetaxel, another cytostatic, has also been used to deliver miR-21 in TNBC. Experiments conducted in a laboratory setting demonstrated that the susceptibility of TNBC cells to docetaxel treatment was enhanced following treatment with a substance known as chitosomes. Chitosomes are nanovectors that self-assemble and have a core-shell structure. They are used to transport anti-miR-21 and docetaxel (Sun et al., 2021). In addition, there have been advancements in the development of delivery vehicles that can co-deliver miR-21 inhibitor and doxorubicin or epirubicin. Some of these vehicles have demonstrated synergistic anti-cancer effects of the therapeutics (Bahreyni et al., 2019; Song et al., 2020), while others have shown the ability to overcome multi-drug resistance (Zhi et al., 2013). There are also vehicles that exhibit high delivery efficiency but do not have a synergistic effect (Qian et al., 2014).

Ren et al. developed nanoparticles to achieve sequential drug delivery, which is crucial for the synergistic effectiveness of miRNA inhibitors and chemotherapeutic drugs (Ren et al., 2016). The researchers utilized a method involving hollow gold nanoparticles (HGNPs) that respond to near-infrared light (NIR). These nanoparticles were modified with PAMAM and loaded with a miR-21 inhibitor and doxorubicin. The purpose was to specifically target breast cancer cells in laboratory tests and in a mouse model where human cancer cells were transplanted. The miR-21 inhibitor was delivered in a sequential manner by utilizing the proton sponge effect of the PAMAM polymer once the nanoparticle was taken up through endocytosis. Following a duration of 4 h, the NIR treatment caused the hollow gold-nanoparticles to collapse, thereby releasing the enclosed doxorubicin into the sensitive cancer cells. The anticancer effectiveness was enhanced four times when compared to treatment with just doxorubicin following intravenous administration, indicating the potential of this sequential delivery approach for cancer therapy (Ren et al., 2016).

The tumor suppressor miR-34a, which plays a significant role in inhibiting tumor growth, is frequently employed in combination therapy alongside chemotherapeutic drugs. The downregulation of miR-34a is commonly associated with the development of resistance to chemotherapy. In the same manner, the administration of miR-34a and docetaxel using nanocarriers suppressed the growth and spread of tumors in a mouse model of metastatic breast cancer (Zhang L. et al., 2017). Administering doxorubicin with miR-34a demonstrated anti-tumor effects in prostate (Yao et al., 2016) and breast (Deng et al., 2014) cancer cells both in laboratory settings and in living organisms. The Table 5 lists several other miRNAs that have been utilized in co-delivery systems alongside chemotherapeutics.

**TABLE 5:** Pre-clinical research on the co-delivery of miRNAs and chemotherapeutics in various cancer.

MiRNA therapeutic	Chemotherapeutic agent	Delivery vehicle	Type of cancer	Ref
miR-7	Paclitaxel	PEG-PLGA-poly (l-lysine) nanoparticles	Ovarian cancer	Cui et al. (2018)
miR-10 inhibitor	Paclitaxel	pH-sensitive liposomes	Breast cancer	Zhang et al. (2015b)
Mir-21 inhibitor	Doxorubicin	Core-shell tecto dendrimers	Breast cancer	Song et al. (2020)
		NIR-responsive hollow gold nanoparticles	Breast cancer	Ren et al. (2016)
		Amphiphilic copolymers	Glioma	Qian et al. (2014)
		Graphene oxide-based nanoparticles	Breast cancer	Zhi et al. (2013)
	Epirubicin	MUC1 aptamer-targeted poymers	Breast cancer	Bahreyni et al. (2019)
	Docetaxel	Chitosome	TNBC	Sun et al. (2021)
	Cisplatin	Nano-Graphene oxide nanoparticles	Lung cancer	Liu et al. (2018b)
	5-FU	Engineered exosomes	Colon cancer	Liang et al. (2020)
	5-FU	Poly (amidoamine) dendrimers	Human glioma	Ren et al. (2010)
	Gemcitabine	Dendrimer-entrapped gold particles using UTMD	Pancreatic cancer	Lin et al. (2018)
		PEG-PEI magnetic iron oxide nanoparticles	Pancreatic cancer	Li et al. (2017d)
	Pemetrexed	Lipid-polymer hybrid nanoplexes	Glioblastoma	Küçüktürkmen et al. (2017)
		Cationic lipid nanoparticles	Glioblastoma	Küçüktürkmen and Bozkır (2018)
	Sorafenib	Dual targeting reconstituted HDL	Hepatocellular carcinoma	Li et al. (2018)
	4-Hydroxy-tamoxifen	PLGA-b-PEG nanoparticles	Breast cancer	Devulapally et al. (2015)
miR-29b	Retinoic acid	Micellar nano system	NSCLC	Magalhães et al. (2020)
miR-31	Doxorubicin	Stimuli-responsive silica nanoparticles	HeLa cell line/tumors	Wang et al. (2018b)
miR-34a	Doxorubicin	Hyaluronic acid chitosan nanoparticles	TNBC	Deng et al. (2014)
		Cationic polypeptide-based micelles	Prostate cancer	Yao et al. (2016)
	Paclitaxel	Lipid nanoparticles	Melanoma lung metastases	Shi et al. (2014)
	Irinotecan	Polymeric hybrid micelles	Colorectal cancer	Li et al. (2020b)
	Notch1 antibody	PLGA nanoparticles	TNBC	Valcourt and Day (2020)
tRNA-mir-34a	Doxorubicin	Creatin-based polymer	Breast cancer lung metastases	Xu et al. (2019)
miR-34a activator rubone	Docetaxel	Dual responsive micelles	Prostate cancer	Lin et al. (2019)
miR-122	Doxorubicin	Gold nanocages	Hepatocellular carcinoma	Huang et al. (2018b)
	5-FU	Chitosan nanoparticles	Hepatocellular carcinoma	Ning et al. (2019)
miR-181a	Melphalan	Lipid nanoparticles	Retinoblastoma	Tabatabaei et al. (2019)
miR-200	Irinotecan	pH-sensitive and peptide-modified	Colorectal cancer	Juang et al. (2019)
		liposomes and solid lipid		
		nanoparticles		
miR-200c	Docetaxel	Gelatinases-responsive nanoparticles	Gastric cancer	Liu et al. (2013)
miR-205	Gemcitabine	Cationic copolymers	Pancreatic cancer	Mittal et al. (2014)
miR-218	Temozolomide	Folate-chitosan gel-delivered gold nanoparticles	Glioblastoma and lung cancer	Fan et al. (2015)
miR-221/222 inhibitors	Paclitaxel	Calcium phosphate-polymer hybrid nanoparticles	TNBC	Zhou et al. (2017b)
miR-345	Gemcitabine	Polymeric dual delivery nanoscale device	Pancreatic cancer	Uz et al. (2019)
miR-375	Cisplatin	Lipid-coated nanoparticles	Hepatocellular carcinoma	Yang et al. (2016)
miR-451	Doxorubicin	Coated calcium carbonate nanoparticles	Multidrug resistant bladder cancer	Wei et al. (2020)
miR-542-3p mimic	Doxycycline	HA/PEI-PLGA nanoparticles	TNBC	Wang et al. (2016)
miR-1284	Cisplatin	CD59 receptor-targeted liposomes	Cervical cancer	Wang and Liang (2020)

### Challenges and future direction

While studying miRNAs as critical regulators of tumor processes in breast cancer, a number of impediments to their efficient diagnostic and therapeutic usage were discovered. To begin, preanalytical and analytical difficulties such as sample selection bias, haemolysis interference, and uneven extraction methods may all reduce the accuracy of miRNA quantification in research. The lack of established standards for sample collection, handling, and processing hinders data interpretation, making the identification of clinically meaningful miRNA biomarkers even more challenging. Biomarker validation efforts are further confounded by the fact that global normalisation processes do not fully account for variations in miRNA expression levels caused by cancer, lowering the reliability of miRNA profiles in the bloodstream. Furthermore, miR-21 dysregulation in other diseases raises questions about its specificity in cancer detection, potentially limiting its utility as a biomarker for breast cancer. Because of this widespread dysregulation, miR-21 is more likely to participate in non-cancer cellular stress responses. One potential solution to these challenges is to use tissue-specific miRNAs in conjunction with miR-21 in a complete biomarker collection cancer (Tiberio et al., 2015). This will improve specificity and validate the carcinogenic process in the afflicted tissues. Technique standardisation is still necessary, since differences in pre-processing procedures lead to inconsistent miRNA expression measurements. In addition, the complicated nature of cancer and the wide control of miRNAs provide challenges to therapies based on miR-21. The complex roles of target genes raise questions about the efficacy of miR-21 treatments, even if some of its targets have been shown to work. Further research on the metabolic byproducts of miRNA-based treatments is also required due to worries of toxicity and long-term consequences. Regardless, if these obstacles can be overcome, miR-21 has the potential to become an important biomarker and treatment for cancer and other complicated disorders. Future study and development of miR-21 indicate great potential as a tool for the diagnosis and treatment of illnesses, including cancer (Bautista-Sánchez et al., 2020b).

In the future, the study of miRNAs and their function as crucial controllers of breast cancer tumors is expected to adopt an interdisciplinary methodology. In order to enhance the practical significance of studying circulating miRNA in breast cancer treatment, it is essential to establish standardised protocols for sample collection, processing, and interpretation of results. This will ensure that the investigations are able to be replicated and trustworthy (Tiberio et al., 2015). Moreover, it is necessary to conduct functional studies and comprehensive experimental validation in order to further investigate the roles of miRNA-gene interactions in the communication between tumors and immune cells. In order to get a deeper understanding of the molecular pathways implicated in the progression of breast cancer, scientists are using both *in vitro* and *in vivo* investigations to validate the accuracy of hypothesised networks that regulate the interaction between miRNA and genes. Essential steps in accurately evaluating variations in miRNA expression associated with cancer include developing specialist normalisation algorithms and integrating contemporary detection techniques such as miRNA-seq (Liu J. et al., 2018). Large-scale clinical investigations are necessary to validate the therapeutic significance of circulating miRNA biomarkers. This would facilitate the development of customised treatment programmes and the use of focused approaches to the diagnosis and treatment of breast cancer. This comprehensive approach enhances breast cancer therapy by elucidating the complex regulatory networks that govern tumor development and presenting new opportunities for therapeutic strategies that focus on disrupted miRNA-gene interactions.

Furthermore, as of now, there have been several phase 1 and 2 clinical trials conducted on drugs targeting miRNA, but none of these drugs have progressed to a phase III clinical trial. This is partially attributed to the difficulty of developing efficient miRNA carriers and delivery systems that can precisely target certain cell types, regions, and organs. Utilizing antibodies, ligands, and nanoparticles to specifically target miRNAs to a desired cell has been demonstrated to improve the effectiveness of treatment and minimize unintended effects, such as immunotoxicity (Sharma Y. et al., 2022). However, there are still certain limits. Utilizing alternative miRNA carriers, such as AGO proteins or low-density lipoproteins, in conjunction with an appropriate delivery mechanism could potentially augment the cellular absorption of miRNA therapies and their integration into RISC, hence enhancing their regulatory capabilities. Creating approaches to specifically target dysregulated miRNAs in TAMs or CAFs could be beneficial in restoring anti-tumoral characteristics and improving treatment outcomes. The effectiveness and precision of the delivery mechanism are crucial for the application of miRNAs as cancer therapeutic agents. The relevance of this application will be determined after the miRNA carriers successfully transition from phase I/II to phase III. Due to the disruption of various cellular pathways, tumor development occurs. Consequently, conventional therapeutic methods that focus on individual proteins, as well as chemotherapy or radiotherapy alone, have limited efficacy. Utilizing combinatorial strategies involving miRNA therapeutics has demonstrated the ability to decrease chemotherapy resistance and enhance treatment effectiveness in pre-clinical investigations. This paves the way for the development of innovative cancer treatments, pending the resolution of the challenges that have been highlighted.

## Conclusion

Tumor immune evasion is a crucial stage in the malignant advancement of tumors and is a notable contributor to the ineffectiveness of certain tumor therapies. This review primarily examined the impact of miRNA interference on three key areas: innate immune response, specialized immunological response, and apoptotic process. The study has demonstrated that miRNAs are key in both promoting cancerous growth and inhibiting tumor development throughout the process of evading the immune system. At present, an increasing number of nucleic acid therapies are being studied in clinical research. Therefore, it is important to fully utilize the advantages of miRNAs and their involvement in tumor immune evasion, either on their own or as supplementary treatments, due to their potential for development. Nevertheless, because miRNAs are not stable in physiological fluids and there is a need for precise delivery, various biomaterials such as liposomes, viruses, inorganic nanoparticles, and others are used as carriers for delivering therapeutic medicines based on miRNAs. Exosomes, which are naturally occurring substances, can carry nucleic acids and proteins to facilitate communication between cells in both normal and abnormal states.

As miRNA research progresses, an increasing number of studies are investigating the potential of miRNA for diagnosing and predicting prognosis. This includes the development of miRNA-based therapeutics that utilize the expression patterns of certain miRNAs to predict and target immunological escape. miRNAs can also be employed to uncover the pathways that contribute to tumor immune evasion, aiming to comprehend the malignant biological characteristics of tumors.

Although there have been significant advancements in treatment techniques based on miRNAs, the structural characteristics of miRNAs and their interactions with target genes can lead to numerous side effects that are not aligned with the intended therapeutic objectives. Hence, apart from chemically modifying miRNAs, the identification of a biocompatible carrier capable of effectively and safely delivering miRNAs to the designated site is a valuable approach to minimizing adverse effects and off-target effects. Mounting research indicates that exosomes hold great potential as carriers of miRNAs. Nevertheless, other evident concerns an additional verification, including the origin of exosomes, methods of isolation, strategies for loading, and the appropriate selection of medications for loading. Ultimately, the advent of small-molecule RNA therapies is swiftly approaching. We are confident that, as research progresses, miRNA-based therapeutic strategies or interventions will be progressively employed in clinical tumor treatment.

## References

[B1] Abbas M. A. El Sayed I. E. T. Abdu-Allah A. M. K. Kalam A. Al-Sehemi A. G. Al-Hartomy O. A. (2022). Expression of MiRNA-29b and MiRNA-31 and their diagnostic and prognostic values in Egyptian females with breast cancer. Non-Coding RNA Res. 7 (4), 248–257. 10.1016/j.ncrna.2022.09.003 PMC9530401 36247409

[B2] Abplanalp W. T. Fischer A. John D. Zeiher A. M. Gosgnach W. Darville H. (2020). Efficiency and target derepression of anti-miR-92a: results of a first in human study. Nucleic Acid. Ther. 30 (6), 335–345. 10.1089/nat.2020.0871 32707001

[B3] Abtin M. Alivand M. R. Khaniani M. S. Bastami M. Zaeifizadeh M. Derakhshan S. M. (2018). Simultaneous downregulation of miR‐21 and miR‐155 through oleuropein for breast cancer prevention and therapy. J. Cell Biochem. 119 (9), 7151–7165. 10.1002/jcb.26754 29905007

[B4] Acuña S. M. Floeter-Winter L. M. Muxel S. M. (2020). MicroRNAs: biological regulators in pathogen–host interactions. Cells 9 (1), 113. 10.3390/cells9010113 31906500 PMC7016591

[B5] Aggarwal V. Priyanka K. Tuli H. S. (2020). Emergence of circulating MicroRNAs in breast cancer as diagnostic and therapeutic efficacy biomarkers. Mol. Diagn Ther. 24 (2), 153–173. 10.1007/s40291-020-00447-w 32067191

[B6] Ahmed C. S. Winlow P. L. Parsons A. L. Jopling C. L. (2018). Eukaryotic translation initiation factor 4AII contributes to microRNA-122 regulation of hepatitis C virus replication. Nucleic Acids Res. 46 (12), 6330–6343. 10.1093/nar/gky262 29669014 PMC6158612

[B7] Alhasan L. (2019). MiR-126 modulates angiogenesis in breast cancer by targeting VEGF-A-mRNA. Asian Pac J. Cancer Prev. APJCP 20 (1), 193–197. 10.31557/APJCP.2019.20.1.193 30678431 PMC6485552

[B8] Alkabban F. M. Ferguson T. (2023). “Breast cancer,” in StatPearls (Treasure Island (FL): StatPearls Publishing). 29493913

[B9] Almohaywi M. Sugita B. M. Centa A. Fonseca A. S. Antunes V. C. Fadda P. (2023). Deregulated miRNA expression in triple-negative breast cancer of ancestral genomic-characterized Latina patients. Int. J. Mol. Sci. 24 (17), 13046. 10.3390/ijms241713046 37685851 PMC10487916

[B10] Amens J. N. Bahçecioglu G. Zorlutuna P. (2021a). Immune system effects on breast cancer. Cell Mol. Bioeng. 14, 279–292. 10.1007/s12195-021-00679-8 34295441 PMC8280260

[B11] Amens J. N. Bahçecioglu G. Zorlutuna P. (2021b). Immune system effects on breast cancer. Cell Mol. Bioeng. 14 (4), 279–292. 10.1007/s12195-021-00679-8 34295441 PMC8280260

[B12] Anwar S. L. Sari D. N. I. Kartika A. I. Fitria M. S. Tanjung D. S. Rakhmina D. (2019). Upregulation of circulating MiR-21 expression as a potential biomarker for therapeutic monitoring and clinical outcome in breast cancer. Asian Pac J. Cancer Prev. APJCP 20 (4), 1223–1228. 10.31557/APJCP.2019.20.4.1223 31030498 PMC6948877

[B13] Arnold M. Morgan E. Rumgay H. Mafra A. Singh D. Laversanne M. (2022). Current and future burden of breast cancer: global statistics for 2020 and 2040. Breast 66, 15–23. 10.1016/j.breast.2022.08.010 36084384 PMC9465273

[B14] Arun R. P. Cahill H. F. Marcato P. (2022). Breast cancer subtype-specific mirnas: networks, impacts, and the potential for intervention. Biomedicines 10 (3), 651. 10.3390/biomedicines10030651 35327452 PMC8945552

[B15] Asadirad A. Hashemi S. M. Baghaei K. Ghanbarian H. Mortaz E. Zali M. R. (2019). Phenotypical and functional evaluation of dendritic cells after exosomal delivery of miRNA-155. Life Sci. 219, 152–162. 10.1016/j.lfs.2019.01.005 30625290

[B16] Babar I. A. Cheng C. J. Booth C. J. Liang X. Weidhaas J. B. Saltzman W. M. (2012). Nanoparticle-based therapy in an *in vivo* microRNA-155 (miR-155)-dependent mouse model of lymphoma. Proc. Natl. Acad. Sci. 109 (26), E1695–E1704. 10.1073/pnas.1201516109 22685206 PMC3387084

[B17] Bahiraee A. Ebrahimi R. Halabian R. Aghabozorgi A. S. Amani J. (2019). The role of inflammation and its related microRNAs in breast cancer: a narrative review. J. Cell Physiol. 234 (11), 19480–19493. 10.1002/jcp.28742 31025369

[B18] Bahreyni A. Alibolandi M. Ramezani M. Sadeghi A. S. Abnous K. Taghdisi S. M. (2019). A novel MUC1 aptamer-modified PLGA-epirubicin-PβAE-antimir-21 nanocomplex platform for targeted co-delivery of anticancer agents *in vitro* and *in vivo* . Colloids Surf. B Biointerfaces 175, 231–238. 10.1016/j.colsurfb.2018.12.006 30537619

[B19] Bakr N. M. Mahmoud M. S. Nabil R. Boushnak H. Swellam M. (2021). Impact of circulating miRNA-373 on breast cancer diagnosis through targeting VEGF and cyclin D1 genes. J. Genet. Eng. Biotechnol. 19 (1), 84. 10.1186/s43141-021-00174-7 34089425 PMC8179880

[B20] Baldassari F. Zerbinati C. Galasso M. Corrà F. Minotti L. Agnoletto C. (2018). Screen for MicroRNA and drug interactions in breast cancer cell lines points to miR-126 as a modulator of CDK4/6 and PIK3CA inhibitors. Front. Genet. 9, 174. 10.3389/fgene.2018.00174 29868122 PMC5968201

[B21] Bashir S. Loya A. Tabish S. Mushtaq S. Hassan U. Hussain M. (2021). Expression of B-cell lymphoma 2 in breast cancer. J. Cancer Allied Spec. 7 (1), e369. 10.37029/jcas.v7i1.369 37197402 PMC10166318

[B22] Bautista-Sánchez D. Arriaga-Canon C. Pedroza-Torres A. De La Rosa-Velázquez I. A. González-Barrios R. Contreras-Espinosa L. (2020b). The promising role of miR-21 as a cancer biomarker and its importance in RNA-based therapeutics. Mol. Ther-Nucleic Acids 20, 409–420. 10.1016/j.omtn.2020.03.003 32244168 PMC7118281

[B23] Bautista-Sánchez D. Arriaga-Canon C. Pedroza-Torres A. Rosa-Velázquez IADL González-Barrios R. Contreras-Espinosa L. (2020a). The promising role of miR-21 as a cancer biomarker and its importance in RNA-based therapeutics. Mol. Ther. - Nucleic Acids 20, 409–420. 10.1016/j.omtn.2020.03.003 32244168 PMC7118281

[B24] Bergholtz H. Norum J. H. Sørlie T. (2020). Re-definition of claudin-low as a breast cancer phenotype. Nat. Commun. 11, 1787. 10.1038/s41467-020-15574-5 32286297 PMC7156396

[B25] Bernstein D. L. Jiang X. Rom S. (2021). let-7 microRNAs: their role in cerebral and cardiovascular diseases, inflammation, cancer, and their regulation. Biomedicines 9 (6), 606. 10.3390/biomedicines9060606 34073513 PMC8227213

[B26] Bhattacharyya G. S. Doval D. C. Desai C. J. Chaturvedi H. Sharma S. Somashekhar S. P. (2020). Overview of breast cancer and implications of overtreatment of early-stage breast cancer: an Indian perspective. JCO Glob. Oncol. 6, 789–798. 10.1200/GO.20.00033 32511068 PMC7328098

[B27] Boivin V. Deschamps-Francoeur G. Scott M. S. (2018). Protein coding genes as hosts for noncoding RNA expression. Semin. Cell Dev. Biol. 75, 3–12. 10.1016/j.semcdb.2017.08.016 28811264

[B28] Breunig C. Pahl J. Kueblbeck M. Miller M. Antonelli D. Erdem N. (2017). MicroRNA-519a-3p mediates apoptosis resistance in breast cancer cells and their escape from recognition by natural killer cells. Cell Death Dis. 8 (8), e2973. 10.1038/cddis.2017.364 28771222 PMC5596553

[B29] Cai J. Guan H. Fang L. Yang Y. Zhu X. Yuan J. (2013). MicroRNA-374a activates Wnt/β-catenin signaling to promote breast cancer metastasis. J. Clin. Invest. 123 (2), 566–579. 10.1172/JCI65871 23321667 PMC3561816

[B30] Cai W. L. Huang W. D. Li B. Chen T. R. Li Z. X. Zhao C. L. (2018). microRNA-124 inhibits bone metastasis of breast cancer by repressing Interleukin-11. Mol. Cancer 17 (1), 9–14. 10.1186/s12943-017-0746-0 29343249 PMC5773190

[B31] Camps C. Saini H. K. Mole D. R. Choudhry H. Reczko M. Guerra-Assunção J. A. (2014). Integrated analysis of microRNA and mRNA expression and association with HIF binding reveals the complexity of microRNA expression regulation under hypoxia. Mol. Cancer 13, 28–21. 10.1186/1476-4598-13-28 24517586 PMC3928101

[B32] Cao L. Niu Y. (2020). Triple negative breast cancer: special histological types and emerging therapeutic methods. Cancer Biol. Med. 17 (2), 293–306. 10.20892/j.issn.2095-3941.2019.0465 32587770 PMC7309458

[B33] Cao W. Chen H. D. Yu Y. W. Li N. Chen W. Q. (2021). Changing profiles of cancer burden worldwide and in China: a secondary analysis of the global cancer statistics 2020. Chin. Med. J. Engl. 134 (07), 783–791. 10.1097/CM9.0000000000001474 33734139 PMC8104205

[B34] Cavallari I. Ciccarese F. Sharova E. Urso L. Raimondi V. Silic-Benussi M. (2021). The miR-200 family of microRNAs: fine tuners of epithelial-mesenchymal transition and circulating cancer biomarkers. Cancers 13 (23), 5874. 10.3390/cancers13235874 34884985 PMC8656820

[B35] Centers for Disease Control and Prevention (2023). What are the risk factors for breast cancer? Available at: https://www.cdc.gov/cancer/breast/basic_info/risk_factors.htm (Accessed November 28, 2023).

[B36] Chakrabortty A. Patton D. J. Smith B. F. Agarwal P. (2023). miRNAs: potential as biomarkers and therapeutic targets for cancer. Genes (Basel) 14, 1375. 10.3390/genes14071375 37510280 PMC10378777

[B37] Chen J. Sun J. Wang Q. Du Y. Cheng J. Yi J. (2022). Systemic deficiency of PTEN accelerates breast cancer growth and metastasis. Front. Oncol. 12, 825484. 10.3389/fonc.2022.825484 35372075 PMC8971716

[B38] Chen L. Gao D. Shao Z. Zheng Q. Yu Q. (2020). miR-155 indicates the fate of CD4+ T cells. Immunol. Lett. 224, 40–49. 10.1016/j.imlet.2020.05.003 32485191

[B39] Chen L. liang Zhang Z. jing Yi Z. bo Li J. jun (2017b). MicroRNA-211-5p suppresses tumour cell proliferation, invasion, migration and metastasis in triple-negative breast cancer by directly targeting SETBP1. Br. J. Cancer 117 (1), 78–88. 10.1038/bjc.2017.150 28571042 PMC5520212

[B40] Chen P. Xu W. Luo Y. Zhang Y. He Y. Yang S. (2017a). MicroRNA 543 suppresses breast cancer cell proliferation, blocks cell cycle and induces cell apoptosis via direct targeting of ERK/MAPK. OncoTargets Ther. 10, 1423–1431. 10.2147/OTT.S118366 PMC5348068 28331335

[B41] Chen W. Hoffmann A. D. Liu H. Liu X. (2018). Organotropism: new insights into molecular mechanisms of breast cancer metastasis. NPJ Precis. Oncol. 2 (1), 4. 10.1038/s41698-018-0047-0 29872722 PMC5871901

[B42] Cheng Y. Li Y. Nian Y. Liu D. Dai F. Zhang J. (2015). STAT3 is involved in miR-124-mediated suppressive effects on esophageal cancer cells. BMC Cancer 15, 306–311. 10.1186/s12885-015-1303-0 25928665 PMC4479077

[B43] Chu J. Zhu Y. Liu Y. Sun L. Lv X. Wu Y. (2015). E2F7 overexpression leads to tamoxifen resistance in breast cancer cells by competing with E2F1 at miR-15a/16 promoter. Oncotarget 6 (31), 31944–31957. 10.18632/oncotarget.5128 26397135 PMC4741652

[B44] Citron F. Armenia J. Franchin G. Polesel J. Talamini R. D’Andrea S. (2017). An integrated approach identifies mediators of local recurrence in head and neck squamous carcinoma. Clin. Cancer Res. 23 (14), 3769–3780. 10.1158/1078-0432.CCR-16-2814 28174235 PMC7309652

[B45] Conant E. F. Soo M. S. (2021). Molecular subtypes of breast cancer: a review for breast radiologists. J. Breast Imaging 3, 12–24. 10.1093/jbi/wbaa110 38424845

[B46] Cortez M. A. Anfossi S. Ramapriyan R. Menon H. Atalar S. C. Aliru M. (2019). Role of miRNAs in immune responses and immunotherapy in cancer. Genes Chromosom. Cancer 58 (4), 244–253. 10.1002/gcc.22725 30578699 PMC6368474

[B47] Costales M. G. Haga C. L. Velagapudi S. P. Childs-Disney J. L. Phinney D. G. Disney M. D. (2017). Small molecule inhibition of microRNA-210 reprograms an oncogenic hypoxic circuit. J. Am. Chem. Soc. 139 (9), 3446–3455. 10.1021/jacs.6b11273 28240549 PMC5810126

[B48] Creugny A. Fender A. Pfeffer S. (2018). Regulation of primary micro RNA processing. FEBS Lett. 592 (12), 1980–1996. 10.1002/1873-3468.13067 29683487

[B49] Cristofoletti C. Bresin A. Picozza M. Picchio M. C. Monzo F. Helmer C. M. (2019). Blood and skin-derived Sezary cells: differences in proliferation-index, activation of PI3K/AKT/mTORC1 pathway and its prognostic relevance. Leukemia 33 (5), 1231–1242. 10.1038/s41375-018-0305-8 30518812 PMC6756225

[B50] Cui X. Sun Y. Shen M. Song K. Yin X. Di W. (2018). Enhanced chemotherapeutic efficacy of paclitaxel nanoparticles co-delivered with microRNA-7 by inhibiting paclitaxel-induced EGFR/ERK pathway activation for ovarian cancer therapy. ACS Appl. Mater Interfaces 10 (9), 7821–7831. 10.1021/acsami.7b19183 29411964

[B51] Dan T. Shastri A. A. Palagani A. Buraschi S. Neill T. Savage J. E. (2021). miR-21 plays a dual role in tumor formation and cytotoxic response in breast tumors. Cancers 13 (4), 888. 10.3390/cancers13040888 33672628 PMC7924198

[B52] Daugaard I. Hansen T. B. (2017). Biogenesis and function of ago-associated RNAs. Trends Genet. 33 (3), 208–219. 10.1016/j.tig.2017.01.003 28174021

[B53] De Cicco P. Catani M. V. Gasperi V. Sibilano M. Quaglietta M. Savini I. (2019). Nutrition and breast cancer: a literature review on prevention, treatment and recurrence. Nutrients 11 (7), 1514. 10.3390/nu11071514 31277273 PMC6682953

[B54] Dekkers I. A. Cleven A. Lamb H. J. Kroon H. M. (2019). Primary osteosarcoma of the breast. RadioGraphics 39 (3), 626–629. 10.1148/rg.2019180181 31059400

[B55] Deng X. Cao M. Zhang J. Hu K. Yin Z. Zhou Z. (2014). Hyaluronic acid-chitosan nanoparticles for co-delivery of MiR-34a and doxorubicin in therapy against triple negative breast cancer. Biomaterials 35 (14), 4333–4344. 10.1016/j.biomaterials.2014.02.006 24565525

[B56] Devulapally R. Sekar T. V. Paulmurugan R. (2015). Formulation of anti-miR-21 and 4-hydroxytamoxifen co-loaded biodegradable polymer nanoparticles and their antiproliferative effect on breast cancer cells. Mol. Pharm. 12 (6), 2080–2092. 10.1021/mp500852s 25880495 PMC4687493

[B57] Diener C. Keller A. Meese E. (2022). Emerging concepts of miRNA therapeutics: from cells to clinic. Trends Genet. 38 (6), 613–626. 10.1016/j.tig.2022.02.006 35303998

[B58] Dinami R. Ercolani C. Petti E. Piazza S. Ciani Y. Sestito R. (2014). miR-155 drives telomere fragility in human breast cancer by targeting TRF1. Cancer Res. 74 (15), 4145–4156. 10.1158/0008-5472.CAN-13-2038 24876105

[B59] Dong M. Xie Y. Xu Y. (2019). miR-7-5p regulates the proliferation and migration of colorectal cancer cells by negatively regulating the expression of Krüppel-like factor 4. Oncol. Lett. 17 (3), 3241–3246. 10.3892/ol.2019.10001 30867755 PMC6396112

[B60] Dong Y. Liu Y. Jiang A. Li R. Yin M. Wang Y. (2018). MicroRNA-335 suppresses the proliferation, migration, and invasion of breast cancer cells by targeting EphA4. Mol. Cell Biochem. 439, 95–104. 10.1007/s11010-017-3139-1 28795314

[B61] Elia I. Haigis M. C. (2021). Metabolites and the tumour microenvironment: from cellular mechanisms to systemic metabolism. Nat. Metab. 3 (1), 21–32. 10.1038/s42255-020-00317-z 33398194 PMC8097259

[B62] Fan L. Yang Q. Tan J. Qiao Y. Wang Q. He J. (2015). Dual loading miR-218 mimics and Temozolomide using AuCOOH@FA-CS drug delivery system: promising targeted anti-tumor drug delivery system with sequential release functions. J. Exp. Clin. Cancer Res. 34, 106–109. 10.1186/s13046-015-0216-8 26407971 PMC4582616

[B63] Fernández-Hernando C. Suárez Y. (2018). MicroRNAs in endothelial cell homeostasis and vascular disease. Curr. Opin. Hematol. 25 (3), 227–236. 10.1097/MOH.0000000000000424 29547400 PMC6175704

[B64] Fong M. Y. Zhou W. Liu L. Alontaga A. Y. Chandra M. Ashby J. (2015). Breast-cancer-secreted miR-122 reprograms glucose metabolism in premetastatic niche to promote metastasis. Nat. Cell Biol. 17 (2), 183–194. 10.1038/ncb3094 25621950 PMC4380143

[B65] Forterre A. Komuro H. Aminova S. Harada M. (2020). A comprehensive review of cancer MicroRNA therapeutic delivery strategies. Cancers 12 (7), 1852. 10.3390/cancers12071852 32660045 PMC7408939

[B66] Foss F. M. Querfeld C. Kim Y. H. Pinter-Brown L. C. William B. M. Porcu P. (2018). Ph 1 study of MRG-106, an inhibitor of miR-155, in CTCL. J. Clin. Oncol. 36 (15_Suppl. l), 2511. 10.1200/JCO.2018.36.15_suppl.2511

[B67] Frédérick P. Simard M. J. (2022). Regulation and different functions of the animal microRNA‐induced silencing complex. Wiley Interdiscip. Rev. RNA 13 (4), e1701. 10.1002/wrna.1701 34725940

[B68] Fu L. Q. Du W. L. Cai M. H. Yao J. Y. Zhao Y. Y. Mou X. Z. (2020). The roles of tumor-associated macrophages in tumor angiogenesis and metastasis. Cell Immunol. 353, 104119. 10.1016/j.cellimm.2020.104119 32446032

[B69] Gao N. Dong L. (2017). MicroRNA-146 regulates the inflammatory cytokines expression in vascular endothelial cells during sepsis. Pharm- Int. J. Pharm. Sci. 72 (11), 700–704. 10.1691/ph.2017.7600 29442046

[B70] Gebert L. F. Rebhan M. A. Crivelli S. E. Denzler R. Stoffel M. Hall J. (2014). Miravirsen (SPC3649) can inhibit the biogenesis of miR-122. Nucleic Acids Res. 42 (1), 609–621. 10.1093/nar/gkt852 24068553 PMC3874169

[B71] Ghafouri-Fard S. Khanbabapour Sasi A. Abak A. Shoorei H. Khoshkar A. Taheri M. (2021). Contribution of miRNAs in the pathogenesis of breast cancer. Front. Oncol. 11, 768949. 10.3389/fonc.2021.768949 34804971 PMC8602198

[B72] Gong Y. Fan Z. Luo G. Yang C. Huang Q. Fan K. (2019). The role of necroptosis in cancer biology and therapy. Mol. Cancer 18 (1), 100. 10.1186/s12943-019-1029-8 31122251 PMC6532150

[B73] Goropevšek A. Holcar M. Avčin T. (2017). The role of STAT signaling pathways in the pathogenesis of systemic lupus erythematosus. Clin. Rev. Allergy Immunol. 52 (2), 164–181. 10.1007/s12016-016-8550-y 27216430

[B74] Grimaldi A. M. Nuzzo S. Condorelli G. Salvatore M. Incoronato M. (2020). Prognostic and clinicopathological significance of MiR-155 in breast cancer: a systematic review. Int. J. Mol. Sci. 21 (16), 5834. 10.3390/ijms21165834 32823863 PMC7461504

[B75] Grimaldi A. M. Salvatore M. Incoronato M. (2021). miRNA-based therapeutics in breast cancer: a systematic review. Front. Oncol. 11, 668464. 10.3389/fonc.2021.668464 34026646 PMC8131824

[B76] Guo X. Connick M. C. Vanderhoof J. Ishak M. A. Hartley R. S. (2015). MicroRNA-16 modulates HuR regulation of cyclin E1 in breast cancer cells. Int. J. Mol. Sci. 16 (4), 7112–7132. 10.3390/ijms16047112 25830480 PMC4425007

[B77] Gyamfi J. Lee Y. H. Eom M. Choi J. (2018). Interleukin-6/STAT3 signalling regulates adipocyte induced epithelial-mesenchymal transition in breast cancer cells. Sci. Rep. 8 (1), 8859. 10.1038/s41598-018-27184-9 29891854 PMC5995871

[B78] Hajarnis S. Lakhia R. Patel V. (2015). MicroRNAs and polycystic kidney disease. Brisbane, Australia: Exon Publ, 313–334. 10.15586/codon.pkd.2015.ch13 27512774

[B79] Hamam R. Hamam D. Alsaleh K. A. Kassem M. Zaher W. Alfayez M. (2017). Circulating microRNAs in breast cancer: novel diagnostic and prognostic biomarkers. Cell Death Dis. 8 (9), e3045. 10.1038/cddis.2017.440 28880270 PMC5636984

[B80] Hannafon B. N. Trigoso Y. D. Calloway C. L. Zhao Y. D. Lum D. H. Welm A. L. (2016). Plasma exosome microRNAs are indicative of breast cancer. Breast Cancer Res. 18, 90–14. 10.1186/s13058-016-0753-x 27608715 PMC5016889

[B81] Hardeman E. C. Bryce N. S. Gunning P. W. (2020). Impact of the actin cytoskeleton on cell development and function mediated via tropomyosin isoforms. Semin. Cell Dev. Biol. 102, 122–131. 10.1016/j.semcdb.2019.10.004 31630997

[B82] Hashemi M. Mirdamadi M. S. A. Talebi Y. Khaniabad N. Banaei G. Daneii P. (2023). Pre-clinical and clinical importance of miR-21 in human cancers: tumorigenesis, therapy response, delivery approaches and targeting agents. Pharmacol. Res. 187, 106568. 10.1016/j.phrs.2022.106568 36423787

[B83] Hashmi A. A. Hashmi K. A. Irfan M. Khan S. M. Edhi M. M. Ali J. P. (2019). Ki67 index in intrinsic breast cancer subtypes and its association with prognostic parameters. BMC Res. Notes 12, 605–5. 10.1186/s13104-019-4653-x 31547858 PMC6755684

[B84] Hill M. Tran N. (2021). miRNA interplay: mechanisms and consequences in cancer. Dis. Model Mech. 14 (4), dmm047662. 10.1242/dmm.047662 33973623 PMC8077553

[B85] Hong B. S. Ryu H. S. Kim N. Kim J. Lee E. Moon H. (2019). Tumor suppressor miRNA-204-5p regulates growth, metastasis, and immune microenvironment remodeling in breast cancer. Cancer Res. 79 (7), 1520–1534. 10.1158/0008-5472.CAN-18-0891 30737233

[B86] Hong H. Yu H. Yuan J. Guo C. Cao H. Li W. (2016). MicroRNA-200b impacts breast cancer cell migration and invasion by regulating Ezrin-Radixin-Moesin. Med. Sci. Monit. Int. Med. J. Exp. Clin. Res. 22, 1946–1952. 10.12659/msm.896551 PMC4917322 27276064

[B87] Hoorzad P. Mousavinasab F. Tofigh P. Kalahroud E. M. Aghaei-Zarch S. M. Salehi A. (2023). Understanding the lncRNA/miRNA-NFκB regulatory network in diabetes mellitus: from function to clinical translation. Diabetes Res. Clin. Pract. 202, 110804. 10.1016/j.diabres.2023.110804 37369279

[B88] Hua K. Jin J. Zhao J. Song J. Song H. Li D. (2016). miR-135b, upregulated in breast cancer, promotes cell growth and disrupts the cell cycle by regulating LATS2. Int. J. Oncol. 48 (5), 1997–2006. 10.3892/ijo.2016.3405 26934863

[B89] Huang F. Shi Q. Li Y. Xu L. Xu C. Chen F. (2018a). HER2/EGFR–AKT signaling switches TGFβ from inhibiting cell proliferation to promoting cell migration in breast cancer. Cancer Res. 78 (21), 6073–6085. 10.1158/0008-5472.CAN-18-0136 30171053

[B90] Huang S. Fan W. Wang L. Liu H. Wang X. Zhao H. (2020). Maspin inhibits MCF-7 cell invasion and proliferation by downregulating miR-21 and increasing the expression of its target genes. Oncol. Lett. 19 (4), 2621–2628. 10.3892/ol.2020.11360 32218812 PMC7068223

[B91] Huang S. Liu Y. Xu X. Ji M. Li Y. Song C. (2018b). Triple therapy of hepatocellular carcinoma with microRNA-122 and doxorubicin co-loaded functionalized gold nanocages. J. Mat. Chem. B 6, 2217–2229. 10.1039/c8tb00224j 32254562

[B92] Huang X. Lyu J. (2018). Tumor suppressor function of miR-483-3p on breast cancer via targeting of the cyclin E1 gene. Exp. Ther. Med. 16 (3), 2615–2620. 10.3892/etm.2018.6504 30186493 PMC6122499

[B93] Huang Z. Ge H. Yang C. Cai Y. Chen Z. Tian W. (2019). MicroRNA‐26a‐5p inhibits breast cancer cell growth by suppressing RNF6 expression. Kaohsiung J. Med. Sci. 35 (8), 467–473. 10.1002/kjm2.12085 31063232 PMC11900708

[B94] Hussen B. M. Abdullah S. T. Rasul M. F. Salihi A. Ghafouri-Fard S. Hidayat H. J. (2021). MicroRNAs: important players in breast cancer angiogenesis and therapeutic targets. Front. Mol. Biosci. 8, 764025. 10.3389/fmolb.2021.764025 34778378 PMC8582349

[B95] Ilhan A. Golestani S. Shafagh S. G. Asadi F. Daneshdoust D. Al-Naqeeb B. Z. T. (2023). The dual role of microRNA (miR)-20b in cancers: friend or foe? Cell Commun. Signal 21 (1), 26–13. 10.1186/s12964-022-01019-7 36717861 PMC9885628

[B96] Iorio M. V. Casalini P. Piovan C. Braccioli L. Tagliabue E. (2011). Breast cancer and microRNAs: therapeutic impact. Breast 20, S63–S70. 10.1016/S0960-9776(11)70297-1 22015296

[B97] Jang J. H. Lee T. J. (2021). The role of microRNAs in cell death pathways. Yeungnam Univ. J. Med. 38 (2), 107–117. 10.12701/yujm.2020.00836 33435638 PMC8016624

[B98] Janssen H. L. Reesink H. W. Lawitz E. J. Zeuzem S. Rodriguez-Torres M. Patel K. (2013). Treatment of HCV infection by targeting microRNA. N. Engl. J. Med. 368 (18), 1685–1694. 10.1056/NEJMoa1209026 23534542

[B99] Jia S. dong M. W. yan Z. C. Zhang Y. Yao Z. H. hui Q. X. (2019). Tanshinone IIA attenuates high glucose induced human VSMC proliferation and migration through miR-21-5p-mediated tropomyosin 1 downregulation. Arch. Biochem. Biophys. 677, 108154. 10.1016/j.abb.2019.108154 31672498

[B100] Jiang Q. He M. Ma M. T. Wu H. Z. Yu Z. J. Guan S. (2016). MicroRNA-148a inhibits breast cancer migration and invasion by directly targeting WNT-1. Oncol. Rep. 35 (3), 1425–1432. 10.3892/or.2015.4502 26707142

[B101] Jin T. Kim H. S. Choi S. K. Hwang E. H. Woo J. Ryu H. S. (2017). microRNA-200c/141 upregulates SerpinB2 to promote breast cancer cell metastasis and reduce patient survival. Oncotarget 8 (20), 32769–32782. 10.18632/oncotarget.15680 28427146 PMC5464826

[B102] Jo H. Shim K. Jeoung D. (2022). Potential of the miR-200 family as a target for developing anti-cancer therapeutics. Int. J. Mol. Sci. 23 (11), 5881. 10.3390/ijms23115881 35682560 PMC9180509

[B103] Jordan-Alejandre E. Campos-Parra A. D. Castro-López D. L. Silva-Cázares M. B. (2023). Potential miRNA use as a biomarker: from breast cancer diagnosis to metastasis. Cells 12 (4), 525. 10.3390/cells12040525 36831192 PMC9954167

[B104] Juang V. Chang C.-H. Wang C.-S. Wang H.-E. Lo Y.-L. (2019). pH-Responsive PEG-shedding and targeting peptide-modified nanoparticles for dual-delivery of irinotecan and microRNA to enhance tumor-specific therapy. Small 15, e1903296. 10.1002/smll.201903296 31709707

[B105] Kahraman M. Röske A. Laufer T. Fehlmann T. Backes C. Kern F. (2018). MicroRNA in diagnosis and therapy monitoring of early-stage triple-negative breast cancer. Sci. Rep. 8 (1), 11584. 10.1038/s41598-018-29917-2 30072748 PMC6072710

[B106] Kalkusova K. Taborska P. Stakheev D. Smrz D. (2022). The role of miR-155 in antitumor immunity. Cancers 14 (21), 5414. 10.3390/cancers14215414 36358832 PMC9659277

[B107] Kamali M. J. Salehi M. Fatemi S. Moradi F. Khoshghiafeh A. Ahmadifard M. (2022). Locked nucleic acid (LNA): a modern approach to cancer diagnosis and treatment. Exp. Cell Res. 423, 113442. 10.1016/j.yexcr.2022.113442 36521777

[B108] Karami F. M. Azargoonjahromi A. Kiani A. Jalalifar F. Osati P. Akbari O. M. (2022a). The role of epigenetic modifications in drug resistance and treatment of breast cancer. Cell Mol. Biol. Lett. 27 (1), 1–25. 10.1186/s11658-022-00344-6 35764927 PMC9238060

[B109] Karami F. M. Azargoonjahromi A. Kiani A. Jalalifar F. Osati P. Akbari O. M. (2022b). The role of epigenetic modifications in drug resistance and treatment of breast cancer. Cell Mol. Biol. Lett. 27 (1), 52. 10.1186/s11658-022-00344-6 35764927 PMC9238060

[B110] Kashyap D. Pal D. Sharma R. Garg V. K. Goel N. Koundal D. (2022). Global increase in breast cancer incidence: risk factors and preventive measures. Biomed. Res. Int. 2022, e9605439. 10.1155/2022/9605439 PMC9038417 35480139

[B111] Khalid N. Azimpouran M. (2023). “Necrosis,” in StatPearls (Treasure Island (FL): StatPearls Publishing). 32491559

[B112] Kim J. (2021). Identification of MicroRNAs as diagnostic biomarkers for breast cancer based on the cancer genome atlas. Diagnostics 11 (1), 107. 10.3390/diagnostics11010107 33440868 PMC7827427

[B113] Kong W. He L. Richards E. Challa S. Xu C. Permuth-Wey J. (2014). Upregulation of miRNA-155 promotes tumour angiogenesis by targeting VHL and is associated with poor prognosis and triple-negative breast cancer. Oncogene 33 (6), 679–689. 10.1038/onc.2012.636 23353819 PMC3925335

[B114] Kontomanolis E. N. Kalagasidou S. Pouliliou S. Anthoulaki X. Georgiou N. Papamanolis V. (2018). The Notch pathway in breast cancer progression. Sci. World J. 2018, e2415489. 10.1155/2018/2415489 PMC6077551 30111989

[B115] Küçüktürkmen B. Bozkır A. (2018). Development and characterization of cationic solid lipid nanoparticles for co-delivery of pemetrexed and miR-21 antisense oligonucleotide to glioblastoma cells. Drug Dev. Ind. Pharm. 44 (2), 306–315. 10.1080/03639045.2017.1391835 29023168

[B116] Küçüktürkmen B. Devrim B. Saka O. M. Yilmaz Ş. Arsoy T. Bozkir A. (2017). Co-delivery of pemetrexed and miR-21 antisense oligonucleotide by lipid-polymer hybrid nanoparticles and effects on glioblastoma cells. Drug Dev. Ind. Pharm. 43 (1), 12–21. 10.1080/03639045.2016.1200069 27277750

[B117] Larsen M. J. Kruse T. A. Tan Q. Laenkholm A. V. Bak M. Lykkesfeldt A. E. (2013). Classifications within molecular subtypes enables identification of BRCA1/BRCA2 mutation carriers by RNA tumor profiling. PloS One 8 (5), e64268. 10.1371/journal.pone.0064268 23704984 PMC3660328

[B118] Law A. M. K. Valdes-Mora F. Gallego-Ortega D. (2020). Myeloid-derived suppressor cells as a therapeutic target for cancer. Cells 9 (3), 561. 10.3390/cells9030561 32121014 PMC7140518

[B119] Lee M. S. Azmiyaty Amar Ma’ ruf C. Nadhirah Izhar D. P. Nafisah I. S. Wan Jamaluddin W. S. Ya’acob S. N. M. (2019). Awareness on breast cancer screening in Malaysia: a cross sectional study. BioMedicine 9 (3), 18. 10.1051/bmdcn/2019090318 31453799 PMC6711317

[B120] Li B. Wang X. Choi I. Y. Wang Y. C. Liu S. Pham A. T. (2017a). miR-146a modulates autoreactive Th17 cell differentiation and regulates organ-specific autoimmunity. J. Clin. Invest. 127 (10), 3702–3716. 10.1172/JCI94012 28872459 PMC5617680

[B121] Li F. Sun H. Yu Y. Che N. Han J. Cheng R. (2023). RIPK1-dependent necroptosis promotes vasculogenic mimicry formation via eIF4E in triple-negative breast cancer. Cell Death Dis. 14, 335. 10.1038/s41419-023-05841-w 37217473 PMC10203343

[B122] Li G. Yao L. Zhang J. Li X. Dang S. Zeng K. (2016b). Tumor-suppressive microRNA-34a inhibits breast cancer cell migration and invasion via targeting oncogenic TPD52. Tumor Biol. 37, 7481–7491. 10.1007/s13277-015-4623-4 26678891

[B123] Li M. Goldman D. P. Chen A. J. (2021a). Spending on Targeted Therapies Reduced Mortality in Patients with Advanced-Stage Breast Cancer: study examines spending on targeted therapies for patients with advanced-stage breast cancer and the impact on mortality. Health Aff. (Millwood) 40 (5), 763–771. 10.1377/hlthaff.2020.01714 33939503

[B124] Li M. Li Q. Yin Q. Wang Y. Shang J. Wang L. (2021b). Evaluation of color Doppler ultrasound combined with plasma miR-21 and miR-27a in the diagnosis of breast cancer. Clin. Transl. Oncol. 23, 709–717. 10.1007/s12094-020-02501-9 33206330

[B125] Li M. Su Y. Zhang F. Chen K. Xu X. Xu L. (2018). A dual-targeting reconstituted high density lipoprotein leveraging the synergy of sorafenib and antimiRNA21 for enhanced hepatocellular carcinoma therapy. Acta Biomater. 75, 413–426. 10.1016/j.actbio.2018.05.049 29859368

[B126] Li M. Zou X. Xia T. Wang T. Liu P. Zhou X. (2019a). A five‐miRNA panel in plasma was identified for breast cancer diagnosis. Cancer Med. 8 (16), 7006–7017. 10.1002/cam4.2572 31568692 PMC6853814

[B127] Li P. Guo Y. Bledsoe G. Yang Z. Chao L. Chao J. (2016a). Kallistatin induces breast cancer cell apoptosis and autophagy by modulating Wnt signaling and microRNA synthesis. Exp. Cell Res. 340 (2), 305–314. 10.1016/j.yexcr.2016.01.004 26790955 PMC5082136

[B128] Li P. Zeng Y. Chen Y. Huang P. Chen X. Zheng W. (2022). LRP11-AS1 promotes the proliferation and migration of triple negative breast cancer cells via the miR-149-3p/NRP2 axis. Cancer Cell Int. 22 (1), 116. 10.1186/s12935-022-02536-8 35279146 PMC8917722

[B129] Li Q. Ren P. Shi P. Chen Y. Xiang F. Zhang L. (2017b). MicroRNA-148a promotes apoptosis and suppresses growth of breast cancer cells by targeting B-cell lymphoma 2. Anticancer Drugs 28 (6), 588–595. 10.1097/CAD.0000000000000498 28430743

[B130] Li S. Li D. Cho W. C. Xu S. Yu Z. Shen L. (2020a). Targeted inhibition of miR-221/222 promotes cell sensitivity to cisplatin in triple-negative breast cancer MDA-MB-231 cells. Front. Genet. 10, 455941. 10.3389/fgene.2019.01278 PMC6971202 32010177

[B131] Li X. Wang Q. Rui Y. Zhang C. Wang W. Gu J. (2019b). HOXC13‐AS promotes breast cancer cell growth through regulating miR‐497‐5p/PTEN axis. J. Cell Physiol. 234 (12), 22343–22351. 10.1002/jcp.28800 31066051

[B132] Li Y. Cai B. Shen L. Dong Y. Lu Q. Sun S. (2017c). MiRNA-29b suppresses tumor growth through simultaneously inhibiting angiogenesis and tumorigenesis by targeting Akt3. Cancer Lett. 397, 111–119. 10.1016/j.canlet.2017.03.032 28365400

[B133] Li Y. Chen Y. Li J. Zhang Z. Huang C. Lian G. (2017d). Co‐delivery of micro RNA‐21 antisense oligonucleotides and gemcitabine using nanomedicine for pancreatic cancer therapy. Cancer Sci. 108 (7), 1493–1503. 10.1111/cas.13267 28444967 PMC5497927

[B134] Li Y. Jia F. Deng X. Wang X. Lu J. Shao L. (2020b). Combinatorial miRNA-34a replenishment and irinotecan delivery via auto-fluorescent polymeric hybrid micelles for synchronous colorectal cancer theranostics. Biomater. Sci. 8 (24), 7132–7144. 10.1039/d0bm01579b 33150879

[B135] Liang G. Zhu Y. Ali D. J. Tian T. Xu H. Si K. (2020). Engineered exosomes for targeted co-delivery of miR-21 inhibitor and chemotherapeutics to reverse drug resistance in colon cancer. J. Nanobiotechnology 18, 10–15. 10.1186/s12951-019-0563-2 31918721 PMC6950820

[B136] Lin F. Di W. Wang X. Mahato R. I. (2019). Dual responsive micelles capable of modulating miRNA-34a to combat taxane resistance in prostate cancer. Biomaterials 192, 95–108. 10.1016/j.biomaterials.2018.10.036 30447399

[B137] Lin L. Fan Y. Gao F. Jin L. Li D. Sun W. (2018). UTMD-promoted co-delivery of gemcitabine and miR-21 inhibitor by dendrimer-entrapped gold nanoparticles for pancreatic cancer therapy. Theranostics 8 (7), 1923–1939. 10.7150/thno.22834 29556365 PMC5858509

[B138] Lin S. Gregory R. I. (2015). MicroRNA biogenesis pathways in cancer. Nat. Rev. Cancer 15 (6), 321–333. 10.1038/nrc3932 25998712 PMC4859809

[B139] Lin Y. Liu A. Y. Fan C. Zheng H. Li Y. Zhang C. (2015). MicroRNA-33b inhibits breast cancer metastasis by targeting HMGA2, SALL4 and Twist1. Sci. Rep. 5 (1), 9995. 10.1038/srep09995 25919570 PMC4412117

[B140] Lin Y. X. Wang Y. Blake S. Yu M. Mei L. Wang H. (2020). RNA nanotechnology-mediated cancer immunotherapy. Theranostics 10 (1), 281–299. 10.7150/thno.35568 31903120 PMC6929632

[B141] Liu C. Chen Z. Fang M. Qiao Y. (2019). MicroRNA let-7a inhibits proliferation of breast cancer cell by downregulating USP32 expression. Transl. Cancer Res. 8 (5), 1763–1771. 10.21037/tcr.2019.08.30 35116927 PMC8799222

[B142] Liu G. Chen T. Zhang X. Ma X. Shi H. (2022). Small molecule inhibitors targeting the cancers. MedComm 3 (4), e181. 10.1002/mco2.181 36254250 PMC9560750

[B143] Liu J. Li X. Wang M. Xiao G. Yang G. Wang H. (2018a). A miR-26a/E2F7 feedback loop contributes to tamoxifen resistance in ER-positive breast cancer. Int. J. Oncol. 53 (4), 1601–1612. 10.3892/ijo.2018.4492 30066905

[B144] Liu J. Zhou Y. Shi Z. Hu Y. Meng T. Zhang X. (2016). microRNA-497 modulates breast cancer cell proliferation, invasion, and survival by targeting SMAD7. DNA Cell Biol. 35 (9), 521–529. 10.1089/dna.2016.3282 27303812

[B145] Liu P. Wang S. Liu X. Ding J. Zhou W. (2018b). Platinated graphene oxide: a nanoplatform for efficient gene-chemo combination cancer therapy. Eur. J. Pharm. Sci. 121, 319–329. 10.1016/j.ejps.2018.06.009 29906508

[B146] Liu Q. Li R.-T. Qian H.-Q. Wei J. Xie L. Shen J. (2013). Targeted delivery of miR-200c/DOC to inhibit cancer stem cells and cancer cells by the gelatinases-stimuli nanoparticles. Biomaterials 34, 7191–7203. 10.1016/j.biomaterials.2013.06.004 23806972

[B147] Loh H. Y. Norman B. P. Lai K. S. Rahman N. Alitheen N. B. M. Osman M. A. (2019). The regulatory role of MicroRNAs in breast cancer. Int. J. Mol. Sci. 20 (19), 4940. 10.3390/ijms20194940 31590453 PMC6801796

[B148] Loric S. Denis J. A. Desbene C. Sabbah M. Conti M. (2023). Extracellular vesicles in breast cancer: from biology and function to clinical diagnosis and therapeutic management. Int. J. Mol. Sci. 24 (8), 7208. 10.3390/ijms24087208 37108371 PMC10139222

[B149] Lu Y. Qin T. Li J. Wang L. Zhang Q. Jiang Z. (2017). MicroRNA-140-5p inhibits invasion and angiogenesis through targeting VEGF-A in breast cancer. Cancer Gene Ther. 24 (9), 386–392. 10.1038/cgt.2017.30 28752859 PMC5668497

[B150] Łukasiewicz S. Czeczelewski M. Forma A. Baj J. Sitarz R. Stanisławek A. (2021a). Breast cancer—epidemiology, risk factors, classification, prognostic markers, and current treatment strategies—an updated review. Cancers 13 (17), 4287. 10.3390/cancers13174287 34503097 PMC8428369

[B151] Łukasiewicz S. Czeczelewski M. Forma A. Baj J. Sitarz R. Stanisławek A. (2021b). Breast cancer—epidemiology, risk factors, classification, prognostic markers, and current treatment strategies—an updated review. Cancers (Basel) 13, 4287. 10.3390/cancers13174287 34503097 PMC8428369

[B152] Luo Q. Li X. Gao Y. Long Y. Chen L. Huang Y. (2013). MiRNA-497 regulates cell growth and invasion by targeting cyclin E1 in breast cancer. Cancer Cell Int. 13, 95–98. 10.1186/1475-2867-13-95 24112607 PMC3853026

[B153] Luo T. Liu Q. Tan A. Duan L. Jia Y. Nong L. (2020). Mesenchymal stem cell-secreted exosome promotes chemoresistance in breast cancer via enhancing miR-21-5p-mediated S100A6 expression. Mol. Ther-Oncolytics 19, 283–293. 10.1016/j.omto.2020.10.008 33294586 PMC7689030

[B154] Lv P. Zhang Z. Hou L. Zhang Y. Lu L. Wang C. (2020). Meta-analysis of the clinicopathological significance of miRNA-145 in breast cancer. Biosci. Rep. 40 (9), BSR20193974. 10.1042/BSR20193974 32869851 PMC7502658

[B155] Ma Y. Shen N. Wicha M. S. Luo M. (2021). The roles of the Let-7 family of MicroRNAs in the regulation of cancer stemness. Cells 10 (9), 2415. 10.3390/cells10092415 34572067 PMC8469079

[B156] Magalhães M. Jorge J. Gonçalves A. C. Sarmento-Ribeiro A. B. Carvalho R. Figueiras A. (2020). miR-29b and retinoic acid co-delivery: a promising tool to induce a synergistic antitumoral effect in non-small cell lung cancer cells. Drug Deliv. Transl. Res. 10, 1367–1380. 10.1007/s13346-020-00768-7 32358723

[B157] Maldonado E. Morales-Pison S. Urbina F. Jara L. Solari A. (2022). Role of the mediator complex and MicroRNAs in breast cancer etiology. Genes (Basel) 13, 234. 10.3390/genes13020234 35205279 PMC8871970

[B158] Mammes A. Pasquier J. Mammes O. Conti M. Douard R. Loric S. (2021). Extracellular vesicles: general features and usefulness in diagnosis and therapeutic management of colorectal cancer. World J. Gastrointest. Oncol. 13 (11), 1561–1598. 10.4251/wjgo.v13.i11.1561 34853637 PMC8603448

[B159] Mastroianni J. Stickel N. Andrlova H. Hanke K. Melchinger W. Duquesne S. (2019). miR-146a controls immune response in the melanoma microenvironment. Cancer Res. 79 (1), 183–195. 10.1158/0008-5472.CAN-18-1397 30425059 PMC6330089

[B160] Matsuhashi S. Manirujjaman M. Hamajima H. Ozaki I. (2019). Control mechanisms of the tumor suppressor PDCD4: expression and functions. Int. J. Mol. Sci. 20 (9), 2304. 10.3390/ijms20092304 31075975 PMC6539695

[B161] McAnena P. Tanriverdi K. Curran C. Gilligan K. Freedman J. E. Brown J. A. (2019). Circulating microRNAs miR-331 and miR-195 differentiate local luminal a from metastatic breast cancer. BMC Cancer 19, 436. 10.1186/s12885-019-5636-y 31077182 PMC6511137

[B162] McCarthy A. M. Friebel-Klingner T. Ehsan S. He W. Welch M. Chen J. (2021). Relationship of established risk factors with breast cancer subtypes. Cancer Med. 10 (18), 6456–6467. 10.1002/cam4.4158 34464510 PMC8446564

[B163] McGuire A. Brown J. A. Kerin M. J. (2015). Metastatic breast cancer: the potential of miRNA for diagnosis and treatment monitoring. Cancer Metastasis Rev. 34, 145–155. 10.1007/s10555-015-9551-7 25721950 PMC4368851

[B164] Mehrgou A. Akouchekian M. (2017). Therapeutic impacts of microRNAs in breast cancer by their roles in regulating processes involved in this disease. J. Res. Med. Sci. Off. J. Isfahan Univ. Med. Sci. 22, 130. 10.4103/jrms.JRMS_967_16 PMC5767816 29387117

[B165] Miraghel S. A. Ebrahimi N. Khani L. Mansouri A. Jafarzadeh A. Ahmadi A. (2022). Crosstalk between non-coding RNAs expression profile, drug resistance and immune response in breast cancer. Pharmacol. Res. 176, 106041. 10.1016/j.phrs.2021.106041 34952200

[B166] Mittal A. Chitkara D. Behrman S. W. Mahato R. I. (2014). Efficacy of gemcitabine conjugated and miRNA-205 complexed micelles for treatment of advanced pancreatic cancer. Biomaterials 35, 7077–7087. 10.1016/j.biomaterials.2014.04.053 24836307

[B167] Mizuno R. Kawada K. Sakai Y. (2018). The molecular basis and therapeutic potential of *let-7* MicroRNAs against colorectal cancer. Can. J. Gastroenterol. Hepatol. 2018, e5769591. 10.1155/2018/5769591 PMC6029494 30018946

[B168] Mohammadi-Yeganeh S. Paryan M. Arefian E. Vasei M. Ghanbarian H. Mahdian R. (2016). MicroRNA-340 inhibits the migration, invasion, and metastasis of breast cancer cells by targeting Wnt pathway. Tumor Biol. 37, 8993–9000. 10.1007/s13277-015-4513-9 26758430

[B169] Momenimovahed Z. Salehiniya H. (2019). Epidemiological characteristics of and risk factors for breast cancer in the world. Breast Cancer - Targets Ther. 11, 151–164. 10.2147/BCTT.S176070 PMC6462164 31040712

[B170] Moustafa-Kamal M. Kucharski T. J. El-Assaad W. Abbas Y. M. Gandin V. Nagar B. (2020). The mTORC1/S6K/PDCD4/eIF4A axis determines outcome of mitotic arrest. Cell Rep. 33 (1), 108230. 10.1016/j.celrep.2020.108230 33027666

[B171] Mulrane L. McGee S. F. Gallagher W. M. O’Connor D. P. (2013). miRNA dysregulation in breast cancer. Cancer Res. 73 (22), 6554–6562. 10.1158/0008-5472.CAN-13-1841 24204025

[B172] Muraki K. Nyhan K. Han L. Murnane J. P. (2012). Mechanisms of telomere loss and their consequences for chromosome instability. Front. Oncol. 2, 135. 10.3389/fonc.2012.00135 23061048 PMC3463808

[B173] Naeem M. Hayat M. Qamar S. Mehmood T. Munir A. Ahmad G. (2019). Risk factors, genetic mutations and prevention of breast cancer. Int. J. Biosci. 14. 10.12692/ijb/14.4.492-496

[B174] Naeli P. Winter T. Hackett A. P. Alboushi L. Jafarnejad S. M. (2023). The intricate balance between microRNA‐induced mRNA decay and translational repression. FEBS J. 290 (10), 2508–2524. 10.1111/febs.16422 35247033

[B175] Naghizadeh S. Mohammadi A. Duijf P. H. Baradaran B. Safarzadeh E. Cho W. C. (2020). The role of miR‐34 in cancer drug resistance. J. Cell Physiol. 235 (10), 6424–6440. 10.1002/jcp.29640 32064620

[B176] Nagpal N. Ahmad H. M. Chameettachal S. Sundar D. Ghosh S. Kulshreshtha R. (2015). HIF-inducible miR-191 promotes migration in breast cancer through complex regulation of TGFβ-signaling in hypoxic microenvironment. Sci. Rep. 5 (1), 9650. 10.1038/srep09650 25867965 PMC4394754

[B177] Najjary S. Mohammadzadeh R. Mokhtarzadeh A. Mohammadi A. Kojabad A. B. Baradaran B. (2020). Role of miR-21 as an authentic oncogene in mediating drug resistance in breast cancer. Gene 738, 144453. 10.1016/j.gene.2020.144453 32035242

[B178] Nanbo A. Furuyama W. Lin Z. (2021). RNA virus-encoded miRNAs: current insights and future challenges. Front. Microbiol. 12, 679210. 10.3389/fmicb.2021.679210 34248890 PMC8266288

[B179] NCHBI (2020). “How does the immune system work?,” in InformedHealth.org (Cologne, Germany: Institute for Quality and Efficiency in Health Care).

[B180] Ngabire D. Seong Y. A. Patil M. P. Niyonizigiye I. Seo Y. B. Kim G. D. (2018). Induction of apoptosis and G1 phase cell cycle arrest by Aster incisus in AGS gastric adenocarcinoma cells. Int. J. Oncol. 53 (5), 2300–2308. 10.3892/ijo.2018.4547 30226597

[B181] Nguyen T. L. Nguyen T. D. Bao S. Li S. Nguyen T. A. (2020). The internal loops in the lower stem of primary microRNA transcripts facilitate single cleavage of human Microprocessor. Nucleic Acids Res. 48 (5), 2579–2593. 10.1093/nar/gkaa018 31956890 PMC7049713

[B182] Ning Q. Liu Y.-F. Ye P.-J. Gao P. Li Z.-P. Tang S.-Y. (2019). Delivery of liver-specific miRNA-122 using a targeted macromolecular prodrug toward synergistic therapy for hepatocellular carcinoma. ACS Appl. Mat. Interfaces 11, 10578–10588. 10.1021/acsami.9b00634 30802029

[B183] O’Bryan S. Dong S. Mathis J. M. Alahari S. K. (2017). The roles of oncogenic miRNAs and their therapeutic importance in breast cancer. Eur. J. Cancer 72, 1–11. 10.1016/j.ejca.2016.11.004 27997852

[B184] O’Day E. Lal A. (2010). MicroRNAs and their target gene networks in breast cancer. Breast Cancer Res. 12, 201–210. 10.1186/bcr2484 20346098 PMC2879559

[B185] Oliveto S. Mancino M. Manfrini N. Biffo S. (2017). Role of microRNAs in translation regulation and cancer. World J. Biol. Chem. 8 (1), 45–56. 10.4331/wjbc.v8.i1.45 28289518 PMC5329714

[B186] Orangi E. Motovali-Bashi M. (2019). Evaluation of miRNA-9 and miRNA-34a as potential biomarkers for diagnosis of breast cancer in Iranian women. Gene 687, 272–279. 10.1016/j.gene.2018.11.036 30468908

[B187] Otmani K. Lewalle P. (2021). Tumor suppressor miRNA in cancer cells and the tumor microenvironment: mechanism of deregulation and clinical implications. Front. Oncol. 11, 708765. 10.3389/fonc.2021.708765 34722255 PMC8554338

[B188] Pakravan K. Babashah S. Sadeghizadeh M. Mowla S. J. Mossahebi-Mohammadi M. Ataei F. (2017). MicroRNA-100 shuttled by mesenchymal stem cell-derived exosomes suppresses *in vitro* angiogenesis through modulating the mTOR/HIF-1α/VEGF signaling axis in breast cancer cells. Cell Oncol. 40, 457–470. 10.1007/s13402-017-0335-7 PMC13001539 28741069

[B189] Pan G. Liu Y. Shang L. Zhou F. Yang S. (2021). EMT‐associated microRNAs and their roles in cancer stemness and drug resistance. Cancer Commun. 41 (3), 199–217. 10.1002/cac2.12138 PMC7968884 33506604

[B190] Pan Y. Jiao G. Wang C. Yang J. Yang W. (2016). MicroRNA-421 inhibits breast cancer metastasis by targeting metastasis associated 1. Biomed. Pharmacother. 83, 1398–1406. 10.1016/j.biopha.2016.08.058 27583980

[B191] Parida S. Sharma D. (2019). The microbiome–estrogen connection and breast cancer risk. Cells 8, 1642. 10.3390/cells8121642 31847455 PMC6952974

[B192] Passmore L. A. Coller J. (2022). Roles of mRNA poly(A) tails in regulation of eukaryotic gene expression. Nat. Rev. Mol. Cell Biol. 23 (2), 93–106. 10.1038/s41580-021-00417-y 34594027 PMC7614307

[B193] Plichta J. K. Thomas S. M. Vernon R. Fayanju O. M. Rosenberger L. H. Hyslop T. (2020). Breast cancer tumor histopathology, stage at presentation, and treatment in the extremes of age. Breast Cancer Res. Treat. 180 (1), 227–235. 10.1007/s10549-020-05542-4 31980967 PMC7066434

[B194] Pommier R. M. Sanlaville A. Tonon L. Kielbassa J. Thomas E. Ferrari A. (2020). Comprehensive characterization of claudin-low breast tumors reflects the impact of the cell-of-origin on cancer evolution. Nat. Commun. 11 (1), 3431. 10.1038/s41467-020-17249-7 32647202 PMC7347884

[B195] Poojary M. Jishnu P. V. Kabekkodu S. P. (2020). Prognostic value of melanoma-associated antigen-A (MAGE-A) gene expression in various human cancers: a systematic review and meta-analysis of 7428 patients and 44 studies. Mol. Diagn Ther. 24 (5), 537–555. 10.1007/s40291-020-00476-5 32548799 PMC7497308

[B196] Pu M. Chen J. Tao Z. Miao L. Qi X. Wang Y. (2019). Regulatory network of miRNA on its target: coordination between transcriptional and post-transcriptional regulation of gene expression. Cell Mol. Life Sci. CMLS 76 (3), 441–451. 10.1007/s00018-018-2940-7 30374521 PMC11105547

[B197] Purohit P. K. Edwards R. Tokatlidis K. Saini N. (2019). MiR-195 regulates mitochondrial function by targeting mitofusin-2 in breast cancer cells. RNA Biol. 16 (7), 918–929. 10.1080/15476286.2019.1600999 30932749 PMC6546347

[B198] Qattan A. (2020). Novel miRNA targets and therapies in the triple-negative breast cancer microenvironment: an emerging hope for a challenging disease. Int. J. Mol. Sci. 21 (23), 8905. 10.3390/ijms21238905 33255471 PMC7727826

[B199] Qian X. Long L. Shi Z. Liu C. Qiu M. Sheng J. (2014). Star-branched amphiphilic PLA-b-PDMAEMA copolymers for co-delivery of miR-21 inhibitor and doxorubicin to treat glioma. Biomaterials 35 (7), 2322–2335. 10.1016/j.biomaterials.2013.11.039 24332459

[B200] Rani V. Sengar R. S. (2022). Biogenesis and mechanisms of microRNA-mediated gene regulation. Biotechnol. Bioeng. 119 (3), 685–692. 10.1002/bit.28029 34979040

[B201] Rashid K. Ahmad A. Meerasa S. S. Khan A. Q. Wu X. Liang L. (2023). Cancer stem cell-derived exosome-induced metastatic cancer: an orchestra within the tumor microenvironment. Biochimie 212, 1–11. 10.1016/j.biochi.2023.03.014 37011805

[B202] Raue R. Frank A. C. Syed S. N. Brüne B. (2021). Therapeutic targeting of MicroRNAs in the tumor microenvironment. Int. J. Mol. Sci. 22 (4), 2210. 10.3390/ijms22042210 33672261 PMC7926641

[B203] Raval A. Joshi J. Shah F. (2022). Significance of metastamiR-10b in breast cancer therapeutics. J. Egypt Natl. Cancer Inst. 34 (1), 19. 10.1186/s43046-022-00120-9 PMC13314274 35491408

[B204] Reid G. Kao S. C. Pavlakis N. Brahmbhatt H. MacDiarmid J. Clarke S. (2016). Clinical development of TargomiRs, a miRNA mimic-based treatment for patients with recurrent thoracic cancer. Epigenomics 8 (8), 1079–1085. 10.2217/epi-2016-0035 27185582

[B205] Ren Y. Kang C. S. Yuan X. B. Zhou X. Xu P. Han L. (2010). Co-delivery of as-miR-21 and 5-FU by poly (amidoamine) dendrimer attenuates human glioma cell growth *in vitro* . J. Biomater. Sci. Polym. Ed. 21 (3), 303–314. 10.1163/156856209X415828 20178687

[B206] Ren Y. Wang R. Gao L. Li K. Zhou X. Guo H. (2016). Sequential co-delivery of miR-21 inhibitor followed by burst release doxorubicin using NIR-responsive hollow gold nanoparticle to enhance anticancer efficacy. J. Control. Release 228, 74–86. 10.1016/j.jconrel.2016.03.008 26956593

[B207] Ren Y. qiang jun W. H. Zhang Y. qing Liu Y. bing (2017). WBP2 modulates G1/S transition in ER+ breast cancer cells and is a direct target of miR-206. Cancer Chemother. Pharmacol. 79, 1003–1011. 10.1007/s00280-017-3302-0 28391353

[B208] Ro Y. T. Jo G. H. Jung S. A. Lee E. H. Shin J. Lee J. H. (2018). Salmonella-induced miR-155 enhances necroptotic death in macrophage cells via targeting RIP1/3. Mol. Med. Rep. 18 (6), 5133–5140. 10.3892/mmr.2018.9525 30280195

[B209] Rodrigues F. B. Wild E. J. (2020). Huntington’s disease clinical trials corner: april 2020. J. Huntingt Dis. 9 (2), 185–197. 10.3233/JHD-200002 32250312

[B210] Roscigno G. Puoti I. Giordano I. Donnarumma E. Russo V. Affinito A. (2017). MiR-24 induces chemotherapy resistance and hypoxic advantage in breast cancer. Oncotarget 8 (12), 19507–19521. 10.18632/oncotarget.14470 28061479 PMC5386701

[B211] Rui X. Zhao H. Xiao X. Wang L. Mo L. Yao Y. (2018). MicroRNA-34a suppresses breast cancer cell proliferation and invasion by targeting Notch1. Exp. Ther. Med. 16 (6), 4387–4392. 10.3892/etm.2018.6744 30542388 PMC6257824

[B212] Saikia M. Paul S. Chakraborty S. (2020). Role of microRNA in forming breast carcinoma. Life Sci. 259, 118256. 10.1016/j.lfs.2020.118256 32822719

[B213] Sareyeldin R. M. Gupta I. Al-Hashimi I. Al-Thawadi H. A. Al Farsi H. F. Vranic S. (2019). Gene expression and miRNAs profiling: function and regulation in human epidermal growth factor receptor 2 (HER2)-Positive breast cancer. Cancers 11 (5), 646. 10.3390/cancers11050646 31083383 PMC6562440

[B214] Schier A. C. Taatjes D. J. (2020). Structure and mechanism of the RNA polymerase II transcription machinery. Genes Dev. 34 (7–8), 465–488. 10.1101/gad.335679.119 32238450 PMC7111264

[B215] Sebastian-delaCruz M. Gonzalez-Moro I. Olazagoitia-Garmendia A. Castellanos-Rubio A. Santin I. (2021). The role of lncRNAs in gene expression regulation through mRNA stabilization. Non-Coding RNA 7 (1), 3. 10.3390/ncrna7010003 33466464 PMC7839045

[B216] Segal M. Slack F. J. (2020). Challenges identifying efficacious miRNA therapeutics for cancer. Expert Opin. Drug Discov. 15 (9), 987–992. 10.1080/17460441.2020.1765770 32421364 PMC7415578

[B217] Sehovic E. Urru S. Chiorino G. Doebler P. (2022). Meta-analysis of diagnostic cell-free circulating microRNAs for breast cancer detection. BMC Cancer 22 (1), 634. 10.1186/s12885-022-09698-8 35681127 PMC9178880

[B218] Seliger B. Jasinski-Bergner S. Gonschorek E. Teren M. Stoehr C. Meinhardt A. (2014). Role of miRs in immune suppression. J. Immunother. Cancer 2, P268. 10.1186/2051-1426-2-s3-p268

[B219] Senthil Kumar K. Gokila V. M. Hsieh H. W. Lin C. C. Liao J. W. Chueh P. J. (2019). MicroRNA-708 activation by glucocorticoid receptor agonists regulate breast cancer tumorigenesis and metastasis via downregulation of NF-κB signaling. Carcinogenesis 40 (2), 335–348. 10.1093/carcin/bgz011 30726934

[B220] Sharma S. Nagpal N. Ghosh P. C. Kulshreshtha R. (2017). P53-miR-191-SOX4 regulatory loop affects apoptosis in breast cancer. Rna 23 (8), 1237–1246. 10.1261/rna.060657.117 28450532 PMC5513068

[B221] Sharma S. Opyrchal M. Lu X. (2022a). Harnessing tumorous flaws for immune supremacy: is miRNA-155 the weak link in breast cancer progression? J. Clin. Invest. 132 (19), e163010. 10.1172/JCI163010 36189796 PMC9525109

[B222] Sharma Y. Saini A. K. Kashyap S. Chandan G. Kaur N. Gupta V. K. (2022b). Host miRNA and immune cell interactions: relevance in nano-therapeutics for human health. Immunol. Res. 70 (1), 1–18. 10.1007/s12026-021-09247-8 34716546

[B223] Shen F. Cai W. S. Feng Z. Li J. L. Chen J. W. Cao J. (2015). MiR-492 contributes to cell proliferation and cell cycle of human breast cancer cells by suppressing SOX7 expression. Tumor Biol. 36, 1913–1921. 10.1007/s13277-014-2794-z 25407488

[B224] Shen Z. Xu X. Lv L. Dai H. Chen J. Chen B. (2020). miR-21 overexpression promotes esophageal squamous cell carcinoma invasion and migration by repressing tropomyosin 1. Gastroenterol. Res. Pract. 2020, 6478653. 10.1155/2020/6478653 33193757 PMC7641708

[B225] Shi P. Chen C. Li X. Wei Z. Liu Z. Liu Y. (2019). MicroRNA-124 suppresses cell proliferation and invasion of triple negative breast cancer cells by targeting STAT3. Mol. Med. Rep. 19 (5), 3667–3675. 10.3892/mmr.2019.10044 30896795 PMC6472193

[B226] Shi S. Han L. Deng L. Zhang Y. Shen H. Gong T. (2014). Dual drugs (microRNA-34a and paclitaxel)-loaded functional solid lipid nanoparticles for synergistic cancer cell suppression. J. Control. Release 194, 228–237. 10.1016/j.jconrel.2014.09.005 25220161

[B227] Shirjang S. Mansoori B. Asghari S. Duijf P. H. G. Mohammadi A. Gjerstorff M. (2019). MicroRNAs in cancer cell death pathways: apoptosis and necroptosis. Free Radic. Biol. Med. 139, 1–15. 10.1016/j.freeradbiomed.2019.05.017 31102709

[B228] Shukla K. Sharma A. K. Ward A. Will R. Hielscher T. Balwierz A. (2015). MicroRNA-30c-2-3p negatively regulates NF-κB signaling and cell cycle progression through downregulation of TRADD and CCNE1 in breast cancer. Mol. Oncol. 9 (6), 1106–1119. 10.1016/j.molonc.2015.01.008 25732226 PMC5528752

[B229] Si M. Zhu S. Wu H. Lu Z. Wu F. Mo Y. (2007). miR-21-mediated tumor growth. Oncogene 26 (19), 2799–2803. 10.1038/sj.onc.1210083 17072344

[B230] Skafi N. Fayyad-Kazan M. Badran B. (2020). Immunomodulatory role for MicroRNAs: regulation of PD-1/PD-L1 and CTLA-4 immune checkpoints expression. Gene 754, 144888. 10.1016/j.gene.2020.144888 32544493

[B231] Smolarz B. Durczyński A. Romanowicz H. Szyłło K. Hogendorf P. (2022). miRNAs in cancer (review of literature). Int. J. Mol. Sci. 23 (5), 2805. 10.3390/ijms23052805 35269947 PMC8910953

[B232] Song B. Hou G. Xu M. Chen M. (2024). Exosomal miR-122-3p represses the growth and metastasis of MCF-7/ADR cells by targeting GRK4-mediated activation of the Wnt/β-catenin pathway. Cell Signal 117, 111101. 10.1016/j.cellsig.2024.111101 38365112

[B233] Song C. Xiao Y. Ouyang Z. Shen M. Shi X. (2020). Efficient co-delivery of microRNA 21 inhibitor and doxorubicin to cancer cells using core–shell tecto dendrimers formed via supramolecular host–guest assembly. J. Mater Chem. B 8 (14), 2768–2774. 10.1039/d0tb00346h 32154812

[B234] Song Y. K. Wang Y. Wen Y. Y. Zhao P. Bian Z. J. (2018). RETRACTED: MicroRNA-22 suppresses breast cancer cell growth and increases paclitaxel sensitivity by targeting NRAS. Technol. Cancer Res. Treat. 17, 1533033818809997. 10.1177/1533033818809997 30384806 PMC6259065

[B235] Speiser D. E. Verdeil G. (2017). More T cells versus better T cells in patients with breast cancer. Cancer Discov. 7 (10), 1062–1064. 10.1158/2159-8290.CD-17-0858 28974529

[B236] Stenvang J. Petri A. Lindow M. Obad S. Kauppinen S. (2012). Inhibition of microRNA function by antimiR oligonucleotides. Silence 3 (1), 1–17. 10.1186/1758-907X-3-1 22230293 PMC3306207

[B237] Sui Y. Zhang X. Yang H. Wei W. Wang M. (2022). [Retracted] MicroRNA‑133a acts as a tumour suppressor in breast cancer through targeting LASP1. Oncol. Rep. 48 (1), 119. 10.3892/or.2022.8330 35583001 PMC9164267

[B238] Sun L. H. Tian D. Yang Z. C. Li J. L. (2020). Exosomal miR-21 promotes proliferation, invasion and therapy resistance of colon adenocarcinoma cells through its target PDCD4. Sci. Rep. 10 (1), 8271. 10.1038/s41598-020-65207-6 32427870 PMC7237414

[B239] Sun X. Xu H. Huang T. Zhang C. Wu J. Luo S. (2021). Simultaneous delivery of anti-miRNA and docetaxel with supramolecular self-assembled “chitosome” for improving chemosensitivity of triple negative breast cancer cells. Drug Deliv. Transl. Res. 11, 192–204. 10.1007/s13346-020-00779-4 32394334

[B240] Sun Y. S. Zhao Z. Yang Z. N. Xu F. Lu H. J. Zhu Z. Y. (2017). Risk factors and preventions of breast cancer. Int. J. Biol. Sci. 13 (11), 1387–1397. 10.7150/ijbs.21635 29209143 PMC5715522

[B241] Sung H. Ferlay J. Siegel R. L. Laversanne M. Soerjomataram I. Jemal A. (2021). Global cancer statistics 2020: GLOBOCAN estimates of incidence and mortality worldwide for 36 cancers in 185 countries. CA Cancer J. Clin. 71 (3), 209–249. 10.3322/caac.21660 33538338

[B242] Świętek A. Gołąbek K. Hudy D. Gaździcka J. Biernacki K. Miśkiewicz-Orczyk K. (2023). The potential association between E2F2, MDM2 and p16 protein concentration and selected sociodemographic and clinicopathological characteristics of patients with oral squamous cell carcinoma. Curr. Issues Mol. Biol. 45 (4), 3268–3278. 10.3390/cimb45040213 37185737 PMC10137059

[B243] Syeda S. Rawat K. Shrivastava A. (2022). Pharmacological inhibition of exosome machinery: an emerging prospect in cancer therapeutics. Curr. Cancer Drug Targets 22 (7), 560–576. 10.2174/1568009622666220401093316 35366773

[B244] Tabatabaei S. N. Derbali R. M. Yang C. Superstein R. Hamel P. Chain J. L. (2019). Co-delivery of miR-181a and melphalan by lipid nanoparticles for treatment of seeded retinoblastoma. J. Control. Release 298, 177–185. 10.1016/j.jconrel.2019.02.014 30776396

[B245] Taghavipour M. Sadoughi F. Mirzaei H. Yousefi B. Moazzami B. Chaichian S. (2020). Apoptotic functions of microRNAs in pathogenesis, diagnosis, and treatment of endometriosis. Cell Biosci. 10 (1), 12. 10.1186/s13578-020-0381-0 32082539 PMC7014775

[B246] Takaki S. Eto K. (2018). Cytoplasmic localization of programmed cell death 4 contributes to its anti-apoptotic function. Mol. Cell Biochem. 448 (1–2), 155–164. 10.1007/s11010-018-3322-z 29442268

[B247] Tan K. Naylor M. J. (2022). Tumour microenvironment-immune cell interactions influencing breast cancer heterogeneity and disease progression. Front. Oncol. 12, 876451. 10.3389/fonc.2022.876451 35646658 PMC9138702

[B248] Tang L. Wei D. Xu X. Mao X. Mo D. Yan L. (2021). Long non-coding RNA MIR200CHG promotes breast cancer proliferation, invasion, and drug resistance by interacting with and stabilizing YB-1. NPJ Breast Cancer 7 (1), 94. 10.1038/s41523-021-00293-x 34272387 PMC8285504

[B249] Tang W. W. Bauer K. M. Barba C. Ekiz H. A. O’Connell R. M. (2022). miR-aculous new avenues for cancer immunotherapy. Front. Immunol. 13, 929677. 10.3389/fimmu.2022.929677 36248881 PMC9554277

[B250] Taylor H. Laurence A. D. J. Uhlig H. H. (2019). The role of PTEN in innate and adaptive immunity. Cold Spring Harb. Perspect. Med. 9 (12), a036996. 10.1101/cshperspect.a036996 31501268 PMC6886458

[B251] Teplyuk N. M. Uhlmann E. J. Gabriely G. Volfovsky N. Wang Y. Teng J. (2016). Therapeutic potential of targeting micro RNA‐10b in established intracranial glioblastoma: first steps toward the clinic. EMBO Mol. Med. 8 (3), 268–287. 10.15252/emmm.201505495 26881967 PMC4772951

[B252] Testa U. Pelosi E. Castelli G. Labbaye C. (2017). miR-146 and miR-155: two key modulators of immune response and tumor development. Non-Coding RNA 3 (3), 22. 10.3390/ncrna3030022 29657293 PMC5831915

[B253] Thomopoulou K. Papadaki C. Monastirioti A. Koronakis G. Mala A. Kalapanida D. (2021). MicroRNAs regulating tumor immune response in the prediction of the outcome in patients with breast cancer. Front. Mol. Biosci. 8, 668534. 10.3389/fmolb.2021.668534 34179081 PMC8220200

[B254] Tiberio P. Callari M. Angeloni V. Daidone M. G. Appierto V. (2015). Challenges in using circulating miRNAs as cancer biomarkers. Biomed. Res. Int. 2015, 731479. 10.1155/2015/731479 25874226 PMC4385632

[B255] Tormo E. Adam-Artigues A. Ballester S. Pineda B. Zazo S. González-Alonso P. (2017). The role of miR-26a and miR-30b in HER2+ breast cancer trastuzumab resistance and regulation of the CCNE2 gene. Sci. Rep. 7 (1), 41309. 10.1038/srep41309 28120942 PMC5264595

[B256] Treiber T. Treiber N. Meister G. (2019). Regulation of microRNA biogenesis and its crosstalk with other cellular pathways. Nat. Rev. Mol. Cell Biol. 20 (1), 5–20. 10.1038/s41580-018-0059-1 30228348

[B257] Tzavlaki K. Moustakas A. (2020). TGF-Β signaling. Biomolecules 10 (3), 487. 10.3390/biom10030487 32210029 PMC7175140

[B258] Uz M. Kalaga M. Pothuraju R. Ju J. Junker W. M. Batra S. K. (2019). Dual delivery nanoscale device for miR-345 and gemcitabine co-delivery to treat pancreatic cancer. J. Control. Release 294, 237–246. 10.1016/j.jconrel.2018.12.031 30576747 PMC6379902

[B259] Valcourt D. M. Day E. S. (2020). Dual regulation of miR-34a and Notch signaling in triple-negative breast cancer by antibody/miRNA nanocarriers. Mol. Ther-Nucleic Acids 21, 290–298. 10.1016/j.omtn.2020.06.003 32622330 PMC7332498

[B260] van den Ende N. S. Smid M. Timmermans A. van Brakel J. B. Hansum T. Foekens R. (2022). HER2-low breast cancer shows a lower immune response compared to HER2-negative cases. Sci. Rep. 12, 12974. 10.1038/s41598-022-16898-6 35902644 PMC9334272

[B261] van Schooneveld E. Wildiers H. Vergote I. Vermeulen P. B. Dirix L. Y. Van Laere S. J. (2015). Dysregulation of microRNAs in breast cancer and their potential role as prognostic and predictive biomarkers in patient management. Breast Cancer Res. 17, 21–15. 10.1186/s13058-015-0526-y 25849621 PMC4332424

[B262] Van Zandwijk N. Pavlakis N. Kao S. C. Linton A. Boyer M. J. Clarke S. (2017). Safety and activity of microRNA-loaded minicells in patients with recurrent malignant pleural mesothelioma: a first-in-man, phase 1, open-label, dose-escalation study. Lancet Oncol. 18 (10), 1386–1396. 10.1016/S1470-2045(17)30621-6 28870611

[B263] Vautrin A. Manchon L. Garcel A. Campos N. Lapasset L. Laaref A. M. (2019). Both anti-inflammatory and antiviral properties of novel drug candidate ABX464 are mediated by modulation of RNA splicing. Sci. Rep. 9 (1), 792. 10.1038/s41598-018-37813-y 30692590 PMC6349857

[B264] Wang B. Zou A. Ma L. Chen X. Wang L. Zeng X. (2017a). miR-455 inhibits breast cancer cell proliferation through targeting CDK14. Eur. J. Pharmacol. 807, 138–143. 10.1016/j.ejphar.2017.03.016 28300591

[B265] Wang F. Zhang L. Bai X. Cao X. Jiao X. Huang Y. (2018b). Stimuli-responsive nanocarrier for co-delivery of MiR-31 and doxorubicin to suppress high MtEF4 cancer. ACS Appl. Mater Interfaces 10 (26), 22767–22775. 10.1021/acsami.8b07698 29897733

[B266] Wang H. Li X. Li T. Wang L. Wu X. Liu J. (2019). Multiple roles of microRNA-146a in immune responses and hepatocellular carcinoma. Oncol. Lett. 18 (5), 5033–5042. 10.3892/ol.2019.10862 31612014 PMC6781720

[B267] Wang J. Wang Q. Guan Y. Sun Y. Wang X. Lively K. (2022a). Breast cancer cell–derived microRNA-155 suppresses tumor progression via enhancing immune cell recruitment and antitumor function. J. Clin. Invest. 132 (19), e157248. 10.1172/JCI157248 35925680 PMC9525116

[B268] Wang J. Wang Q. Guan Y. Sun Y. Wang X. Lively K. (2022b). Breast cancer cell–derived microRNA-155 suppresses tumor progression via enhancing immune cell recruitment and antitumor function. J. Clin. Invest. 132 (19), e157248. 10.1172/JCI157248 35925680 PMC9525116

[B269] Wang J. Zeng H. Li H. Chen T. Wang L. Zhang K. (2017b). MicroRNA-101 inhibits growth, proliferation and migration and induces apoptosis of breast cancer cells by targeting sex-determining region Y-Box 2. Cell Physiol. Biochem. 43 (2), 717–732. 10.1159/000481445 28946143

[B270] Wang L. Liang T.-T. (2020). CD59 receptor targeted delivery of miRNA-1284 and cisplatin-loaded liposomes for effective therapeutic efficacy against cervical cancer cells. Amb. Express 10, 54. 10.1186/s13568-020-00990-z 32185543 PMC7078418

[B271] Wang M. Ji S. Shao G. Zhang J. Zhao K. Wang Z. (2018a). Effect of exosome biomarkers for diagnosis and prognosis of breast cancer patients. Clin. Transl. Oncol. 20, 906–911. 10.1007/s12094-017-1805-0 29143228

[B272] Wang Q. Yang H. S. (2018). The role of Pdcd4 in tumour suppression and protein translation. Biol. Cell. 28, 169–177. 10.1111/boc.201800014 PMC6261700 29806708

[B273] Wang S. Zhang J. Wang Y. Chen M. (2016). Hyaluronic acid-coated PEI-PLGA nanoparticles mediated co-delivery of doxorubicin and miR-542-3p for triple negative breast cancer therapy. Nanomedicine 12, 411–420. 10.1016/j.nano.2015.09.014 26711968

[B274] Wang X. Qiu W. Zhang G. Xu S. Gao Q. Yang Z. (2015). MicroRNA-204 targets JAK2 in breast cancer and induces cell apoptosis through the STAT3/BCl-2/survivin pathway. Int. J. Clin. Exp. Pathol. 8 (5), 5017–5025. 26191195 PMC4503067

[B275] Wei S. Gao J. Zhang M. Dou Z. Li W. Zhao L. (2020). Dual delivery nanoscale device for miR-451 and adriamycin co-delivery to combat multidrug resistant in bladder cancer. Biomed. Pharmacother. 122, 109473. 10.1016/j.biopha.2019.109473 31918263

[B276] Wong J. S. Cheah Y. K. (2020). Potential miRNAs for miRNA-based therapeutics in breast cancer. Non-Coding RNA 6 (3), 29. 10.3390/ncrna6030029 32668603 PMC7549352

[B277] Wu J. Lu P. Yang T. Wang L. (2014). Meta-analysis of the differentially expressed breast cancer-related microRNA expression profiles. J. Obstet. Gynaecol. 34 (7), 630–633. 10.3109/01443615.2014.920782 24922277

[B278] Wu Z. Cai X. Huang C. Xu J. Liu A. (2016). miR-497 suppresses angiogenesis in breast carcinoma by targeting HIF-1α. Oncol. Rep. 35 (3), 1696–1702. 10.3892/or.2015.4529 26718330

[B279] Xie D. Song H. Wu T. Li D. Hua K. Xu H. (2018). MicroRNA-424 serves an anti-oncogenic role by targeting cyclin-dependent kinase 1 in breast cancer cells. Oncol. Rep. 40 (6), 3416–3426. 10.3892/or.2018.6741 30272324 PMC6196586

[B280] Xie F. Hosany S. Zhong S. Jiang Y. Zhang F. Lin L. (2017). MicroRNA-193a inhibits breast cancer proliferation and metastasis by downregulating WT1. PloS One 12 (10), e0185565. 10.1371/journal.pone.0185565 29016617 PMC5634539

[B281] Xu J. Sun J. Ho P. Y. Luo Z. Ma W. Zhao W. (2019). Creatine based polymer for codelivery of bioengineered MicroRNA and chemodrugs against breast cancer lung metastasis. Biomaterials 210, 25–40. 10.1016/j.biomaterials.2019.04.025 31054369 PMC6538300

[B282] Yan C. Chen Y. Kong W. Fu L. Liu Y. Yao Q. (2017). PVT 1‐derived miR‐1207‐5p promotes breast cancer cell growth by targeting STAT 6. Cancer Sci. 108 (5), 868–876. 10.1111/cas.13212 28235236 PMC5448618

[B283] Yang L. Cai Y. Zhang D. Sun J. Xu C. Zhao W. (2018a). The open issues regarding cyclin-dependent kinase 4/6 inhibitors in the management of advanced breast cancer. J. Breast Cancer 21 (4), 468–470. 10.4048/jbc.2018.21.e59 30607170 PMC6310729

[B284] Yang L. Cai Y. Zhang D. Sun J. Xu C. Zhao W. (2018b). miR-195/miR-497 regulate CD274 expression of immune regulatory ligands in triple-negative breast cancer. J. Breast Cancer 21 (4), 371–381. 10.4048/jbc.2018.21.e60 30607158 PMC6310715

[B285] Yang T. Zhao P. Rong Z. Li B. Xue H. You J. (2016). Anti-tumor efficiency of lipid-coated cisplatin nanoparticles Co-loaded with MicroRNA-375. Theranostics 6, 142–154. 10.7150/thno.13130 26722380 PMC4679361

[B286] Yang X. Shang P. Yu B. Jin Q. Liao J. Wang L. (2021). Combination therapy with miR34a and doxorubicin synergistically inhibits Dox-resistant breast cancer progression via down-regulation of Snail through suppressing Notch/NF-κB and RAS/RAF/MEK/ERK signaling pathway. Acta Pharm. Sin. B 11 (9), 2819–2834. 10.1016/j.apsb.2021.06.003 34589399 PMC8463267

[B287] Yao C. Liu J. Wu X. Tai Z. Gao Y. Zhu Q. (2016). Reducible self-assembling cationic polypeptide-based micelles mediate co-delivery of doxorubicin and microRNA-34a for androgen-independent prostate cancer therapy. J. Control. Release 232, 203–214. 10.1016/j.jconrel.2016.04.034 27126903

[B288] Yin H. Xiong G. Guo S. Xu C. Xu R. Guo P. (2019). Delivery of anti-miRNA for triple-negative breast cancer therapy using RNA nanoparticles targeting stem cell marker CD133. Mol. Ther. 27 (7), 1252–1261. 10.1016/j.ymthe.2019.04.018 31085078 PMC6612664

[B289] Yoon A. J. Wang S. Kutler D. I. Carvajal R. D. Philipone E. Wang T. (2020). MicroRNA-based risk scoring system to identify early-stage oral squamous cell carcinoma patients at high-risk for cancer-specific mortality. Head. Neck 42 (8), 1699–1712. 10.1002/hed.26089 31981257 PMC7369212

[B290] Zacharias F. George D. Michail D. Ioannis P. Marianna T. Arzou B. (2020). MicroRNAs determining carcinogenesis by regulating oncogenes and tumor suppressor genes during cell cycle. MicroRNA 9 (2), 82–92. 10.2174/2211536608666190919161849 31538910 PMC7366009

[B291] Zhan M. N. Yu X. T. Tang J. Zhou C. X. Wang C. L. Yin Q. Q. (2018). MicroRNA-494 inhibits breast cancer progression by directly targeting PAK1. Cell Death Dis. 8 (1), e2529. 10.1038/cddis.2016.440 PMC5386359 28055013

[B292] Zhang G. Zhang W. Li B. Stringer-Reasor E. Chu C. Sun L. (2017b). MicroRNA-200c and microRNA-141 are regulated by a FOXP3-KAT2B axis and associated with tumor metastasis in breast cancer. Breast Cancer Res. 19, 73–13. 10.1186/s13058-017-0858-x 28637482 PMC5480201

[B293] Zhang H. Li M. Kaboli P. J. Ji H. Du F. Wu X. (2021b). Identification of cluster of differentiation molecule-associated microRNAs as potential therapeutic targets for gastrointestinal cancer immunotherapy. Int. J. Biol. Markers. 36 (2), 22–32. 10.1177/17246008211005473 33788641

[B294] Zhang J. Liu D. Feng Z. Mao J. Zhang C. Lu Y. (2015a). MicroRNA-138 modulates metastasis and EMT in breast cancer cells by targeting vimentin. Biomed. Pharmacother. Biomedecine Pharmacother. 77, 135–141. 10.1016/j.biopha.2015.12.018 26796277

[B295] Zhang J. Shan W. F. Jin T. T. Wu G. Q. xing X. X. yan J. H. (2014b). Propofol exerts anti-hepatocellular carcinoma by microvesicle-mediated transfer of miR-142-3p from macrophage to cancer cells. J. Transl. Med. 12, 279–9. 10.1186/s12967-014-0279-x 25292173 PMC4198740

[B296] Zhang J. Zhang Z. Wang Q. Xing X. J. Zhao Y. (2016). Overexpression of microRNA-365 inhibits breast cancer cell growth and chemo-resistance through GALNT4. Eur. Rev. Med. Pharmacol. Sci. 20 (22), 4710–4718. 27906431

[B297] Zhang K. Wang Y. Y. Xu Y. Zhang L. Zhu J. Si P. C. (2021a). A two-miRNA signature of upregulated miR-185-5p and miR-362-5p as a blood biomarker for breast cancer. Pathol-Res Pract. 222, 153458. 10.1016/j.prp.2021.153458 33962174

[B298] Zhang K. Zhang Y. Liu C. Xiong Y. Zhang J. (2014a). MicroRNAs in the diagnosis and prognosis of breast cancer and their therapeutic potential (review). Int. J. Oncol. 45 (3), 950–958. 10.3892/ijo.2014.2487 24913679

[B299] Zhang L. Yang X. Lv Y. Xin X. Qin C. Han X. (2017a). Cytosolic co-delivery of miRNA-34a and docetaxel with core-shell nanocarriers via caveolae-mediated pathway for the treatment of metastatic breast cancer. Sci. Rep. 7 (1), 46186. 10.1038/srep46186 28383524 PMC5382875

[B300] Zhang M. Gao D. Shi Y. Wang Y. Joshi R. Yu Q. (2019). miR-149-3p reverses CD8+ T-cell exhaustion by reducing inhibitory receptors and promoting cytokine secretion in breast cancer cells. Open Biol. 9 (10), 190061. 10.1098/rsob.190061 31594465 PMC6833224

[B301] Zhang M. Shi Y. Zhang Y. Wang Y. Alotaibi F. Qiu L. (2020b). miRNA-5119 regulates immune checkpoints in dendritic cells to enhance breast cancer immunotherapy. Cancer Immunol. Immunother. 69, 951–967. 10.1007/s00262-020-02507-w 32076794 PMC11027689

[B302] Zhang Q. Ran R. Zhang L. Liu Y. Mei L. Zhang Z. (2015b). Simultaneous delivery of therapeutic antagomirs with paclitaxel for the management of metastatic tumors by a pH-responsive anti-microbial peptide-mediated liposomal delivery system. J. Control. Release 197, 208–218. 10.1016/j.jconrel.2014.11.010 25445692

[B303] Zhang Y. Xiao Y. Ma Y. Liang N. Liang Y. Lu C. (2020a). ROS-mediated miR-21-5p regulates the proliferation and apoptosis of Cr(VI)-exposed L02 hepatocytes via targeting PDCD4. Ecotoxicol. Environ. Saf. 191, 110160. 10.1016/j.ecoenv.2019.110160 31951899

[B304] Zhang Y. Yan J. Wang L. Dai H. Li N. Hu W. (2017c). HIF-1α promotes breast cancer cell MCF-7 proliferation and invasion through regulating miR-210. Cancer Biother Radiopharm. 32 (8), 297–301. 10.1089/cbr.2017.2270 29053417

[B305] Zhao Y. Yang F. Li W. Xu C. Li L. Chen L. (2017). miR-29a suppresses MCF-7 cell growth by downregulating tumor necrosis factor receptor 1. Tumor Biol. 39 (2), 1010428317692264. 10.1177/1010428317692264 28222663

[B306] Zhao Z. Li L. Du P. Ma L. Zhang W. Zheng L. (2019). Transcriptional downregulation of miR-4306 serves as a new therapeutic target for triple negative breast cancer. Theranostics 9 (5), 1401–1416. 10.7150/thno.30701 30867840 PMC6401504

[B307] Zhi F. Dong H. Jia X. Guo W. Lu H. Yang Y. (2013). Functionalized graphene oxide mediated adriamycin delivery and miR-21 gene silencing to overcome tumor multidrug resistance *in vitro* . PloS One 8 (3), e60034. 10.1371/journal.pone.0060034 23527297 PMC3603917

[B308] Zhirong Z. Qiaojian Z. Chunjing X. Shengchen W. Jiahe L. Zhaoyi L. (2021). Methionine selenium antagonizes LPS-induced necroptosis in the chicken liver via the miR-155/TRAF3/MAPK axis. J. Cell Physiol. 236 (5), 4024–4035. 10.1002/jcp.30145 33151563

[B309] Zhou H. Jia W. Lu L. Han R. (2023a). MicroRNAs with multiple targets of immune checkpoints, as a potential sensitizer for immune checkpoint inhibitors in breast cancer treatment. Cancers 15 (3), 824. 10.3390/cancers15030824 36765782 PMC9913694

[B310] Zhou H. Jia W. Lu L. Han R. (2023b). MicroRNAs with multiple targets of immune checkpoints, as a potential sensitizer for immune checkpoint inhibitors in breast cancer treatment. Cancers 15 (3), 824. 10.3390/cancers15030824 36765782 PMC9913694

[B311] Zhou L. Dong J. Huang G. Sun Z. Wu J. (2017a). MicroRNA-143 inhibits cell growth by targeting ERK5 and MAP3K7 in breast cancer. Braz J. Med. Biol. Res. 50, e5891. 10.1590/1414-431X20175891 28746466 PMC5520219

[B312] Zhou Z. Kennell C. Lee J.-Y. Leung Y.-K. Tarapore P. (2017b). Calcium phosphate-polymer hybrid nanoparticles for enhanced triple negative breast cancer treatment via co-delivery of paclitaxel and miR-221/222 inhibitors. Nanomedicine 13, 403–410. 10.1016/j.nano.2016.07.016 27520723

